# Crystal structure and catalytic mechanism of the MbnBC holoenzyme required for methanobactin biosynthesis

**DOI:** 10.1038/s41422-022-00620-2

**Published:** 2022-02-02

**Authors:** Chao Dou, Zhaolin Long, Shoujie Li, Dan Zhou, Ying Jin, Li Zhang, Xuan Zhang, Yanhui Zheng, Lin Li, Xiaofeng Zhu, Zheng Liu, Siyu He, Weizhu Yan, Lulu Yang, Jie Xiong, Xianghui Fu, Shiqian Qi, Haiyan Ren, She Chen, Lunzhi Dai, Binju Wang, Wei Cheng

**Affiliations:** 1 Division of Respiratory and Critical Care Medicine, Respiratory Infection and Intervention Laboratory of Frontiers Science Center for Disease-related Molecular Network, State Key Laboratory of Biotherapy, West China Hospital of Sichuan University, Chengdu, Sichuan China; 2 State Key Laboratory of Structural Chemistry of Solid Surface and Fujian Provincial Key Laboratory of Theoretical and Computational Chemistry, College of Chemistry and Chemical Engineering, Xiamen University, Xiamen, Fujian China; 3 National Institute of Biological Sciences, NIBS, Beijing, China; 4 College of Life Science, Sichuan University, Chengdu, Sichuan China; 5 Beijing National Laboratory for Molecular Sciences (BNLMS), Beijing Key Laboratory for Magnetoelectric Materials and Devices, College of Chemistry and Molecular Engineering, Peking University, Beijing, China

**Keywords:** X-ray crystallography, Post-translational modifications

## Abstract

Methanobactins (Mbns) are a family of copper-binding peptides involved in copper uptake by methanotrophs, and are potential therapeutic agents for treating diseases characterized by disordered copper accumulation. Mbns are produced via modification of MbnA precursor peptides at cysteine residues catalyzed by the core biosynthetic machinery containing MbnB, an iron-dependent enzyme, and MbnC. However, mechanistic details underlying the catalysis of the MbnBC holoenzyme remain unclear. Here, we present crystal structures of MbnABC complexes from two distinct species, revealing that the leader peptide of the substrate MbnA binds MbnC for recruitment of the MbnBC holoenzyme, while the core peptide of MbnA resides in the catalytic cavity created by the MbnB–MbnC interaction which harbors a unique tri-iron cluster. Ligation of the substrate sulfhydryl group to the tri-iron center achieves a dioxygen-dependent reaction for oxazolone-thioamide installation. Structural analysis of the MbnABC complexes together with functional investigation of MbnB variants identified a conserved catalytic aspartate residue as a general base required for MbnBC-mediated MbnA modification. Together, our study reveals the similar architecture and function of MbnBC complexes from different species, demonstrating an evolutionarily conserved catalytic mechanism of the MbnBC holoenzymes.

## Introduction

Metals such as copper are critical in maintaining physiological homeostasis in all living organisms,^1^ and are specifically involved in catalysis of some essential bacterial proteins.^2–6^ While copper is necessary for certain protein activities, it is also employed as an antibacterial agent in multiple industrial and medical fields.^7^ In response, bacteria can use copper-chelating compounds such as chalkophores for detoxification. Chalkophore molecules are similar to the iron-binding siderophores involved in bacterial metabolism and detoxification.^8–10^ Notably, they have been investigated in clinical trials as potential therapeutic agents for Wilson disease, a genetic disorder that causes excessive copper accumulation in organs such as the liver and brain.^11–14^

Methanobactins (Mbns) are chalkophores present specifically in methanotrophs.^15,16^ A few Mbns have been characterized^15,17–20^ (Supplementary information, Fig. S1a) and found to be ribosomally synthesized and posttranslationally modified peptides (RiPPs).^17,21–23^ Mbn molecules are biosynthesized from gene-encoded precursor polypeptides (MbnAs) containing a leader peptide (LP) for biosynthetic machinery recognition and a core peptide (CP) that becomes the mature product after MbnA modification and LP cleavage^21,22^ (Supplementary information, Fig. S1b, c). A core biosynthetic machinery containing the MbnBC complex was recently identified in *Methylosinus trichosporium* (*Mt*) OB3b.^17^

Five gene-encoded cluster groups (I–V) for Mbn biosynthesis have been characterized thus far^23^ (Supplementary information, Fig. S1d). Among the known Mbn operons, *mbnA* is always followed by *mbnB* and *mbnC* genes which are responsible for MbnA modification.^17,21^ *mbnB* is predicted to encode a triose phosphate isomerase (TIM),^24^ and shows MbnA-modifying activity only in complex with MbnC.^10,17^ However, the organization and catalytic mechanism of the MbnBC machinery remains unknown. Here, we determined the crystal structures of MbnBC complexes of *Rugamonas rubra* (*Rr*) ATCC 43154 (RrMbnBC, representing Group III) and *Vibrio caribbenthicus* (*Vc*) BAA-2122 (VcMbnBC, representing Group V) (Supplementary information, Table S1) bound to RrMbnA and VcMbnA, respectively. The structure of RrMbnABC and VcMbnABC is conserved, containing a unique tri-iron cluster ligated with the cysteine residue of substrate MbnA. Our findings elucidate the mechanism of Mbn catalytic production by the MbnBC holoenzyme.

## Results

### Reconstitution of MbnABC complexes

**Fig. 1:**
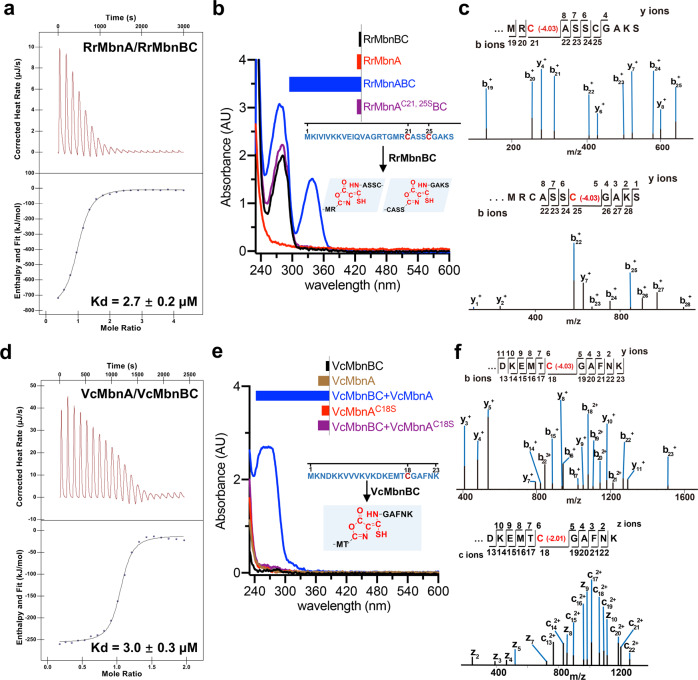
Reconstitution of MbnABC complexes. **a** ITC measurement of the binding affinity between RrMbnA and RrMbnBC. The upper panel shows the original titration traces. **b** UV-Vis spectra detection of RrMbnA and RrMbnA^C21,25S^ modifications catalyzed by co-expressed RrMbnBC with absorption peaks at ~270 nm and ~335 nm. The amino acid sequence of RrMbnA is indicated, and the potentially modified residues are shown in red. Putative products are shown as chemical structures. Inset, the activities of these proteins are shown by UV absorbance at 335 nm. **c** Analysis of the modified RrMbnA by electrospray ionization tandem MS (ESI-MS/MS). The mass changes of Cys21 or Cys25 are highlighted in red. **d** ITC measurement of the binding affinity between VcMbnA and VcMbnBC. **e** Absorbance of synthetic VcMbnA and variant VcMbnA^C18S^ modified by VcMbnBC. The peak absorbance of the modified VcMbnA is shown at ~270 nm. The amino acid sequence of VcMbnA is indicated, and the potentially modified cysteine is shown in red. Putative product is shown as a chemical structure. Inset, the activities of these proteins are shown by UV absorbance at 270 nm. **f** Analysis of the modified VcMbnA by ESI-MS/MS. The mass changes of Cys18 are highlighted in red.

To facilitate structural studies of MbnABC, we reconstituted its complexes from different species (Supplementary information, Fig. S1e). Similar to MtMbnABC,^17^ the three components of RrMbnABC formed a stable complex when co-expressed in *Escherichia coli* (Supplementary information, Fig. S2a). The interaction of RrMbnA with RrMbnBC was verified by isothermal titration calorimetry (ITC) (Fig. 1a). Consistently, limited proteolysis showed that RrMbnA binding could enhance RrMbnBC stability (Supplementary information, Fig. S2b). The conjugate bond in the modified MbnA can be detected using a scanning ultraviolet-visible (UV-Vis) spectrophotometer.^17,19^ Indeed, RrMbnA could be modified by RrMbnBC in the co-expressed system, and the product displayed absorption peak at ~335 nm, consistent with observations in MtMbnA^17^ (Fig. 1b). High-resolution mass spectrometry (MS) indicated that the absorption resulted from modifications on either Cys21 or Cys25 (Fig. 1c). Supporting this finding, a double mutation C21S/C25S completely abolished the absorption at this wavelength (Fig. 1b). Further supporting that RrMbnA was modified, MS data showed decreases of 2 and 4 Daltons in molecular weight of the peptide substrate (Fig. 1c; Supplementary information, Fig. S2c), which are characteristic mass shifts in modified MbnA.^17^

The aerobically purified VcMbnBC proteins interacted with VcMbnA as indicated by a pull-down assay and further confirmed by ITC (Fig. 1d; Supplementary information, Fig. S3a). Synthetic VcMbnA notably inhibited trypsin-mediated digestion of MbnBC, indicating that VcMbnA stabilizes the MbnBC complex (Supplementary information, Fig. S3b). Unexpectedly, the modified VcMbnA exhibited a strong absorption peak at ~270 nm (Fig. 1e) and a shoulder absorption in the range of 300–360 nm. However, the expected decreases of 2 and 4 Daltons in the molecular weight of the peptide were detected (Fig. 1f). Additionally, an increase of 14 and a decrease of 30 Daltons in the mass of VcMbnA were also detected with MS (Supplementary information, Fig. S3c). MS analysis further showed that all of the mass shifts occurred at the cysteine residue (Cys18) of VcMbnA, and the VcMbnA^C18S^ mutation completely abrogated absorption in the UV range (Fig. 1e). The modified VcMbnA with absorption at ~270 nm is reminiscent of acid-catalyzed hydrolysis of the oxazolone-thioamide-containing products^20,25–28^ (Supplementary information, Fig. S3d). Supporting this hypothesis, the MS data analysis showed that mass shifts +14 and −30 Daltons stemmed from the predicted intermediate and product generated by hydrolysis of the modified VcMbnA, respectively (Supplementary information, Fig. S3c, e). Our results here differ from the previous report^17^ likely because our assays were performed using synthetic substrate in vitro.

Apart from the absorption at 335 nm, the modified RrMbnA also displayed absorption at ~270 nm (Fig. 1b), and this absorption was promoted by acid-mediated hydrolysis. Supporting the idea that the absorption resulted from a modified RrMbnA, a mass shift of −30 Daltons in the modified RrMbnA^C21S/C25S^ was observed (Supplementary information, Fig. S3f). Importantly, ITC assays showed that Cu^2+^ bound to the modified RrMbnA and VcMbnA but not to their unmodified forms (Supplementary information, Fig. S4a, b). The characteristic of acid-mediated hydrolysis of modified VcMbnA differs from that of the modified RrMbnA; the latter is similar to that of previously studied Mbns^17^ (Supplementary information, Fig. S4c–e). This is likely because the modified VcMbnA was almost hydrolyzed (Supplementary information, Fig. S3c–e). Additionally, the enzymatic activities of anaerobically purified RrMbnBC and VcMbnBC proteins showed no notable improvement compared with those of aerobically purified ones (Supplementary information, Fig. S5a–c).

### Overall structures of MbnABCs

**Fig. 2:**
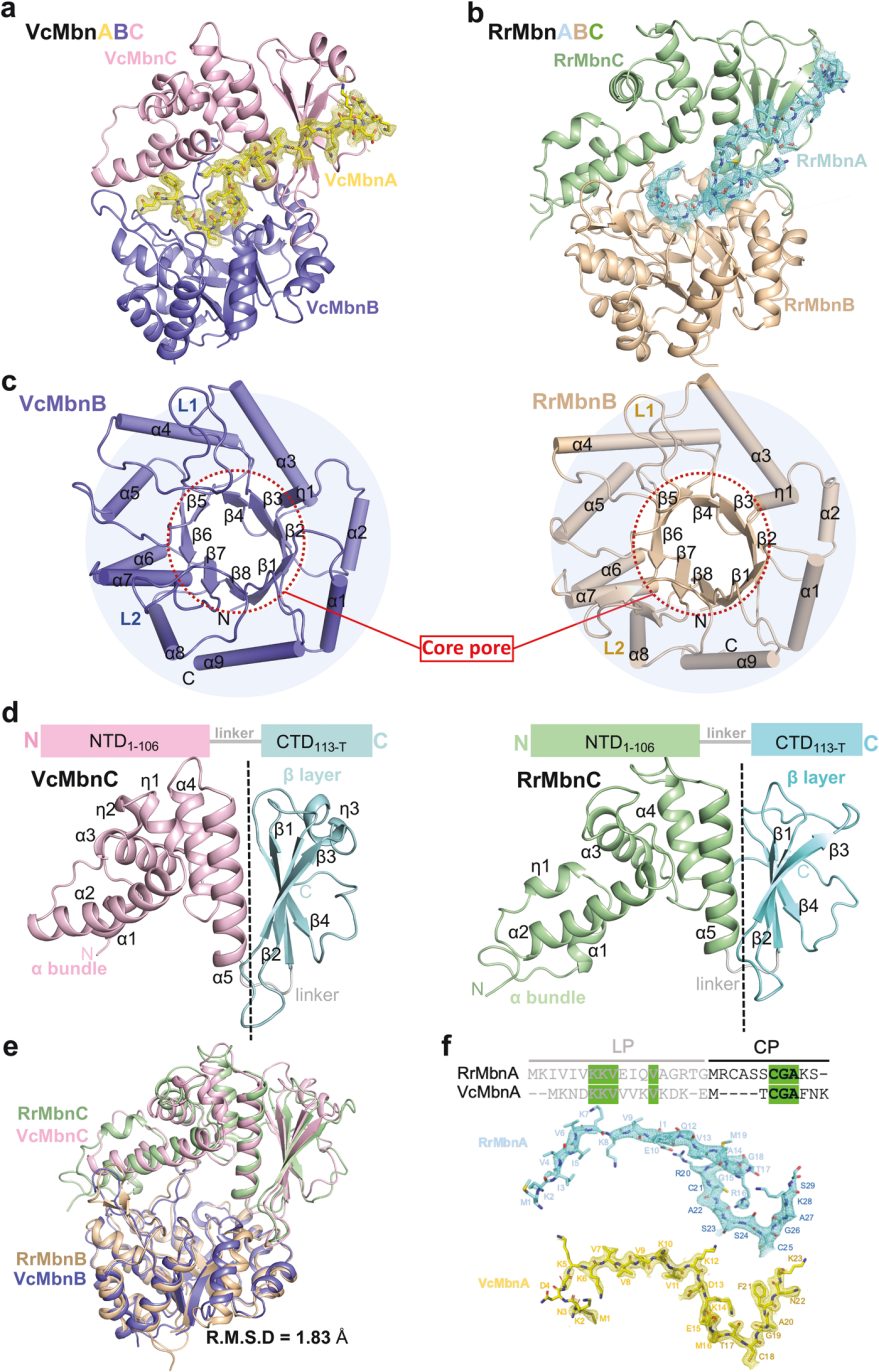
Structures of VcMbnABC and RrMbnABC. **a**–**b** Overall structures of MbnABC complexes, VcMbnABC (**a**), RrMbnABC (**b**). MbnA, MbnB and MbnC are shown in different colors. The MbnA shown as ball-and-stick models are contoured with a 2Fo-Fc map at 1 σ. **c** The TIM barrel of VcMbnB (left) and RrMbnB (right). Red dotted circles denote core pores. **d** The structure of VcMbnC (left) and RrMbnC (right). NTD and CTD are presented as cartoons. **e** VcMbnBC and RrMbnBC show nearly identical architectures. **f** The structures of RrMbnA (upper) and VcMbnA (lower), contoured with a 2Fo-Fc map at 1.5 σ. Sequence alignment of RrMbnA and VcMbnA is shown with conserved amino acids highlighted.

To understand the mechanism of how MbnBCs catalyze modification of MbnAs, we finally solved the crystal structures of VcMbnABC and RrMbnABC at resolutions of 2.2 Å and 2.7 Å, respectively. In both structures, the interactions between MbnA, MbnB and MbnC result in the formation of a 1:1:1 tertiary complex, and the overall structures of VcMbnABC and RrMbnABC are nearly identical (Fig. 2a, b; Supplementary information, Fig. S6a and Table S1). MbnB adopts a TIM barrel (α_9_β_8_) fold, comprising eight parallel β-sheets surrounded by nine α-helices (Fig. 2c), which resembles the unpublished crystal structure of a protein member of the DUF692 family in *Haemophilus somnus* (*Hs*), whose function remains unknown (hereafter named HsMbnB, PDB ID: 3BWW, Supplementary information, Fig. S6b, c). The structure of MbnC is elongated and can be divided into an N-terminal helix domain and a C-terminal β-sheet domain (Fig. 2d). Querying the DALI server^29^ failed to identify any structural homologs of MbnC, indicating that the structure represents a novel fold. The overall structures of VcMbnBC and RrMbnBC are very similar (Fig. 2e, root mean square deviations (RMSD) = 1.83 Å). Intriguingly, despite their divergent sequences (Fig. 2f), VcMbnA and RrMbnA assume similar conformations when they interact with VcMbnBC and RrMbnBC, respectively (Fig. 2f).

### Interactions between MbnB and MbnC

The secondary structure of the regions involved in the interaction between MbnB and MbnC are identical in VcMbnABC and RrMbnABC (Supplementary information, Fig. S6a). The N-terminal domain (NTD) of MbnC is sandwiched between two extended loops of MbnB (Supplementary information, Fig. S7a). One extended loop (L1) of MbnB packs against the horizontal helix (α1) of the MbnC NTD, whereas the other extended loop (L2) interacts with α2 and the two crossing α-helices from underneath. These interactions collectively form interface I between MbnB and MbnC. In addition, the hairpin loop between β2 and β3 and the loop linking the NTD and CTD of MbnC contact MbnB at the other side, forming the interface II (Supplementary information, Fig. S7b). Structural-based sequence alignment showed that the amino acids mediating interactions between VcMbnB and VcMbnC are highly conserved in RrMbnBC and other MbnBC complexes (Supplementary information, Fig. S7c). Collectively, our results suggest a conserved mechanism regulating the formation of MbnBC holoenzymes to produce Mbns.

To verify the MbnB–MbnC interactions observed in the crystal structures, we generated two VcMbnC mutants by deleting N-terminal residues 1–12 and an internal fragment containing residues 131–136, which mediate interactions within interfaces I and II, respectively. We then tested whether the VcMbnC mutants could still interact with VcMbnB. As expected, VcMbnC^131–136^ truncation resulted in the insolubility of VcMbnC and VcMbnC^△NTD12^ truncation abolished interaction with VcMbnB, indicating that the interaction interfaces are critical for the formation of the MbnBC complex (Supplementary information, Fig. S7d). This further supports the essentiality of these two regions in VcMbnB-mediated modification of VcMbnA. Similarly, no RrMbnBC complex could be obtained when equivalent regions of RrMbnC were mutated, as both RrMbnC^151–156^ and RrMbnC^△NTD28^ are insoluble (Supplementary information, Fig. S7e). The biochemical data thus confirmed the MbnB–MbnC interaction observed in the structure.

### MbnA recognition by the MbnBC complex

**Fig. 3:**
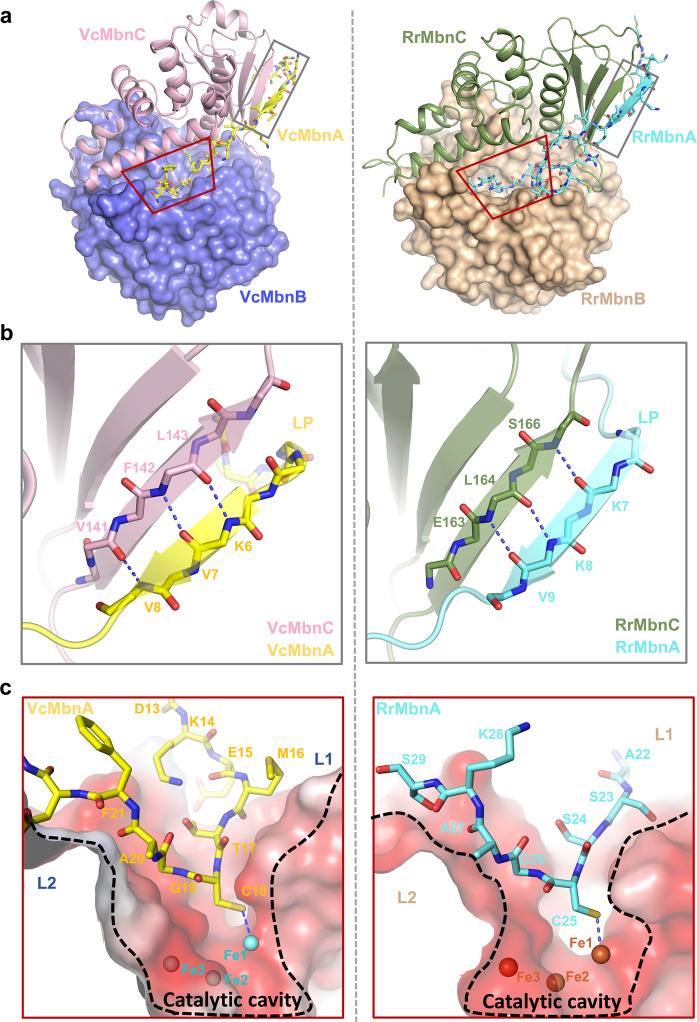
MbnA recognition by MbnBC. **a** MbnAs are shown in VcMbnABC (left) and RrMbnABC complexes (right). MbnA, MbnB and MbnC are shown as ball-and-stick, surface and cartoon representations, respectively. Interaction regions between MbnA and MbnBC are highlighted by colored boxes with LP-binding region in gray and CP-binding region in red. **b** Interaction of LP with MbnCs (left, VcMbnAC; right, RrMbnAC). Conserved residues are labeled and hydrogen bonds are indicated by blue dashed lines. **c** MbnA CP resides in the catalytic cavity of MbnB (left, VcMbnAB; right, RrMbnAB).

MbnA establishes extensive interactions with MbnBC, binding to both MbnC and MbnB and burying a surface area of ~1588 Å^2^ in VcMbnBC and ~1791 Å^2^ in RrMbnBC (Supplementary information, Fig. S8). In both complexes, a large majority (~978 Å^2^ for VcMbnABC and ~979 Å^2^ for RrMbnABC) of the buried surface is derived from contacts of MbnA with MbnC (Supplementary information, Fig. S8), explaining why MbnC is required for the substrate MbnA to interact with the catalytic subunit MbnB (Fig. 3a). The N-terminal half of the LP interacts predominantly with MbnC, forming an antiparallel β-sheet via three inter-main chain hydrogen bonds (Fig. 3b). Notably, similar binding was observed in the interactions between the RiPP leader peptide and the precursor peptide-recognition element (RRE)^30–33^ (Supplementary information, Fig. S9a–f). Structural prediction of MbnABC complexes of other groups with AlphaFold2^34,35^ further supports this conserved LP-binding model (Supplementary information, Fig. S9g, h). In VcMbnA, the C-terminal half of the LP (residues 13–16) and the CP (residues 17–23) form a curved structure that docks into the catalytic cavity (Fig. 3c). This presents the only cysteine residue (Cys18) of VcMbnA at the apex of the curve, coordinating with one of the iron ions in the catalytic cavity formed by VcMbnBC (Fig. 3c). A similar curved conformation is formed by the RrMbnA CP in the RrMbnABC complex (Fig. 3c). In contrast to VcMbnA, the C-terminal half of the RrMbnA LP (residues 15–20) forms a short kink, which interacts with the surface loop region of RrMbnB (Supplementary information, Fig. S10a). Formation of the kink allows the second cysteine residue (Cys25) to coordinate to one of the iron ions in the catalytic cavity of RrMbnBC.

Binding assays of VcMbnBC to VcMbnA with either LP or CP deleted indicated that VcMbnA with LP alone displayed weak interaction with VcMbnBC, whereas deletion of LP completely abolished interaction with VcMbnBC. These results show that LP and CP are cooperative in the interaction with VcMbnBC (Fig. 1d; Supplementary information, Fig. S10b). VcMbnA^C18S^ maintains the interaction but compromises VcMbnBC-mediated modification (Fig. 1d; Supplementary information, Fig. S10c). Collectively, our results suggest that MbnA recognition is similar to that of the RiPP leader peptide recognition involved in enzyme processing, although the regioselectivity and promiscuity remain unclear^30–33^.

### Substrate specificity determinants for the MbnBC holoenzymes

**Fig. 4:**
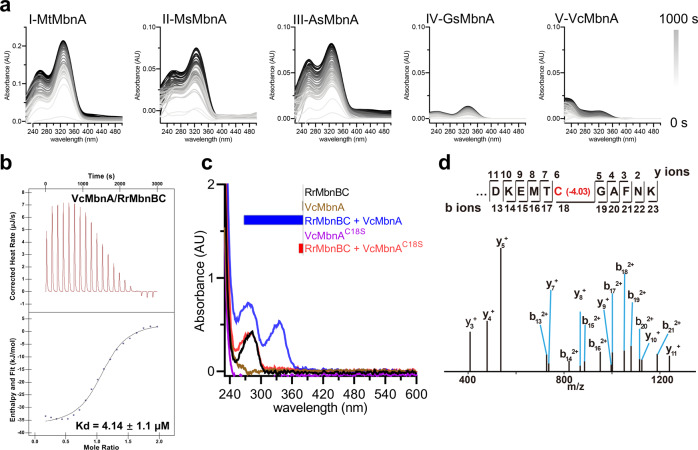
Promiscuous substrate recognition. **a** Activity assay of RrMbnBC with MbnAs from five Mbn groups over 1000 s. MbnAs from *Methylosinus trichosporium* OB3b (MtMbnA, Group I), *Methylocystis* sp. SC2 (MsMbnA, Group II), *Azospirillum* sp. B510 (AsMbnA, Group III), *Gluconacetobacter* sp. SXCC-1 (GsMbnA, Group IV), and *Vibrio caribbenthicus* BAA-2122 (VcMbnA, Group V) are used. **b** ITC measurement of the binding affinity between VcMbnA and RrMbnBC. **c** UV-Vis spectra detection for the synthetic VcMbnA and VcMbnA^C18S^ modified by RrMbnBC, with the absorption peaks at ~270 nm and ~335 nm. Inset, the activities of these proteins are shown by UV absorbance at ~335 nm. **d** ESI-MS/MS analysis of the VcMbnA modification catalyzed by RrMbnBC. The mass change of Cys18 is highlighted in red.

Among the MbnB and MbnC proteins from different species, VcMbnB and VcMbnC are distantly related to those in other species (Supplementary information, Fig. S11a, b). Nonetheless, the structures of VcMbnABC and RrMbnABC are nearly identical (Supplementary information, Fig. S6a), indicating that the MbnBC holoenzyme machinery for MbnA modification is conserved. Structural-based sequence alignment indicated that the MbnA recognition residues are conserved among MbnBC from different groups, particularly from Groups I–IV (Supplementary information, Fig. S11c). This may explain the substrate promiscuity of MbnBCs.^17^ Consistent with this hypothesis, our enzymatic assays showed that RrMbnBC was active in modifying MbnAs from Groups I–V (Fig. 4a). Notably, VcMbnA (Group V) could be recognized and modified by RrMbnBC (Fig. 4b, c); mass shifts in the VcMbnA^C18^ were verified by tandem MS (Fig. 4d; Supplementary information, Fig. S12a, b). However, despite the highly conserved structure, MbnBCs from Group V are specific for Group V MbnAs, as shown previously^17^ and further confirmed here (Supplementary information, Fig. S12c). Structural superposition showed that VcMbnA and RrMbnA form a set of conserved interactions with VcMbnBC and RrMbnBC, respectively (Supplementary information, Fig. S9g, h), providing further evidence for promiscuous substrate recognition. Some nonconserved residues of VcMbnB and VcMbnC form severe steric clashes with the kink region of RrMbnA, likely preventing recognition of RrMbnA or other MbnAs by VcMbnBC. Overall, the structural observations suggest that these nonconserved residues are likely to be determinants for the specific selection of VcMbnA by VcMbnBC.

### MbnB active site

**Fig. 5:**
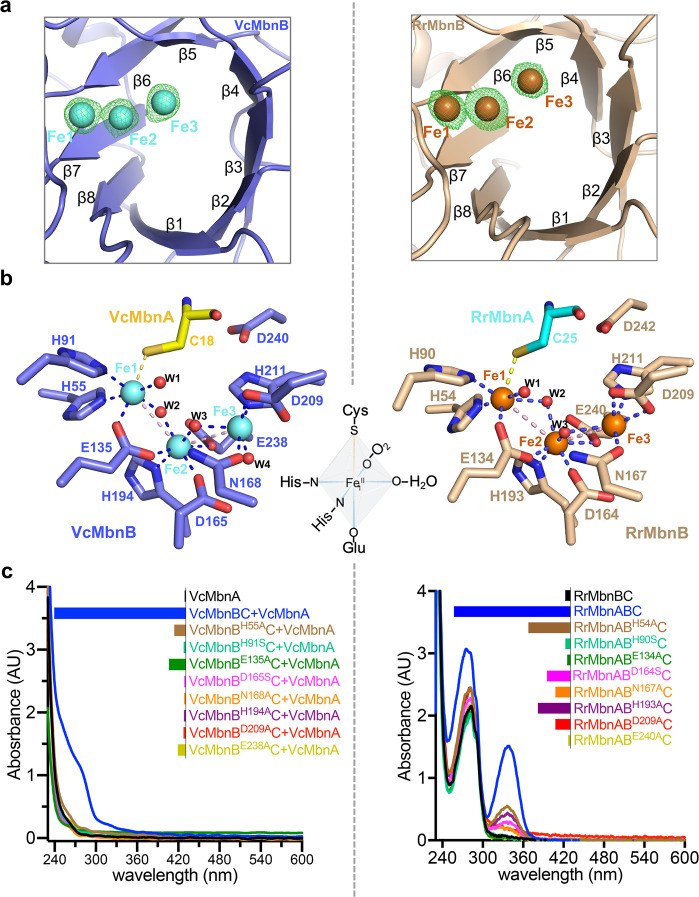
MbnB active site. **a** Electron density maps of three metal ions contoured with a 2Fo-Fc map at 5 σ in VcMbnB (left) and RrMbnB (right) structures. Fe atoms are colored in cyan (VcMbnB) and brown (RrMbnB). **b** Interaction networks formed by the tri-iron cluster with surrounding residues in VcMbnB (left) and RrMbnB (right). The six-coordinate Fe_1_^II^ is highlighted as a cartoon in the middle. O_2_ occupies a water site for activation. Hydrogen bonds, Fe–S bonds, and Fe–Fe bonds are indicated by blue, yellow and pink dashed lines, respectively. W, water molecule. **c** In vitro activity of VcMbnBC or mutants to modify the synthetic VcMbnA (left); in vivo activity of RrMbnBC or mutants to modify the co-expressed RrMbnA (right). The relative absorbances at ~270 nm for VcMbnA and ~335 nm for RrMbnA are represented as histograms in the insets.

Strong unoccupied electron densities at the catalytic sites of VcMbnABC and RrMbnABC suggest the existence of three metal ions (Fig. 5a). Supporting this conclusion, metal element analysis of the purified proteins with inductively coupled plasma mass spectrometry (ICP-MS) showed that RrMbnABC and VcMbnBC contain 2.80 ± 0.36 and 2.83 ± 0.19 iron ions per heterotrimer, respectively (Supplementary information, Table S2). Moreover, high spin Fe^III^ ions were detected by electron paramagnetic resonance spectroscopy (EPR) in the protein samples (Supplementary information, Fig. S13a). Importantly, ^57^Fe-Mössbauer spectroscopy of the anaerobically purified VcMbnBC and RrMbnBC proteins indicated a trinuclear cluster and a 2:1 stoichiometry between Fe^III^ and Fe^II^ (Supplementary information, Fig. S13b and Table S3). This conclusion is further supported by quantitative assays of Fe^III^ and Fe^II^ in the VcMbnBC and RrMbnBC proteins using 1,10-phenanthroline^36^ (Supplementary information, Table S4). Treatment with excess H_2_O_2_ almost abrogated the MbnBC-mediated MbnA modification with negligible impact on the stability of the MbnBC complexes (Supplementary information, Fig. S13c, d), supporting an essential role of Fe^II^ in the catalytic activity of RrMbnBC and VcMbnBC (Supplementary information, Fig. S13e), similar to what has been observed in MtMbnBC.^17^ Thus, three iron ions (Fe1, Fe2, and Fe3) with octahedral coordination were modeled in the densities (Fig. 5b; Supplementary information, Fig. S14a). Detailed analysis of metals in MbnBC proteins is presented in the Supplementary information, Data S1. The divalent ion (Fe1) coordinates to VcMbnA^C18^ in VcMbnABC and to RrMbnA^C25^ in RrMbnABC (Fig. 5b; Supplementary information, Fig. S14a), providing direct evidence for cysteine modifications of MbnA by MbnBC. In VcMbnB, the residues coordinating the iron ions are His^55^, His^91^, Glu^135^, Asp^165^, Asn^168^, His^194^, Asp^209^, and Glu^238^; this is also the case for equivalent residues in RrMbnB (Fig. 5b; Supplementary information, Fig. S14a). Sequence alignment indicated that the Fe-coordinated residues are highly conserved among the MbnB family members (Supplementary information, Fig. S14b), suggesting that the tri-iron cluster is conserved in this enzyme family. Glu^135^ in VcMbnB provides a bidentate carboxylate ligand that bridges Fe1 and Fe2 (Fig. 5b; Supplementary information, Fig. S14c). Solvent molecules complete the coordination of Fe2 and Fe3, with one water molecule coupling the two metal ions (Fig. 5b; Supplementary information, Fig. S14d). The L2 loop of VcMbnB harbors the conserved residues Asp209 and His211, which are critical for Fe3 coordination (Supplementary information, Fig. S15a). However, the equivalent loop in the unpublished structure of HsMbnB is completely disordered (Supplementary information, Fig. S15b), which could explain why a di-iron center, but not a tri-iron center, was observed in the structure of HsMbnB (Supplementary information, Fig. S15c).

Consistent with previous data,^17^ single mutations of the Fe-coordinated residues His^55^, His^91^, Glu^135^, Asp^165^, Asn^168^, His^194^, Asp^209^, and Glu^238^ in VcMbnB or their equivalents in RrMbnBC and MtMbnBC abolished their MbnA modification activity or impaired MbnB protein level (Fig. 5c; Supplementary information, Fig. S16a–c). These results indicate that the integrity of the tri-iron center is critical for the catalytic activity of MbnBC. Circular dichroism (CD) spectroscopic analyses indicated that loss of activity in these MbnBC mutants was not caused by protein misfolding (Supplementary information, Fig. S16b, c). Similar CD analyses were not performed for VcMbnBC because several mutants were expressed at very low levels (Supplementary information, Fig. S16a).

### Catalytic mechanism

**Fig. 6:**
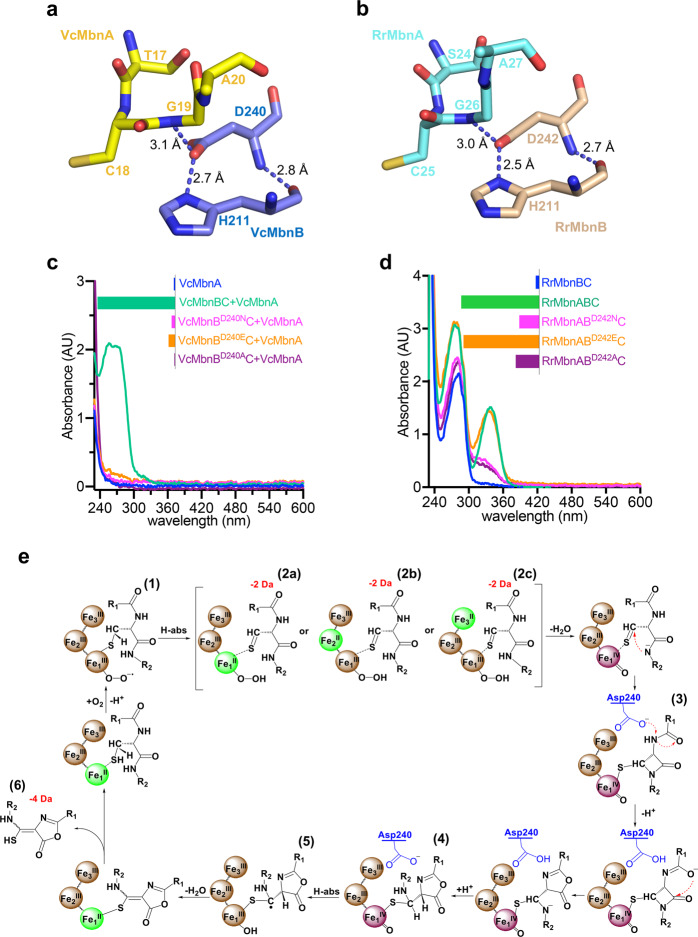
Catalytic mechanism underlying MbnBC-mediated MbnA modification. **a** The structure of VcMbnB^D240^ interacting with VcMbnB^H211^ and VcMbnA^G19^. **b** The structure of RrMbnB^D242^ interacting with RrMbnB^H211^ and RrMbnA^G26^. **c** UV-Vis spectrometric analyses of VcMbnA molecules modified by VcMbnB^D240^C variants (VcMbnB^D240A^C, VcMbnB^D240E^C and VcMbnB^D240N^C). The peak absorbance of VcMbnA is shown at ~270 nm. **d** UV-Vis spectrometric analyses of RrMbnA molecules modified by co-expressed RrMbnB^D242^C variants (RrMbnB^D242A^C, RrMbnB^D242E^C and RrMbnB^D242N^C). The peak absorbance of RrMbnA is shown at ~335 nm. **e** Proposed mechanism for MbnA modification catalyzed by MbnBC. The general base Asp240 of VcMbnB and mass shifts (−2 Da, −4 Da) are indicated. Fe^II^, Fe^III^ and Fe^IV^ irons are colored green, brown, and purple, respectively.

VcMbnB Asp240 and the equivalent in RrMbnB, Asp242, are conserved among MbnBs and are located adjacent to the apex of the curved portion of MbnA (Supplementary information, Fig. S14b). The main chain of VcMbnB Asp240 forms an intramolecular hydrogen bond with VcMbnB^H211^, while the side chain forms a hydrogen bond with the amide nitrogen of VcMbnA^G19^ (Fig. 6a). Similar interactions are also seen in the structure of RrMbnABC (Fig. 6b). Mutation of VcMbnB^D240^ to Ala, Glu or Asn had no detectable effect on VcMbnB’s interaction with VcMbnC but slightly impaired VcMbnA recognition by VcMbnBC (Supplementary information, Fig. S17a, b). However, the mutation abrogated enzymatic activity of the VcMbnBC complex (Fig. 6c). These results collectively suggest that VcMbnB^D240^ likely plays a critical role in catalyzing modification of MbnA. Similar results were observed in equivalent mutants of RrMbnB (Asp242) and MtMbnB (Asp241) (Fig. 6d; Supplementary information, Fig. S17c–g). The exception was RrMbnB^D242E^C, which retained RrMbnA-binding affinity and catalytic activity. Although this suggests that Glu can mimic the function of Asp242 in RrMbnB, the overall results nonetheless point to a conserved role for the Asp residue, acting as a catalytic base for MbnA modification.

The Cys–S–Fe bond of the MbnABC substrate–enzyme complex is reminiscent of the one in isopenicillin *N* synthase (IPNS), a dioxygen-dependent oxidase that is nonheme iron-dependent. IPNS initially catalyzes formation of the β-lactam ring, and then generates the five-membered thiazolidine ring with the assistance of an oxidized iron (IV)-oxo (ferryl) moiety.^37–44^ In addition, previously generated functional data^17^ have suggested that MbnBCs are oxygen-activating nonheme tri-iron-dependent enzymes. Based on these prior findings, we propose a plausible mechanism by which MbnBC catalyzes the production of Mbns (Fig. 6e). First, O_2_ is activated by Fe_1_^II^, which is ligated to the substrate cysteine that is to be modified. This yields the superoxo species of Fe_1_^III^–O_2_^−^ (1), which can further perform H atom abstraction (H-abs) from cysteine C_β_-H to generate the Fe_1_^II^–hydroperoxo complex (2a). Abstraction of the H atom was confirmed via mass spectroscopy, which showed a decreased mass. It was recently proposed that there are likely three irons in MbnBCs,^17^ a hypothesis that is further supported by our data with the clearly observed irons in the MbnABC structure. We identified the valence states of the irons in the MbnB active site via ^57^Fe-Mössbauer, EPR spectroscopy and biochemical assays. Based on these new spectroscopic and structural data, we speculate that the inner-sphere electron transfer from the ligated thioalkyl radical to the Fe_2_^III^ or Fe_3_^III^ site might occur to generate the Fe_2_^II^ species (2b) or Fe_3_^II^ species (2c). This step is followed by O–O cleavage of the Fe_1_–hydroperoxo complex, and the subsequent formation of the β-lactam ring and a Fe^IV^–oxo (ferryl) complex (3). The way in which the third Fe impacts the chemistry of electron transfer requires future investigation. Asp240 subsequently accepts and provides a proton to facilitate formation of a five-member ring intermediate (4) and acts as a general base;^45^ this conclusion is supported by structural analysis and a mutagenesis assay. Finally, the Fe^IV^–oxo-mediated dehydrogenation of the intermediate (5) generates the product Mbn (6).

## Discussion

In the current study, we report crystal structures of MbnABCs from Mbn Groups III and V. The structures reveal a conserved architecture of MbnBC holoenzymes across groups and elucidate the mechanisms underlying substrate recognition and catalysis (Figs. 2, 6e; Supplementary information, Table S1). These data facilitate the identification of MbnA precursors and establish a framework for engineering TIM-barrel enzymes to produce Mbns.

Interaction with MbnC is required for MbnB catalytic activity, indicating that the MbnBC complex acts as a holoenzyme for Mbn generation.^17^ The structures of VcMbnBC and RrMbnBC are nearly identical (Fig. 2), although the former is the least conserved among species (Supplementary information, Fig. S11a, b). These data suggest that MbnBCs serve as a highly conserved machinery for catalytic production of Mbns. Indeed, some MbnA residues responsible for its recognition are conserved throughout Groups I–V (Supplementary information, Fig. S9g). Recognition of the N-terminal MbnA LP by MbnC is remarkably similar to that of RiPP precursor peptides by RREs^30–33^ (Fig. 3b; Supplementary information, Fig. S9), suggesting that recognition of the N-terminal half of the LP does not involve extensive sequence specificity. Recognition of the C-terminal half of LP and CP is mediated by both MbnB and MbnC, which is likely important for specific selection of MbnAs by cognate MbnBCs. Despite MbnBCs having different specificity for MbnAs from different species, the active sites of MbnBCs, including the tri-iron center and the potential catalytic Asp residue are highly conserved (Figs. 5, 6a; Supplementary information, Fig. S14b), suggesting a conserved mechanism for MbnA modifications.

The protein structures presented here show that the cysteine residue to be modified is directly coordinated to the divalent Fe in the tri-iron center (Fig. 5b; Supplementary information, Fig. S14a), although residues surrounding the cysteine are not conserved. RrMbnBC can modify either Cys21 or Cys25 of RrMbnA as indicated by MS data (Fig. 1c; Supplementary information, Fig. S3f), but only Cys25 was found to coordinate to Fe1 in the crystal structure of RrMbnABC (Fig. 3c). This may be a more energetically favorable binding mode for RrMbnA to interact with the catalytic site of RrMbnBC and could therefore be more readily captured by crystallography. In contrast to RrMbnA, only the first cysteine residue Cys21 of MtMbnA was found to be modified although the peptide contains 4 cysteine residues in the CP; the second potential modification on Cys27 that had been reported in a previous study^17^ could not be detected. The precise reason for this discrepancy remains unknown, but the distance between the two potential modification sites in MbnAs (Cys21/25 for RrMbnA, Cys21/27 for MtMbnA) may play a role in determining whether the second modification can occur. Interestingly, the two cysteines in RrMbnA that can be modified alternatively have three residues between them, whereas there are five residues between the two cysteine residues in MtMbnA. After modification of the first cysteine residue, the MbnA precursor peptide would dissociate from the MbnBC complex due to limited space of the active site. These results raise an intriguing possibility that other enzymes may be involved for further modifications of MbnAs that have more than one cysteine residues modified. In agreement with this hypothesis, other enzymes such as MbnN, MbnF and MbnD have been suggested to be important for Mbn biosynthesis.^10,17,23^

Moreover, although the results of acid hydrolysis, the mass shifts and the Cu^2+^-binding properties of modified MbnAs are similar to the characteristics of a single oxazolone-thioamide moiety, the absorption features of the modified VcMbnA by VcMbnBC or RrMbnBC are strikingly different (Figs. 1e, 4c), indicating that the catalytic environment of the active site is critical for product formation. A better understanding of the catalytic environment of these enzymes will rationalize the substrate or enzyme engineering to yield stable and simple Mbns for clinical applications.

## Materials and methods summary

### Peptide information

The VcMbnA peptide (MKNDKKVVVKVKDKEMTCGAFNK) from *Vibrio caribbenthicus* BAA-2122 and the VcMbnA variants LP (leader peptide, VcMbnA1-16) and CP (core peptide, VcMbnA17-23); the MtMbnA peptide (MTVKIAQKKVLPVIGRAAALCGSCYPCSCM) from *Methylosinus trichosporium* OB3b; and the RrMbnA peptide (MKIVIVKKVEIQVAGRTGMRCASSCGAKS) from *Rugamonas rubra* ATCC 43154 were all produced by Zhejiang Ontores Biotechnologies Co., Ltd, China. Peptides used in this study are listed in Supplementary information, Table S5. All peptides were purified to 95% purity except for RrMbnA (< 80% purity), which was too hydrophobic to be purified to the desired purity. All peptides were verified by HPLC and MS. The materials for molecular cloning used in this work were purchased from Vazyme Biotech Co., Ltd (Nanjing, China). All other chemicals were purchased from Sangon Biotech (Shanghai) Co., Ltd (Shanghai, China) unless otherwise noted.

### Gene cloning and protein expression

The *mbnB* and *mbnC* genes originating from *Methylosinus* sp. LW4 (Group I), *Methylosinus* sp. PW1 (Group I), *Mt* OB3b (Group I), *Methylocystis hirsuta* CSC1* (Group II), *Methylocystis rosea* SV79 (Group II), *Methylosinus* sp. LW3 (Group II), *Methylosinus* sp. R-45379 (Group II), *Pseudomonas extremaustralis* DSM17835 (Group III), *Rr* ATCC 43154 (Group III), *Gluconacetobacter* sp. SXCC-1 (Group IV), and *Vc* BAA-2122 (Group V) were all synthesized by GENEWIZ (Hangzhou, China) and codon-optimized for expression in *E. coli*. The pET-Duet-1 vector was used for co-expression of MbnB (non-tagged) and MbnC (N-terminally His-tagged) to generate MbnBC complexes. The *mbnC* genes were subcloned into the CDS-1 region of the vector between the *Bam*HI and *Hin*dIII restriction sites in frame with an N-terminal His_6_ tag. The *mbnB* genes were inserted into CDS-2 using the *Nde*I and *Xho*I restriction sites with a stop codon at the end of *mbnB* sequence; this vector was designated *mbnBC*_pET-Duet-1. Proteins prepared for crystallization, Mössbauer spectroscopy, EPR, activity assays, CD assays, MbnA binding affinity assays, and ICP-MS were all produced from the *mbnBC*_pET-Duet-1 construct. In addition, point mutations in MbnBC were introduced using quick-change PCR or overlap PCR with *mbnBC*_pET-Duet-1 as a template and primers containing the desired base mutations. For the deletion constructs, the truncated sequences were subcloned into the pET-Duet-1 vector as described for the *mbnBC*_pET-Duet-1 construct.

The *mbnAs* from different species were separately constructed by overlap extension PCR using three pairs of primers (codon optimized and synthesized by Tsingke Biotechnology, China) and then cloned into a modified pET28b* vector between the *Xba*I and *Xho*I restriction sites (*mbnA*-pET28b*). The resulting *mbnA*-pET28b* construct contains a C-terminal His_6_ tag. To express RrMbnA with no tag, the stop codon TAA was introduced into the third forward primer of RrMbnA used for PCR amplification, and the sequence was subcloned into the pET28b* vector as described for the *mbnA*-pET28b* construct. All the constructs were verified by Sanger sequencing and are summarized in Supplementary information, Table S6. The primers used in this study are listed in Supplementary information, Table S7.

The *mbnBC*-pET-Duet-1 and *mbnA*-pET28b* plasmids were transformed or co-transformed into *E. coli* BL21 (DE3) cells. LB medium supplemented with 0.25 mM ferrous ammonium sulfate and appropriate antibiotics was used for expression. Cultures were grown at 37 °C and 220 rpm to a final OD_600_ of 0.6, and protein expression was induced by adding isopropyl β-D-thiogalactopyranoside (IPTG) to a final concentration of 200 μM and incubating at 16 °C for 16 h.

### Preparation of ^57^Fe-rich and selenomethionine-derived proteins

The *mbnBC*-pET-Duet-1 plasmids were transformed into *E. coli* BL21 (DE3) cells. For Mössbauer spectroscopy, minimal nutrient M9 medium (the recipe for M9 is provided in Supplementary information, Table S8) supplemented with ^57^Fe (anaerobically dissolved under heat in hydrochloric acid) was used instead of LB medium to enhance the loading of ^57^Fe in the protein and avoid the introduction of additional ^56^Fe. For phase determination of the VcMbnBC crystal structure, selenomethionine (Se-Met)-substituted proteins were expressed in M9 medium supplemented with Se-Met and 0.25 mM ferrous ammonium sulfate. Protein expression was induced by adding IPTG to a final concentration of 200 μM and incubating at 16 °C for 16 h. All the reconstituted MbnBC and MbnABC proteins in this study are summarized in Supplementary information, Table S9.

### Aerobic and anaerobic protein purification

During aerobic purification, cell pellets were harvested by centrifugation at 4000 rpm at 4 °C and resuspended in buffer A (25 mM HEPES, pH 8.0, 200 mM NaCl). The cells were disrupted using a French press (AH-1500, ATS, China). After cell lysis, the lysate was centrifuged at 18,000 rpm for 30 min to remove the unlysed cells and insoluble material. Then the supernatant was applied to a nickel affinity column (Ni-NTA; GE Healthcare, Little Chalfont, UK). Buffer A mixed with a gradient of imidazole (5, 10, and 15 mM) was used to wash the column, and the protein was eluted with buffer A containing 250 mM imidazole. The eluted protein was concentrated using Amicon Ultra-15 concentrators (MilliporeSigma, Darmstadt, Germany) with the appropriate molecular weight cutoffs (3 kDa for co-expressed MbnAs, 10–30 kDa for MbnBC). Anion exchange chromatography (Source Q; GE Healthcare, Sweden) was then used for further purification. The peak fractions were injected into size exclusion chromatography columns (Superdex200 Increase 10/30 GL; GE Healthcare, Sweden) equilibrated with buffer B containing 100 mM NaCl and 25 mM HEPES (pH 8.0). The purity of MbnB and MbnBC was assessed using sodium dodecyl sulfate-polyacrylamide gel electrophoresis (SDS-PAGE), and the proteins were flash-frozen with liquid nitrogen for subsequent crystallization and biochemical analysis. Se-Met-substituted proteins were expressed in M9 medium supplemented with Se-Met and purified just as described for the native proteins. All MbnB, MbnBC, and MbnABC mutations (Supplementary information, Table S6) were produced using the procedure mentioned above.

During the anaerobic purification of MbnBC, all buffers used were oxygen-free and all purification steps were performed in an anaerobic glove box (UN-750S, DELLIX, China) maintained at < 0.1 ppm oxygen. Cell pellets were harvested by centrifugation at 4000 rpm at 4 °C and resuspended in Buffer A (25 mM HEPES, pH 8.0, 200 mM NaCl). The cells were cooled in an ice bath and disrupted by an ultrasonic cell disruptor (Scientz-IID, Ningbo Scientz Biotechnology, China) at 20% output power, with 9-s pulses for a total of 30 min of sonic disruption. The lysate was centrifuged at 18,000 rpm for 30 min to remove the precipitates. Then the supernatant was applied to a nickel affinity column equilibrated with cooled buffer A. Buffer A mixed with a gradient of imidazole (as mentioned above) was used to wash the column, and the protein was eluted with buffer A containing 250 mM imidazole. Protein concentration and buffer replacement were performed using Amicon Ultra-15 concentrators (Millipore Sigma, Darmstadt, Germany) with the appropriate molecular weight cutoffs (3 kDa for co-expressed MbnAs, 10–30 kDa for MbnBC). Then, the concentrated proteins were injected into size exclusion chromatography columns (Superdex200 Increase 10/30 GL; GE Healthcare, Sweden) equilibrated with buffer B containing 100 mM NaCl and 25 mM HEPES (pH 8.0). The purities of MbnB and MbnBC were assessed by SDS-PAGE. Finally, the anaerobically prepared proteins were stored in a threaded tube with a rubber ring to avoid contamination by oxygen from the air and flash frozen for further assays.

### Crystallization

The purified VcMbnBC and RrMbnABC (RrMbnBC co-expressed with RrMbnA) proteins were concentrated to 10 mg/mL and 15 mg/mL for crystallization, respectively. A crystallization screening assay was carried out using five classic screening kits, namely Index, Crystal, SaltRX, WizardI/II/III/IV, and PEG/ION. VcMbnBC incubated with synthesized VcMbnA at 4 °C for 2–3 h (molar ratio = 1:8) was used for the screening assays. Crystals of VcMbnABC grew to suitable sizes for X-ray diffraction within two weeks. To determine the phases of these complex structures, we purified Se-Met-substituted VcMbnBC, and the protein was incubated with synthesized VcMbnA for crystallization, The Se-derived VcMbnABC complex was crystallized under the same conditions as the native protein. Crystals of VcMbnABC were obtained with 2% Tacsimate (pH 7.0), 0.1 M HEPES (pH 7.5) and 20% PEG 3350, then flash-frozen in liquid nitrogen with an additional 12% glycerol added as a cryoprotectant during X-ray diffraction. Crystals of RrMbnABC were obtained after four days of incubation in a screening buffer containing 10% PEG 20000 and 100 mM MES (pH 6.5).

### Data collection and structure determination

X-ray diffraction datasets of Se-derived VcMbnBC protein in complex with VcMbnA and the RrMbnABC complex were collected at the BL18U1 beamline end station of the Shanghai Synchrotron Radiation Facility at a wavelength of 0.9789 Å. All diffraction pictures collected were processed using the XDS data processing package or the HKL3000 package. The phase of the VcMbnABC complex was determined using the single-wavelength anomalous dispersion (SAD) method using the Se-Met-labeled MbnBC protein. The Se-Met sites were searched for and identified using the HySS module in the Phenix package, and density modification was performed using Resolve. An initial model was automatically built by Resolve in the phenix autobuild tool and then refined using phenix refinement. The final model was determined after being repeatedly manually built in COOT^46^ and refined in Phenix.^47^ The RrMbnABC structure was determined by molecular replacement (Molrep, Phenix) using the VcMbnBC complex structure as a searching model, manually built in COOT, and refined by Phenix. The statistics for X-ray diffraction data collection and refinement are listed in Supplementary information, Table S1.

### Limited proteolysis assay

The limited proteolysis assay allows the characterization of the increase in stability of wild-type proteins upon binding to their substrates. The VcMbnBC and RrMbnBC proteins used in this assay were all adjusted to an equal molar concentration of about 20 µM. The RrMbnBC or VcMbnBC complex, by itself or after incubation with the corresponding MbnA peptide for 1 h at 4 °C, was digested by trypsin. After 30 min of incubation on ice, the proteolyzed products were analyzed by SDS-PAGE and visualized by Coomassie blue staining.

### Fe content determination

#### ICP-MS

ICP-MS measurements were carried out on the 7900 ICP-MS Agilent system (Agilent Technologies Inc., CA, USA). The MtMbnBC, RrMbnBC, RrMbnABC, and VcMbnBC proteins were mixed with 8 mL nitric acid for 15 min to predigest them. The supernatant was then loaded into the ICP-MS instrument for analysis of the Fe, Ni, Co, and Cu contents. Data are shown in Supplementary information, Table S2.

#### EPR

For EPR analysis, ~120 μL aliquots of anaerobically and aerobically prepared VcMbnBC (400 μM), RrMbnBC (400 μM), and MtMbnBC (300 μM) were transferred into EPR tubes in an anaerobic glove box maintained at < 0.1 ppm oxygen, and the nozzles of the EPR tubes were all sealed with plasticine to avoid oxygen contamination. The samples were then frozen in liquid nitrogen for analysis. The continuous-wave EPR (CW-EPR) spectrum was acquired on a Bruker Elexsys E580 spectrometer with a super-high sensitivity probe head (ω = 9.36 GHz). Data were acquired under a helium flow cryostat at 5 K, and the temperature was controlled by an ESR900 Oxford instrument. The experimental parameters were set as follows: microwave frequency 9.376 GHz (5 K), microwave power 2.37 mW, 4 G modulation amplitude, and 30 ms time constant and conversion time.

#### Mössbauer spectroscopy

For Mössbauer analysis, ~45 mg of aerobically purified, ^57^Fe-enriched, and lyophilized RrMbnBC and VcMbnBC were transferred into a Mössbauer cup and frozen by placing the cup on an aluminum block precooled with liquid nitrogen. All the sample preparation steps were conducted in an anaerobic glove box maintained at < 0.1 ppm oxygen. The ^57^Fe Mössbauer spectra were measured using a conventional Mössbauer spectrometer (Topologic Systems, Kanagawa, Japan) in transmission mode with a 57Co/Rh γ-ray source. The samples were tightly sealed with silicon grease in an acrylic holder, and the spectra were calibrated using α-Fe foil as a reference at room temperature. Simulation of the Mössbauer spectra was carried out using the WMOSS spectral analysis software (www.wmoss.org; Seeco Research, Edina, MN). Mössbauer simulation parameters of the tri-iron cluster of VcMbnBC and RrMbnBC are shown in Supplementary information, Table S3.

#### 1,10-phenanthroline assay

The relative concentrations of Fe^II^ and total Fe were measured using the standard 1,10-phenanthroline method^36^ in which 1,10-phenanthroline forms a stable complex with Fe^II^ that absorbs light at 510 nm. For sample preparation, the proteins (aerobically or anaerobically purified MtMbnBC, RrMbnBC, RrMbnABC, and VcMbnBC) were adjusted to 1 mg/mL, then digested by trypsin at a mass ratio of 1:1 for 2 h at room temperature; proteolysis of the proteins allows exposure of the iron to the solution for detection. For Fe^II^ determination, 20 μL of 1 M sodium ethanoate solution and 150 μL of 5 mM 1,10-phenanthroline solution were mixed with 250 μL of protein–trypsin mixture sample and water (up to a final volume of 1 mL), then incubated for 10 min until detection. For total Fe determination, an additional 10 μL of 1.5 M hydroxylamine hydrochloride was introduced to reduce the Fe^III^ to Fe^II^, and the remaining steps for iron content determination were the same as those for Fe^II^. Finally, the absorbance at 510 nm was acquired by a UV-Vis and Vis instrument (Ultrospec 2100 pro, Biochrom, UK) using a quartz cuvette. For generating the standard curve, a standard solution of 0.100 g/dm^3^ Fe^II^ (Fe (NH_4_)_2_(SO_4_)_2_·6H_2_O) was prepared and the iron contents of 0, 10, 20, 30, 40, and 50 μL of the standard solution were determined following the total Fe determination method. The processed data are summarized in Supplementary information, Table S4.

### Pull-down assay for the MbnBC and MbnABC complexes

The *mbnBC*-pET-Duet-1 construct, which results in the co-expression of His_6_-MbnC and MbnB without a tag, and *mbnA*-pET-28b* were used for the pull-down assay. The MbnBC complex was applied to a nickel affinity column; the MbnB protein could be pulled down by His_6_-MbnC. The constructs for the tri-iron binding site mutations of MbnB and mutations of the MbnB and MbnC interface were designed using a strategy similar to that used for the wild-type *mbnBC*-pET-Duet-1 construct. For reconstitution of the VcMbnABC complex, the VcMbnBC complex was incubated with synthetic VcMbnA in vitro, and then purified by a nickel column. For reconstitution of RrMbnABC and other MbnABC complexes, the *mbnBC*-pET-Duet-1 and *mbnA*-pET28b* constructs were co-transformed into *E. coli* BL21 (DE3) for co-expression, then protein purification was performed as described above.

### Far-ultraviolet CD

Far-ultraviolet CD spectra for the wild-type RrMbnBC and MtMbnBC proteins and the RrMbnBC and MtMbnBC mutants were captured between 190 and 260 nm using a Chirascan plus spectropolarimeter (Applied Photophysics, England) at 15 °C. Proteins were thawed in solvent (10 mM Tris-HCl, pH 8.0). The concentrations of proteins were all maintained at 10 µM.

### UV-Vis spectroscopy for MbnA activity assays (standard and stopped-flow absorption spectroscopy)

Standard UV-Vis spectra detection was performed on a UV-Vis and Vis instrument (Ultrospec 2100 pro, Biochrom, UK) at room temperature. MtMbnBC and RrMbnBC proteins were adjusted to 5 mg/mL (nearly 100 μM), and VcMbnBC was adjusted to a concentration of 1 mg/mL (nearly 20 μM). The corresponding substrates, VcMbnA, RrMbnA, MtMbnA, and their variants (Supplementary information, Table S5), were dissolved in distilled water and diluted to a working concentration of 5 mM with buffer B. The reaction was initiated when 100 μL MbnBC proteins and 5 μL MbnA peptides were applied to the reaction system, and the reaction was diluted up to 1 mL with buffer B. The mixture was then added into a quartz cuvette and recorded at 20-s intervals for a total of 50 reads at room temperature. For assays using anaerobically prepared MbnBC proteins, the measurements were immediately recorded by adding the anaerobically prepared MbnBC into the reaction system, with peptides pre-mixed in the quartz cuvette. The buffer B used in the assay was O_2_-saturated at room temperature.

For the stopped-flow UV-Vis spectroscopic analysis, 10 μM MtMbnBC or RrMbnBC or 2 μM VcMbnBC was incubated with 250 μM MbnA peptides at room temperature for 1 h, followed by detection on a UV-Vis and Vis instrument (Nanodrop One, Thermo Scientific, MA). Data were processed by Graphpad Prism version 8.3.

### MbnBC activity assay followed by H_2_O_2_ treatment

H_2_O_2_ was first incubated with proteins (VcMbnBC, MtMbnBC, and RrMbnBC) for 5 min and the proteins were further purified by size exclusion chromatography. The H_2_O_2_-treated MbnBC complexes were then used for the activity assay.

### ITC measurements of MbnA binding affinity

MbnBC and the mutant proteins were all purified using the two-step standard method described above. The purified proteins were adjusted to 1 mg/mL and stored in 25 mM HEPES (pH 8.0) and 100 mM NaCl for ITC measurement. The MbnAs were dissolved and diluted to a working concentration of 2 mM in distilled water. ITC experiments were performed on a Nano ITC (Nano ITC-Low Volume; TA instrument, USA). Settings for the MbnA binding assay were optimized as follows: the titration assay was performed at 16 °C by injecting 2.5 μL MbnA peptide into a full cell containing 190 μL MbnBC or mutant MbnBC proteins every 120 s for a total of 20 injections, and the stirrer syringe stirring speed was set at 250 rpm.

For measurement of Cu^2+^ binding affinity with modified MbnAs, all modified MbnAs were extracted from the enzyme mixtures by size exclusion chromatography except for modified RrMbnA, which was isolated from the co-expressed RrMbnABC complex (the filtrate of 30-kDa centrifugal ultrafiltration tubes). The BCA kit purchased from Suo Laibao Biotechnology Co., Ltd (Beijing, China) was used for determining the concentrations of MbnAs; ~0.2 mM Cu^2+^ was titrated into 0.01 mM modified MbnAs for the ITC measurement, and the synthesized unmodified MbnAs were used as controls in the assay. The raw titration datasets were processed using the one-site binding mode of the NanoAnalyze Data Analysis software version 3.8.0.

### MS analysis

To prepare the samples for MS analysis, the wild-type proteins RrMbnBC and VcMbnBC were separately incubated with synthetic VcMbnA, respectively, to generate the modified VcMbnA products accordingly. The product was applied to an analytic size exclusion chromatography column (Superdex200 Increase 10/300 GL; GE Healthcare, Sweden) to separate the modified MbnAs from the reaction system. Specifically, modified RrMbnA was isolated from the co-expressed RrMbnABC complex as described for ITC sample preparation (the filtrate of 30-kDa centrifugal ultrafiltration tubes). Then, the fractions containing the modified MbnAs were collected and freeze-dried. Lyophilized powder was dissolved in 0.1% formic acid H_2_O and applied to a C18 ZipTip micro-chromatography desalination column (Millipore, Massachusetts, USA) to remove the existing salts, following the manufacturer’s instructions. The MS assay was performed on an Orbitrap Fusion Lumos mass spectrometer (ThermoFisher Scientific, San Jose, CA, USA) equipped with a nano-ESI ion source providing high-energy collision dissociation (HCD), collision-induced dissociation (CID), and electron-transfer dissociation (ETD) fragmentation analysis. The peptides were separated by an analytical capillary column (100 μm × 15 cm) packed with 3 μm spherical C18 reversed phase material (Dr. Masch GmbH, Germany). An EASY-nLC 1200 (ThermoFisher Scientific, USA) was used to generate the following HPLC gradient: 0%–65% B over 50 min, 50%–80% B over 5 min, and 80% B for 2 min (A = 0.1% formic acid, 99.9% H_2_O; B = 80% acetonitrile, 20% H_2_O, 0.1% formic acid). The mass spectrometer was operated in data-dependent mode with one MS scan followed by HCD MS/MS scans and ETHCD (electron-transfer/higher-energy collision dissociation) for each cycle in 3 s at top speed. The original data were analyzed using Thermo Xcalibur QualBrowser and the Thermo Proteome Discoverer 2.5.0.400.

### Hybrid cluster-continuum methodology

The transformation of oxazolone/thioamide product in water solution was investigated with hybrid cluster-continuum (HCC) model calculations.^48^ This model has previously been used to study chemical reactions in aqueous solutions, such as hydration and hydrolysis reactions,^49–52^ yielding thermodynamic properties and mechanistic results comparable to those obtained from more advanced ab initio molecular dynamics (MD) simulations.^53^ All of the HCC calculations were performed with the Gaussian 16 software package.^54^ The geometries of all of the transition states, reactants, and intermediates involved in the reaction were fully optimized using a hydrated cluster in conjunction with the Solvation Model Based on Density (SMD) continuum solvation model^55^ at the level of B3LYP/6-31 G(d) theory. Harmonic frequency calculations were performed using the equilibrium geometries to confirm the existence of first-order saddle points and local minima on the potential energy surfaces and to estimate the zero-point energies, as well as the thermal and entropic corrections. The connections between the stable structures and the transition states were ascertained by analyzing the corresponding imaginary frequency modes, as well as by limited intrinsic reaction coordinate (IRC) calculations. The relative energies of the B3LYP/6-31 G(d)-optimized structures were further refined by single-point calculations at the B3LYP/6-311 + +G(d,p) level.

## Supplementary information

Supplementary Figure S1

Supplementary Figure S2

Supplementary Figure S3

Supplementary Figure S4

Supplementary Figure S5

Supplementary Figure S6

Supplementary Figure S7

Supplementary Figure S8

Supplementary Figure S9

Supplementary Figure S10

Supplementary Figure S11

Supplementary Figure S12

Supplementary Figure S13

Supplementary Figure S14

Supplementary Figure S15

Supplementary Figure S16

Supplementary Figure S17

Supplementary Table S1

Supplementary Table S2

Supplementary Table S3

Supplementary Table S4

Supplementary Table S5

Supplementary Table S6

Supplementary Table S7

Supplementary Table S8

Supplementary Table S9

## Data Availability

Coordinates and structure factors are available in the RCSB Protein Data Bank with accession numbers 7DZ9 and 7FC0 for VcMbnABC and RrMbnABC, respectively. All other data are available in the main text or Supplementary information.
