# Supplementary material for: Crystal structure and catalytic mechanism of the MbnBC holoenzyme required for methanobactin biosynthesis
Source: Cell Res. 2022 Feb 2;32(3):302–14. doi: 10.1038/s41422-022-00620-2 (PMC8888699; doi:10.1038/s41422-022-00620-2)
Supplement: Supplementary file 1 — Supplementary Figure S1 [file 41422_2022_620_MOESM1_ESM.pdf]

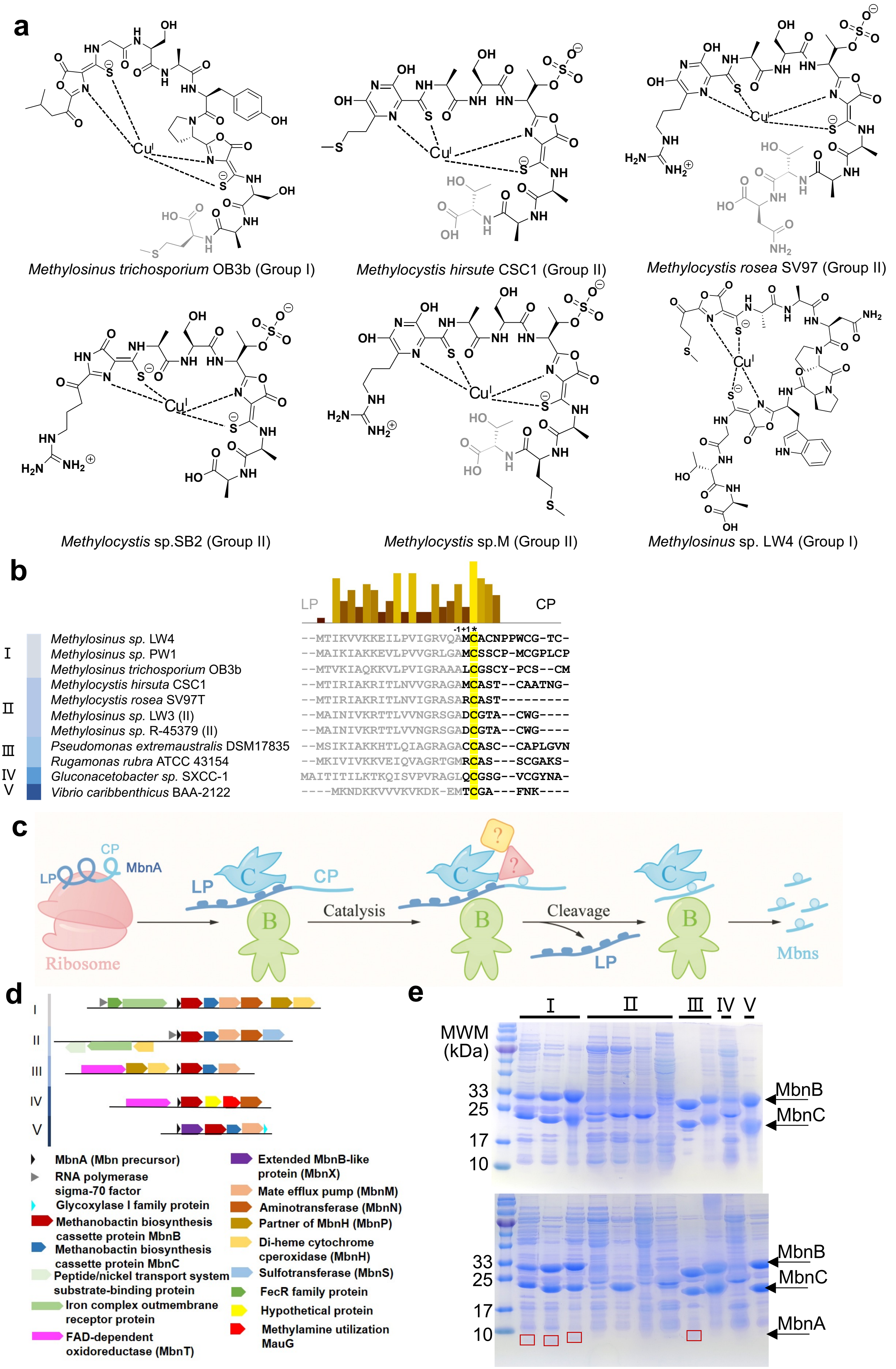

a
Methylosinus trichosporium OB3b (Group I)
Methylocystis hirsute CSC1 (Group II)
Methylocystis rosea SV97 (Group II)
Methylocystis sp.SB2 (Group II)
Methylocystis sp.M (Group II)
Methylosinus sp. LW4 (Group I)
b
LP
CP
-1+1*
I
Methylosinus sp. LW4 --MTIKVVKKEILPVIGRVQAMCACNPPWCG-TC-
Methylosinus sp. PW1 --MAIKIAKKEVLPVVGRLGAMCSSCP-MCGPLCP
Methylosinus trichosporium OB3b --MTVKIAQKKVLPVIGRAAALCGSCY-PCS--CM
II
Methylocystis hirsuta CSC1 --MTIRIAKRITLNVVGRAGAMCAST--CAATNG-
Methylocystis rosea SV97T --MTIRIAKRITLNVIGRASARCAST---------
Methylosinus sp. LW3 (II) --MAINIVKRTTLVVNGRSGADCGTA--CWG----
Methylosinus sp. R-45379 (II) --MAINIVKRTTLVVNGRSGADCGTA--CWG----
III
Pseudomonas extremaustralis DSM17835 --MSIKIAKKHTLQIAGRAGACCASC--CAPLGVN
Rugamonas rubra ATCC 43154 --MKIVIVKKVEIQVAGRTGMRCAS---SCGAKS-
IV
Gluconacetobacter sp. SXCC-1 MAITITILKTKQISVPVRAGLQCGSG--VCGYNA-
V
Vibrio caribbenthicus BAA-2122 ----MKNDKKVVVKVKDK-EMTCGA---FNK----
c
CP
MbnA
LP
Ribosome
LP
C
CP
B
Catalysis
?
?
Cleavage
LP
Mbns
d
I
II
III
IV
V
MbnA (Mbn precursor)
RNA polymerase sigma-70 factor
Glycoxylase I family protein
Methanobactin biosynthesis cassette protein MbnB
Methanobactin biosynthesis cassette protein MbnC
Peptide/nickel transport system substrate-binding protein
Iron complex outmembrane receptor protein
FAD-dependent oxidoreductase (MbnT)
Extended MbnB-like protein (MbnX)
Mate efflux pump (MbnM)
Aminotransferase (MbnN)
Partner of MbnH (MbnP)
Di-heme cytochrome cperoxidase (MbnH)
Sulfotransferase (MbnS)
FecR family protein
Hypothetical protein
Methylamine utilization MauG
e
I
II
III
IV
V
MWM (kDa)
33
25
17
10
MbnB
MbnC
33
25
17
10
MbnB
MbnC
MbnA

Fig. S1. Mbns and mbn operons.

(a) Structures of copper-chelated Mbns characterized to date. **(b)** ClustalW alignment of MbnA sequences from five groups. Leader peptide (LP) and core peptide (CP) sequences are colored in gray and in black, respectively. The alignment is visualized in Jalview, and both LP and CP exhibit well-conserved motifs except for VcMbnA. The potentially modified residue is highlighted in yellow. **(c)** Cartoon representation of Mbn biosynthesis. The uncharacterized enzymes involved in the modification are labeled as boxed question marks. **(d)** Schematic of typical Mbn operons from different groups. Groups are designated via phylogenetic analysis of MbnA, MbnB, and MbnC. **(e)** Reconstitution of MbnBC (upper) and MbnABC (lower) complexes from a range of species containing Mbn operons. SDS-PAGE gel showing, from left to right, constructs from (1) *Methylosinus* sp. LW4 (Group I); (2) *Methylosinus* sp. PW1(Group I); (3) *Methylosinus trichosporium* OB3b (Group I); (4) *Mc.hirsuta* CSC1 (Group IIa); (5) *Methylocystis rosea* SV97T (Group IIa); (6) *Methylosinus* sp. LW3 (II) (Group IIb); (7) *Methylosinus* sp. R-45379 (II) (Group IIb); (8) *Pseudomonas extremaustralis* DSM17835 (Group III); (9) *Rugamonas rubra* ATCC 43154 (Group III); (10) *Gluconacetobacter* sp. SXCC-1 (Group IV) and (11) *Vibrio caribbenthicus* BAA-2122 (Group V), consistent with Supplementary information Fig. S1b. The MbnA, MbnB and MbnC positions are indicated with black arrows. The MbnA, MbnB and MbnC positions are indicated with black arrows.
