# Supplementary material for: Crystal structure and catalytic mechanism of the MbnBC holoenzyme required for methanobactin biosynthesis
Source: Cell Res. 2022 Feb 2;32(3):302–14. doi: 10.1038/s41422-022-00620-2 (PMC8888699; doi:10.1038/s41422-022-00620-2)
Supplement: Supplementary file 2 — Supplementary Figure S2 [file 41422_2022_620_MOESM2_ESM.pdf]

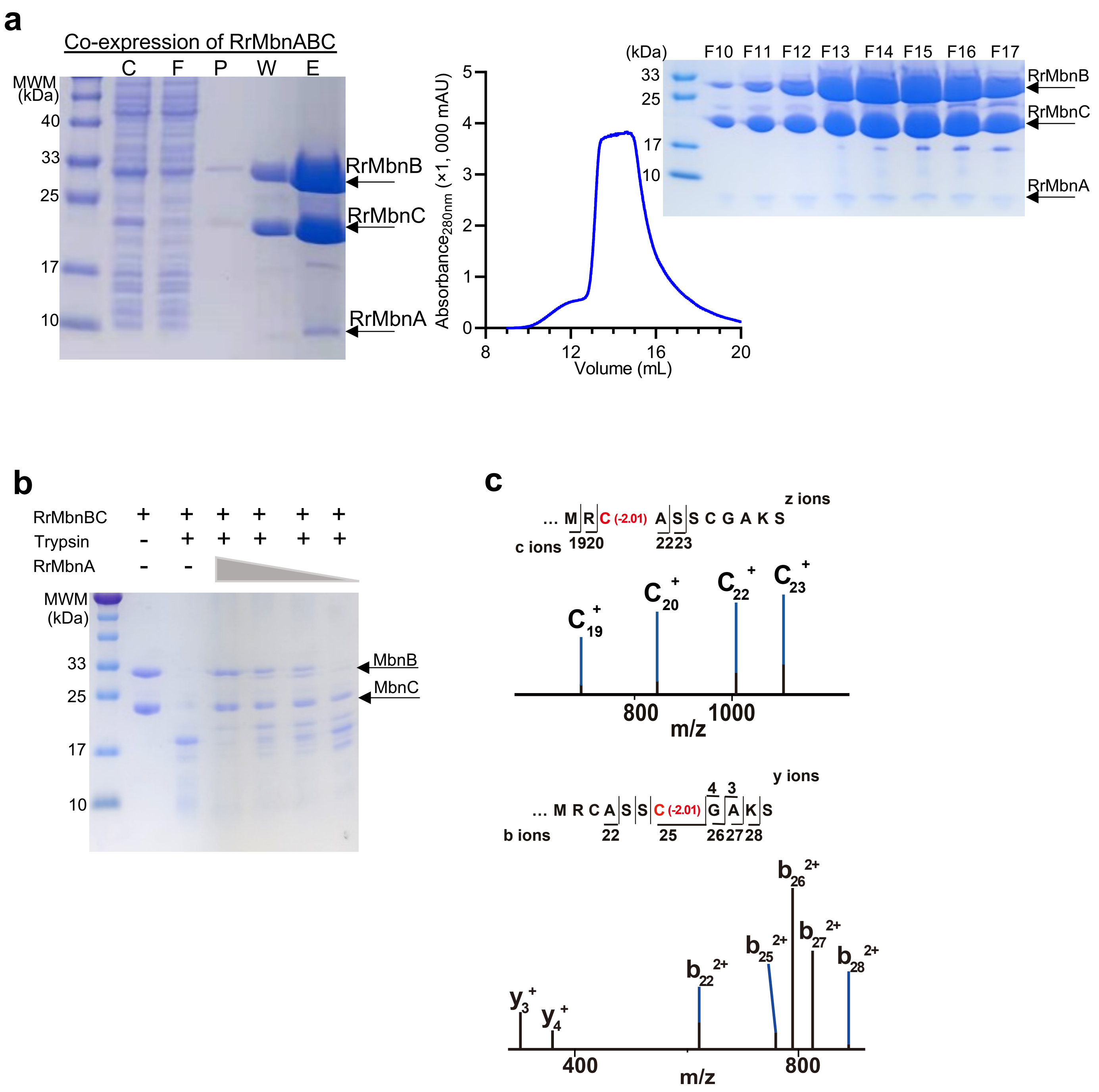

Fig. S2. Reconstitution of the RrMbnABC complex.

(a) SDS-PAGE gel showing co-expressed RrMbnABC complex and purified fractions of gel filtration chromatography by Coomassie staining, indicating that RrMbnA, RrMbnB and RrMbnC form a complex. C, crude; F, flow through; P, pellet; W, wash; E, eluent; F10–17, 10–17 mL fractions of gel filtration chromatography. **(b)** Analysis of trypsin digestion of RrMbnBC in the presence or absence of RrMbnA with Coomassie staining SDS-PAGE gel. **(c)** Analysis of the modified RrMbnA by ESI-MS/MS. The mass shifts at Cys21 and Cys25 of RrMbnA are highlighted in red (-2.01 Da).
