# Supplementary material for: Crystal structure and catalytic mechanism of the MbnBC holoenzyme required for methanobactin biosynthesis
Source: Cell Res. 2022 Feb 2;32(3):302–14. doi: 10.1038/s41422-022-00620-2 (PMC8888699; doi:10.1038/s41422-022-00620-2)
Supplement: Supplementary file 3 — Supplementary Figure S3 [file 41422_2022_620_MOESM3_ESM.pdf]

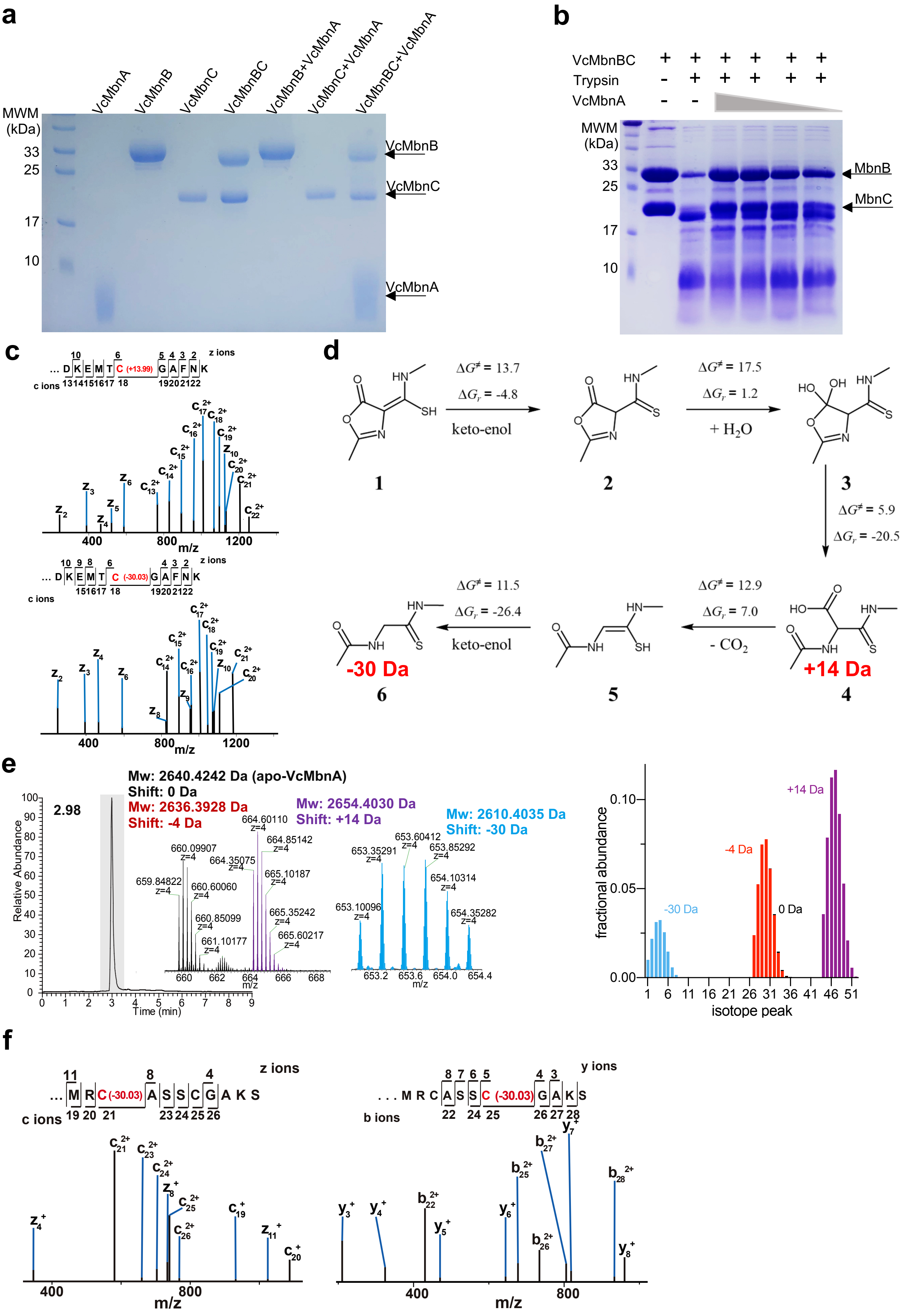
a
VcMbnA
VcMbnB
VcMbnC
VcMbnBC
VcMbnB+VcMbnA
VcMbnC+VcMbnA
VcMbnBC+VcMbnA
MWW (kDa)
33
25
17
10
VcMbnB
VcMbnC
VcMbnA
b
VcMbnBC
Trypsin
VcMbnA
MWW (kDa)
33
25
17
10
MbnB
MbnC
c
z ions
c ions
... D K E M T C (+13.99) G A F N K
... D K E M T C (-30.03) G A F N K
m/z
400
800
1200
d
ΔG≠ = 13.7
ΔGr = -4.8
keto-enol
ΔG≠ = 17.5
ΔGr = 1.2
+ H2O
ΔG≠ = 5.9
ΔGr = -20.5
ΔG≠ = 12.9
ΔGr = 7.0
- CO2
ΔG≠ = 11.5
ΔGr = -26.4
keto-enol
1
2
3
4
5
6
+14 Da
-30 Da
e
Mw: 2640.4242 Da (apo-VcMbnA)
Shift: 0 Da
Mw: 2636.3928 Da
Shift: -4 Da
Mw: 2654.4030 Da
Shift: +14 Da
Mw: 2610.4035 Da
Shift: -30 Da
2.98
Relative Abundance
Time (min)
m/z
fractional abundance
isotope peak
-30 Da
-4 Da
0 Da
+14 Da
f
z ions
c ions
...M R C (-30.03) A S S C G A K S
y ions
b ions
. . . M R C A S S C (-30.03) G A K S
m/z
400
800

Fig. S3. Reconstitution of the VcMbnABC complex.

(a) Pull down assay of the VcMbnABC complex (see Methods). **(b)** Analysis of trypsin digestion of VcMbnBC in the presence or absence of VcMbnA by Coomassie staining SDS-PAGE gel. **(c)** Analysis of the modified VcMbnA by ESI-MS/MS. The mass shifts at Cys18 of VcMbnA are denoted and highlighted in red (-30 Da and +14 Da). **(d)** Proposed scheme for acid hydrolysis of the modified MbnA. The mass shifts during the reaction are indicated and highlighted in red. $\Delta G^{\neq}$ and ΔG_r represent the calculated activation Gibbs energy and Gibbs free energy (in $kcal{\cdot}mol^{-1}$), respectively. Keto-enol denotes the keto-enol tautomerization or the reverse process. In brief, 1 is first converted to 2 through keto-enol tautomerizations. Subsequently, 2 is hydrolyzed to form 4 with intermediate 3. Decarboxylation of 4 forms 5. Finally, 5 is converted to 6 through keto-enol tautomerizations. The mass shifts +14 Da and -30 Da are shown in red. **(e)** Total ion chromatogram and mass spectra (inset) of VcMbnA modified by VcMbnBC. The apo–VcMbnA molecular weights is 2640.4242 Da, the molecular weights of modified VcMbnA are 2636.3928 Da, 2610.4035 Da and 2654.4030 Da, corresponding to mass shifts of -4 Da, -30 Da and +14 Da, respectively. The relative abundance of the modified VcMbnA components is represented in the histogram on the right; the unmodified state is 1%, the -4 Da state is 34%, the -30 Da state is 14%, and the +14 Da state is 51%. **(f)** Analysis of RrMbnA modification by ESI-MS/MS, with mass shifts at Cys21 and Cys25 noted and highlighted in red (-30 Da).
