# Supplementary material for: Crystal structure and catalytic mechanism of the MbnBC holoenzyme required for methanobactin biosynthesis
Source: Cell Res. 2022 Feb 2;32(3):302–14. doi: 10.1038/s41422-022-00620-2 (PMC8888699; doi:10.1038/s41422-022-00620-2)
Supplement: Supplementary file 4 — Supplementary Figure S4 [file 41422_2022_620_MOESM4_ESM.pdf]

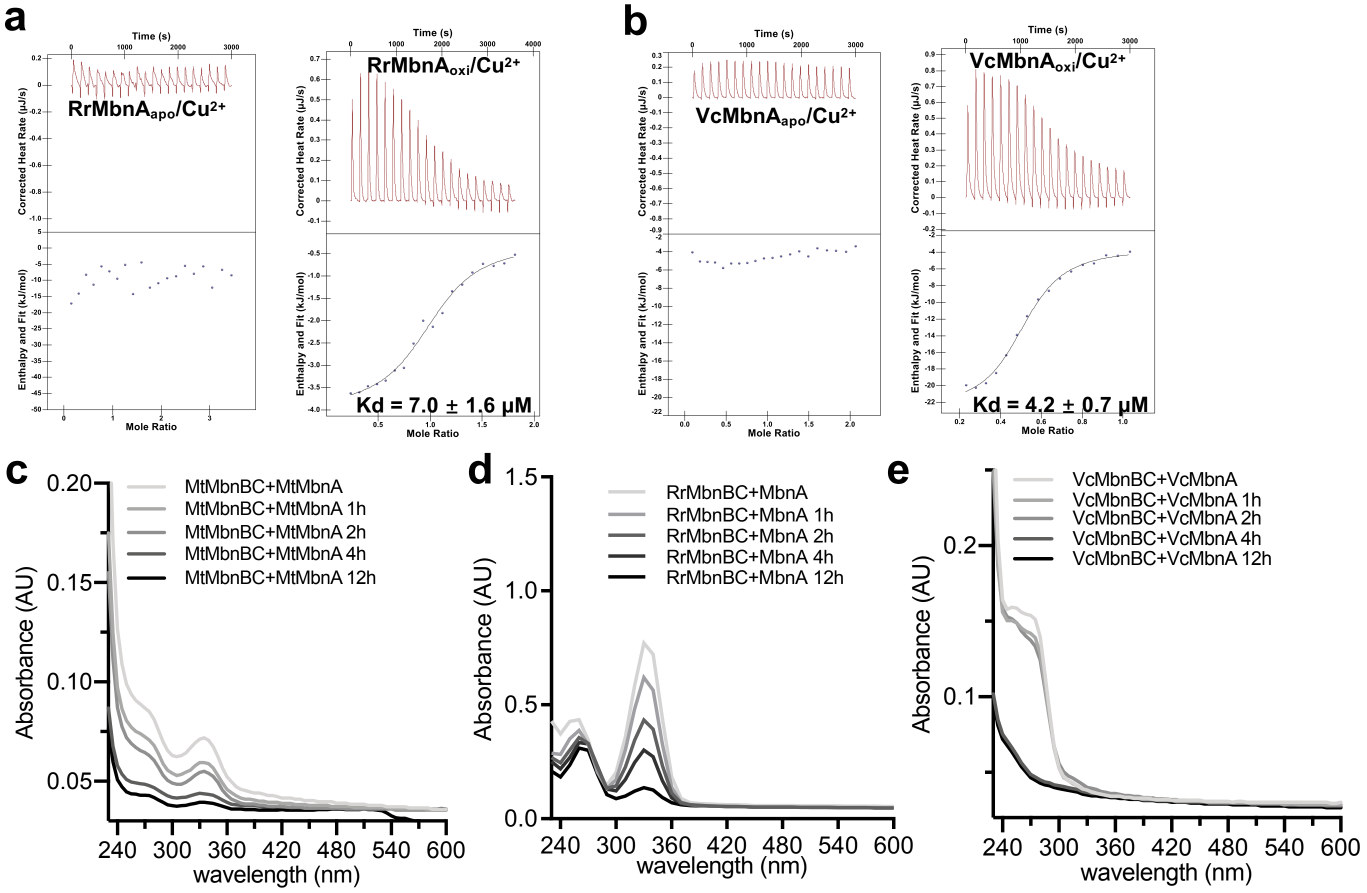


**Fig. S4. $Cu^{2+}$ binding affinity and acid hydrolysis analysis of modified MbnAs**.

**(a)** ITC measurements of unmodified RrMbnA (left) and modified RrMbnA with $Cu^{2+}$ (right) binding affinity. The upper panel shows the original titration traces. **(b)** ITC measurements of unmodified VcMbnA (left) and modified VcMbnA with $Cu^{2+}$ (right) binding affinity. The top panel shows the original titration traces. **(c-e)** Acid-catalyzed hydrolysis of modified MtMbnA (c), RrMbnA (d), and VcMbnA (e). UV-Vis spectra over the first 12 h after the addition of hydrochloric acid to a concentration of 10 mM. Preparation of modified MbnA variants is described in the Methods.
