# Supplementary material for: Crystal structure and catalytic mechanism of the MbnBC holoenzyme required for methanobactin biosynthesis
Source: Cell Res. 2022 Feb 2;32(3):302–14. doi: 10.1038/s41422-022-00620-2 (PMC8888699; doi:10.1038/s41422-022-00620-2)
Supplement: Supplementary file 5 — Supplementary Figure S5 [file 41422_2022_620_MOESM5_ESM.pdf]

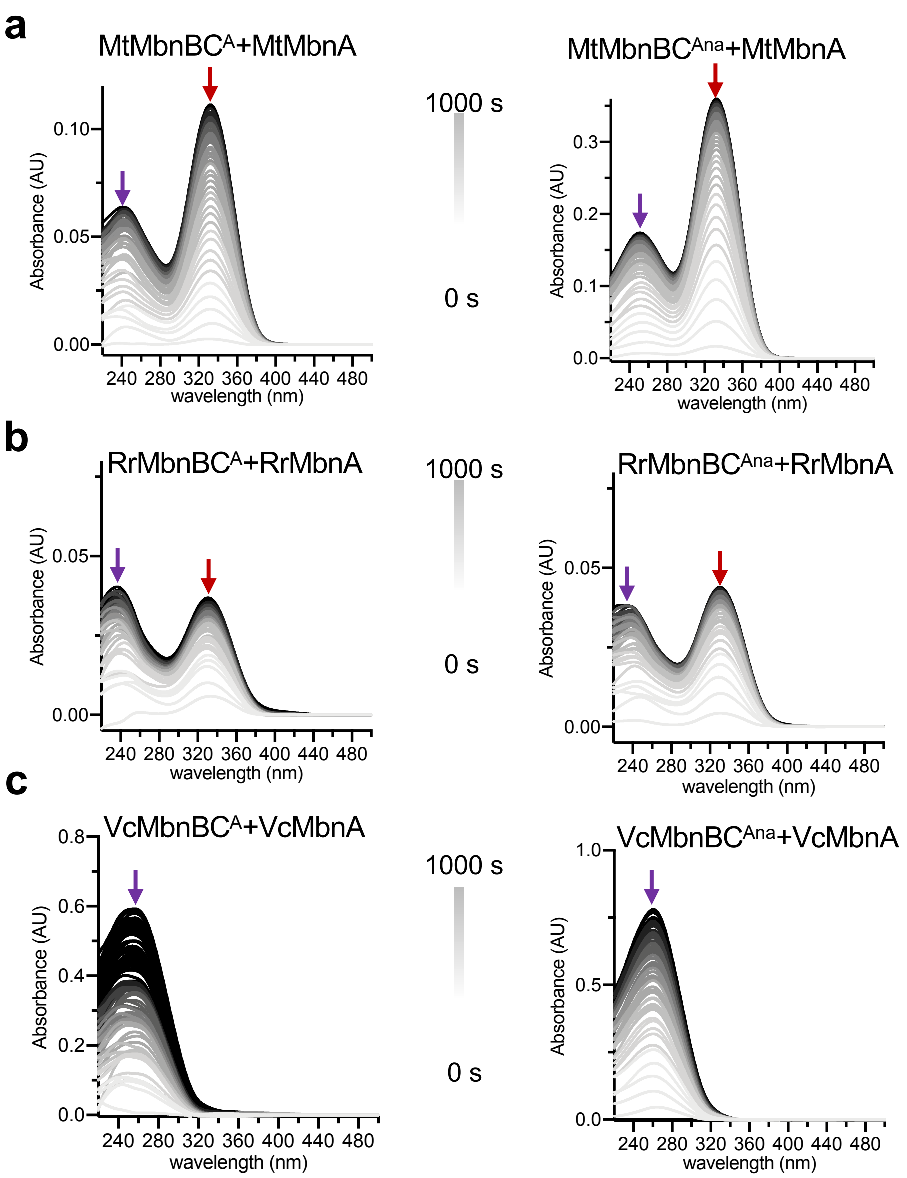

Fig. S5. Activities of aerobically and anaerobically prepared MbnBCs.

(a-c) Standard UV-Vis spectra detection for the enzymatic activity of aerobically (represented as superscript "A") or anaerobically (represented as superscript "Ana") purified MtMbnBC (a), RrMbnBC (b) and VcMbnBC (c) enzymes. Detection was performed with the addition of the substrate MbnA over 1000 seconds. The feature absorbances at 270 nm and 335 nm are indicated by violet and red arrows, respectively.
