# Supplementary material for: Crystal structure and catalytic mechanism of the MbnBC holoenzyme required for methanobactin biosynthesis
Source: Cell Res. 2022 Feb 2;32(3):302–14. doi: 10.1038/s41422-022-00620-2 (PMC8888699; doi:10.1038/s41422-022-00620-2)
Supplement: Supplementary file 6 — Supplementary Figure S6 [file 41422_2022_620_MOESM6_ESM.pdf]

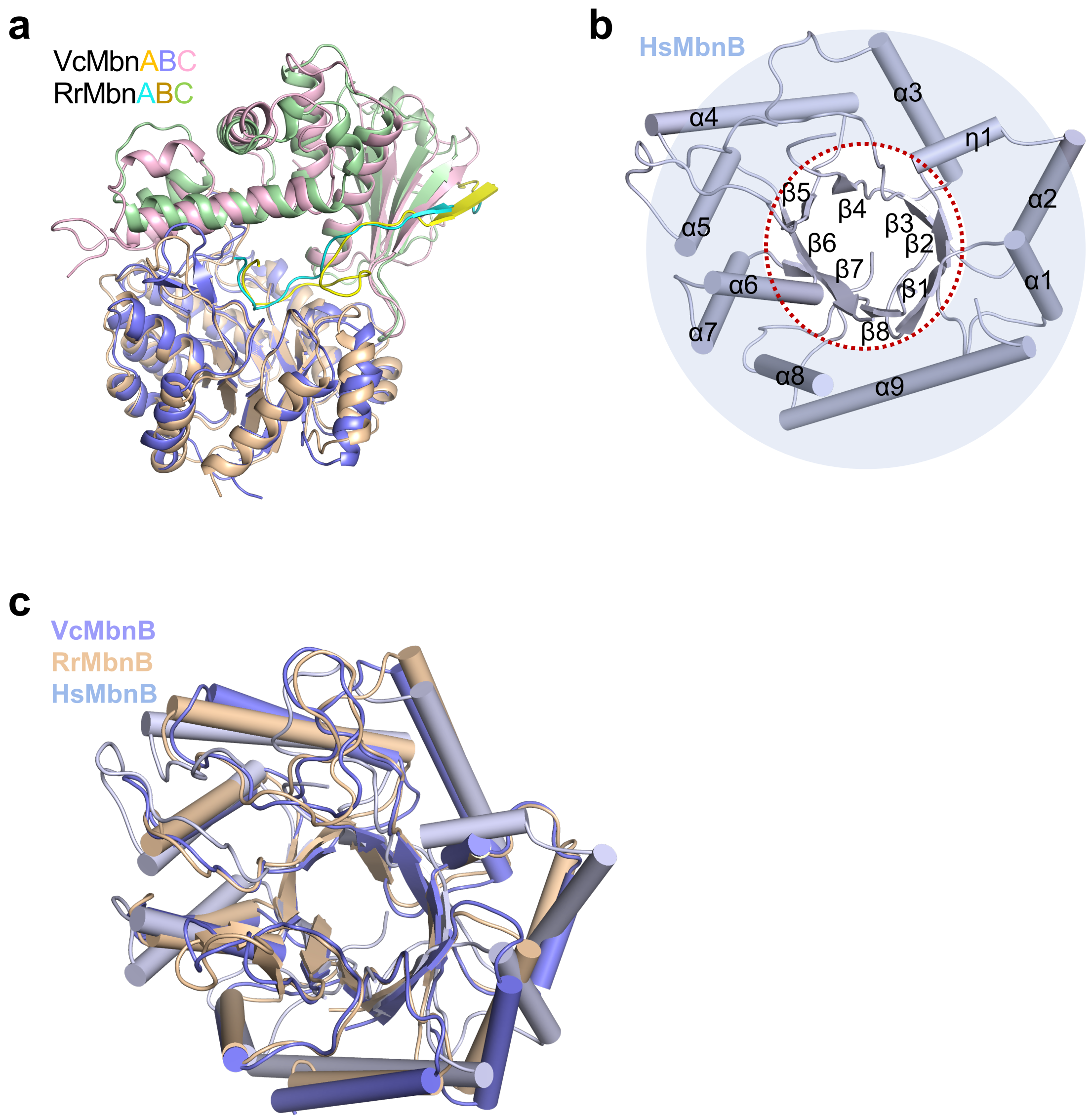

Fig. S6. Structures of VcMbnABC, RrMbnABC, and HsMbnB.

(a) Structural comparison of VcMbnABC and RrMbnABC. MbnA, MbnB, and MbnC are shown in different colors. **(b)** The structure of HsMbnB (PDB ID: 3BWW). Red dotted circle denotes core pore. **(c)** Structural comparison of VcMbnB, RrMbnB and HsMbnB.
