# Supplementary material for: Crystal structure and catalytic mechanism of the MbnBC holoenzyme required for methanobactin biosynthesis
Source: Cell Res. 2022 Feb 2;32(3):302–14. doi: 10.1038/s41422-022-00620-2 (PMC8888699; doi:10.1038/s41422-022-00620-2)
Supplement: Supplementary file 7 — Supplementary Figure S7 [file 41422_2022_620_MOESM7_ESM.pdf]

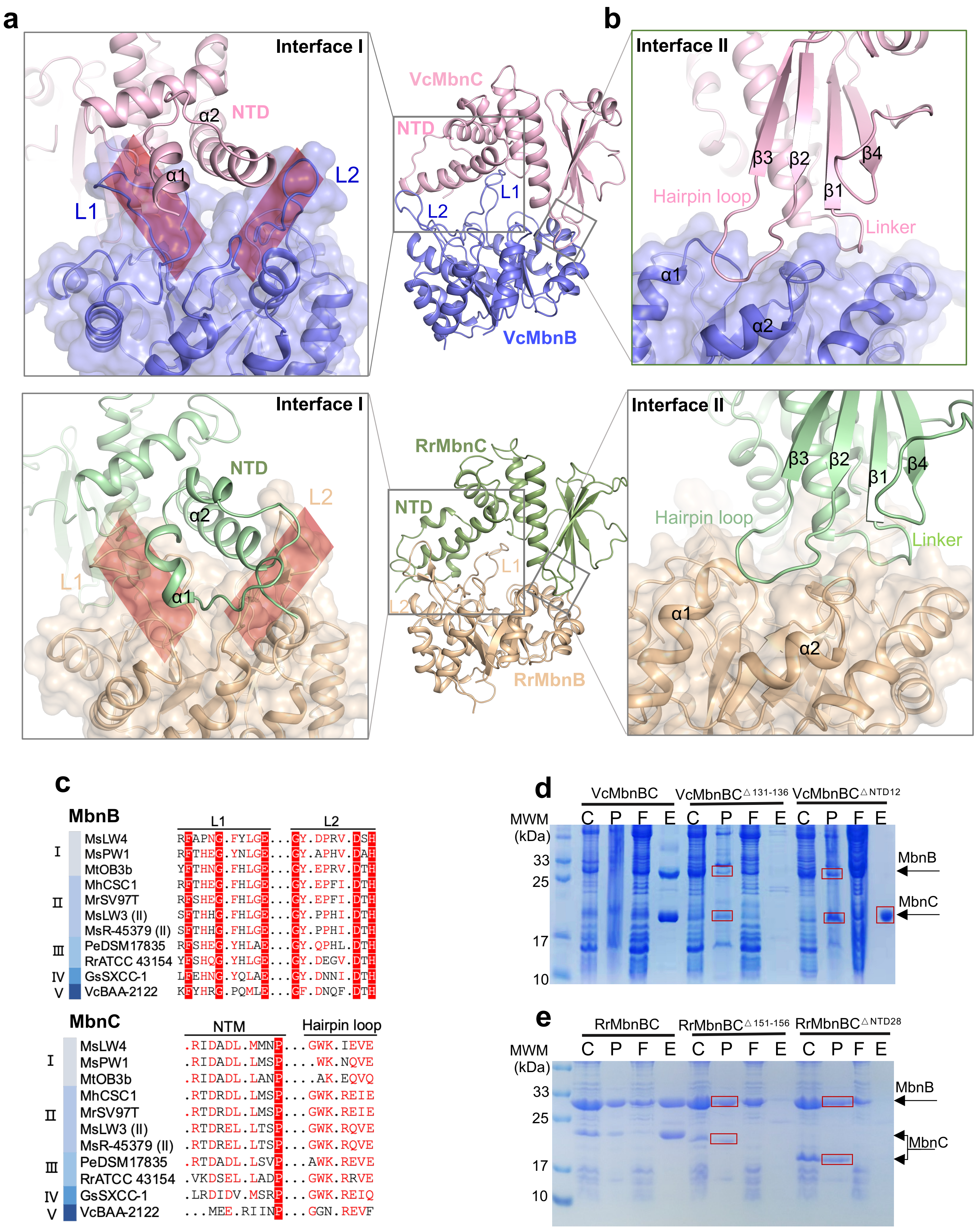

a
Interface I
VcMbnC
NTD
α2
α1
L1
L2
VcMbnB
b
Interface II
β3
β2
β4
β1
Hairpin loop
Linker
α1
α2
Interface I
RrMbnC
NTD
α2
α1
L1
L2
RrMbnB
Interface II
β3
β2
β1
β4
Hairpin loop
Linker
α1
α2
c
MbnB
L1
L2
I MsLW4 RFAPNG.FYLGE...GY.DPRV.DSH
MsPW1 RFTHEG.YNLGE...GY.APHV.DAH
MtOB3b YFTHNG.FHLGE...GY.EPRV.DTH
II MhCSC1 RFTHEG.FHLGE...GY.EPFI.DTH
MrSV97T RFSHEG.FHLGE...GY.EPFI.DTH
MsLW3 (II) SFTHHG.FHLGE...GY.PPHI.DTH
MsR-45379 (II) SFTHHG.FHLGE...GY.PPHI.DTH
III PeDSM17835 RFSHEG.YHLAE...GY.QPHL.DTH
RrATCC 43154 YFSHQG.YHLGE...GY.DEGV.DTH
IV GsSXCC-1 LFEHNG.YQLAE...GY.DNNI.DTH
V VcBAA-2122 KFYHRG.PQMLE...GF.DNQF.DTH
MbnC
NTM
Hairpin loop
I MsLW4 .RIDADL.MMNP...GWK.IEVE
MsPW1 .RIDADL.LMSP....WK.NQVE
MtOB3b .RIDADL.LANP....AK.EQVQ
II MhCSC1 .RTDRDL.LMSP...GWK.REIE
MrSV97T .RTDRDL.LMSP...GWK.REIE
MsLW3 (II) .RTDREL.LTSP...GWK.RQVE
MsR-45379 (II) .RTDREL.LTSP...GWK.RQVE
III PeDSM17835 .RTDADL.LSVP...AWK.REVE
RrATCC 43154 .VKDSEL.LADP...GWK.RRVE
IV GsSXCC-1 .LRDIDV.MSRP...GWK.REIQ
V VcBAA-2122 ...MEE.RIINP...GGN.REVF
d
VcMbnBC
VcMbnBC△131-136
VcMbnBC△NTD12
MWM (kDa)
C P F E C P F E C P F E
33
25
17
10
MbnB
MbnC
e
RrMbnBC
RrMbnBC△151-156
RrMbnBC△NTD28
MWM (kDa)
C P F E C P F E C P F E
33
25
17
10
MbnB
MbnC

Fig. S7. Interactions between MbnB and MbnC.

(a) Interface I between MbnB and MbnC of VcMbnABC (upper) and RrMbnABC (lower). Special structural elements involved in the interactions are labeled. **(b)** Interface II between MbnB and MbnC of VcMbnABC (upper) and RrMbnABC (lower) complexes. Special structural elements involved in interactions are labeled. **(c)** Amino acid sequence alignments of MbnB (top) and MbnC (bottom) mediating the MbnBC interaction. Highly conserved residues are indicated in red. **(d)** Coomassie staining SDS-PAGE gel showing co-expressed VcMbnBC, VcMbnBC$^{\triangle 131\text{-}136}$ and VcMbnBC$^{\triangle \mathrm{NTD12}}$ complexes. C, crude; P, pellet; F, flow through; E, eluent. The MbnB and MbnC positions are indicated with red boxes. **(e)** Coomassie staining SDS-PAGE gel showing co-expressed RrMbnBC, RrMbnBC$^{\triangle 151\text{-}156}$ and the RrMbnBC$^{\triangle \mathrm{NTD28}}$ complexes. C, crude; P, pellet; F, flow through; E, eluent. The MbnB and MbnC positions are indicated with red boxes.
