# Supplementary material for: Crystal structure and catalytic mechanism of the MbnBC holoenzyme required for methanobactin biosynthesis
Source: Cell Res. 2022 Feb 2;32(3):302–14. doi: 10.1038/s41422-022-00620-2 (PMC8888699; doi:10.1038/s41422-022-00620-2)
Supplement: Supplementary file 8 — Supplementary Figure S8 [file 41422_2022_620_MOESM8_ESM.pdf]

a

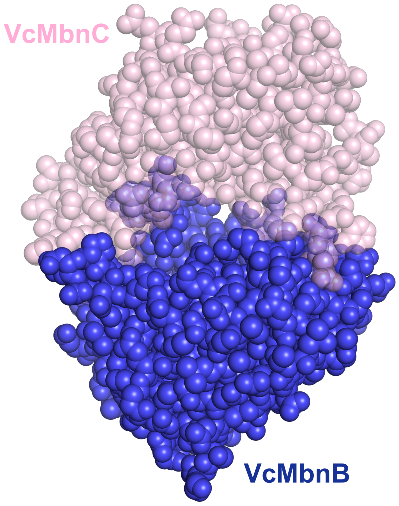

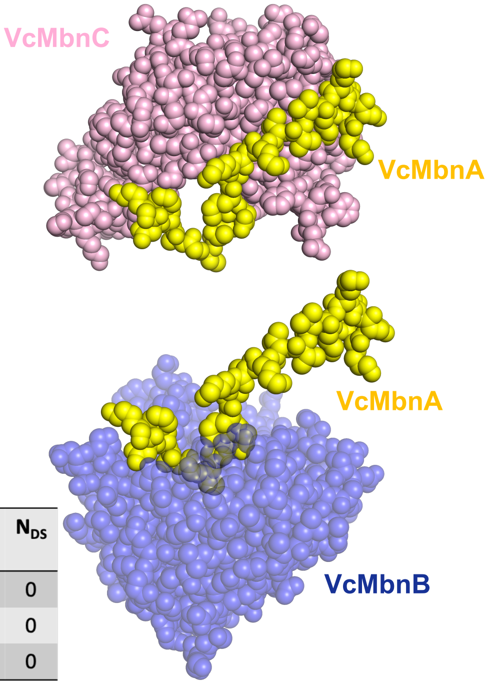

Interfacing structures	Buried area, Å²	Δ^iG, kcal/mol	N_{HB}	N_{SB}	N_{DS}
VcMbnB + VcMbnC	1588.7	-23.6	19	9	0
VcMbnA + VcMbnB	601.5	-7.1	15	5	0
VcMbnA + VcMbnC	978.2	-12.8	11	4	0

b

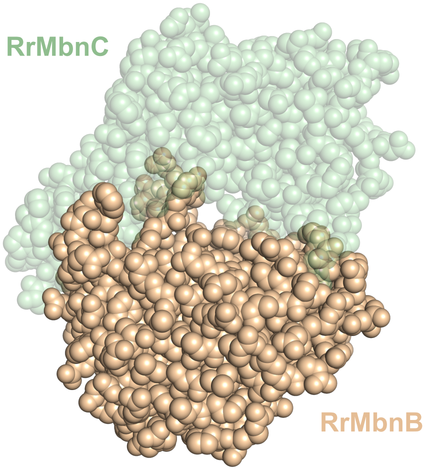

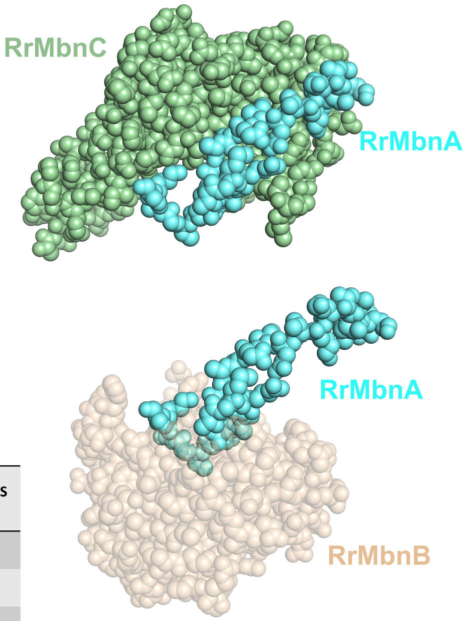

Interfacing structures	Buried area Å²	Δ^iG kcal/mol	N_{HB}	N_{SB}	N_{DS}
RrMbnB + RrMbnC	1791.5	-19.2	19	8	0
RrMbnA + RrMbnB	608.0	-7.2	10	0	0
RrMbnA + RrMbnC	979.2	-3.6	14	2	0

Fig. S8. Interfaces of MbnABC complexes.

(a) Sphere representation of the interaction regions between VcMbnA and VcMbnBC in the VcMbnABC complex. **(b)** Sphere representation of the interaction regions between RrMbnA and RrMbnBC in the RrMbnABC complex. For each interface, values representing the area buried are shown below the structure.
