# Supplementary material for: Crystal structure and catalytic mechanism of the MbnBC holoenzyme required for methanobactin biosynthesis
Source: Cell Res. 2022 Feb 2;32(3):302–14. doi: 10.1038/s41422-022-00620-2 (PMC8888699; doi:10.1038/s41422-022-00620-2)
Supplement: Supplementary file 9 — Supplementary Figure S9 [file 41422_2022_620_MOESM9_ESM.pdf]

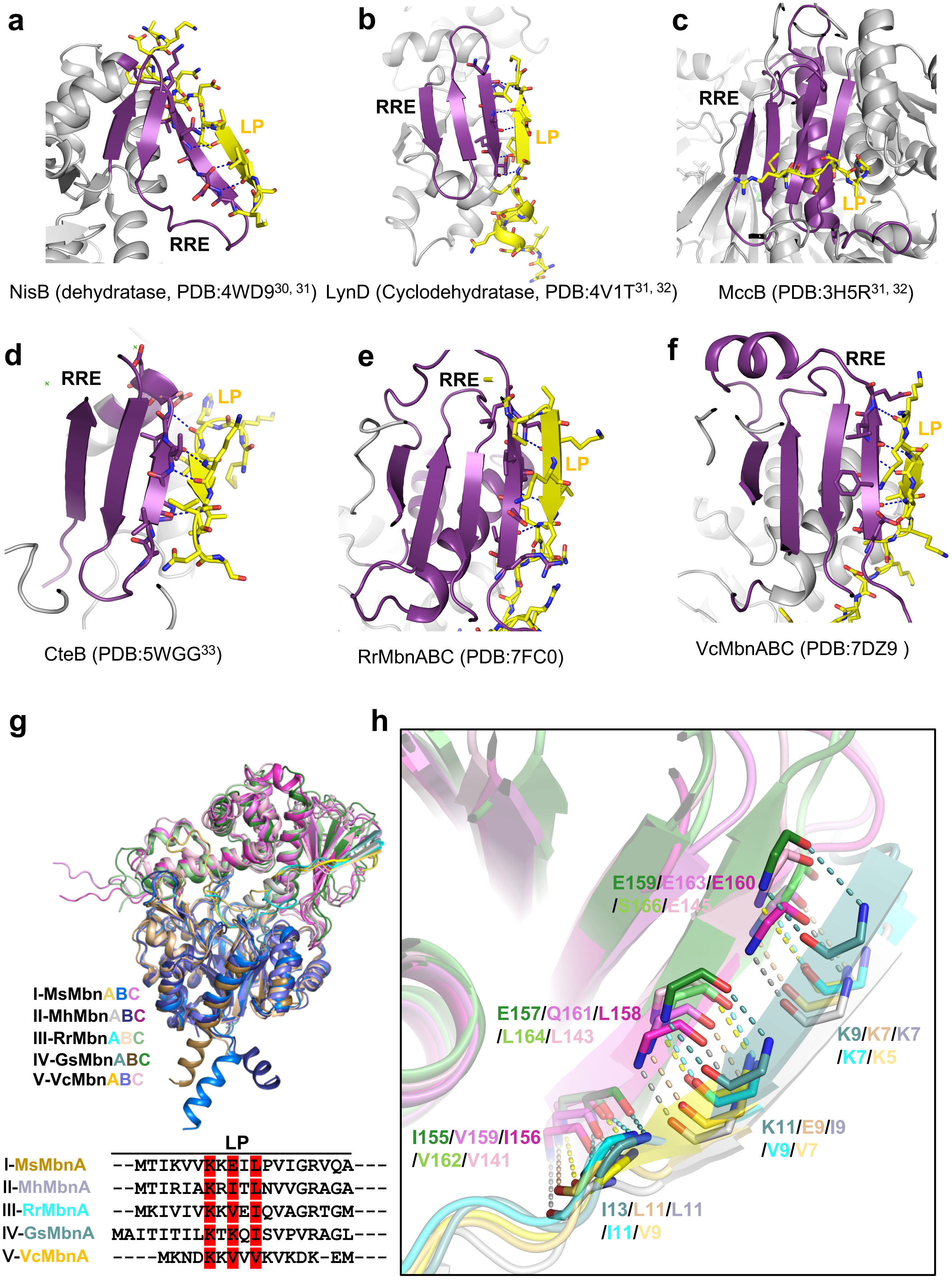
a
LP
RRE
NisB (dehydratase, PDB:4WD9[30, 31])
b
RRE
LP
LynD (Cyclodehydratase, PDB:4V1T[31, 32])
c
RRE
LP
MccB (PDB:3H5R[31, 32])
d
RRE
LP
CteB (PDB:5WGG[33])
e
RRE
LP
RrMbnABC (PDB:7FC0)
f
RRE
LP
VcMbnABC (PDB:7DZ9)
g
I-MsMbnABC
II-MhMbnABC
III-RrMbnABC
IV-GsMbnABC
V-VcMbnABC
LP
I-MsMbnA --MTIKVVKKEILPVIGRVQA---
II-MhMbnA --MTIRIAKRITLNVVGRAGA---
III-RrMbnA --MKIVIVKKVEIQVAGRTGM---
IV-GsMbnA MAITITILKTKQISVPVRAGL---
V-VcMbnA ----MKNDKKVVVKVKDK-EM---
h
E159/E163/E160
/S166/E145
E157/Q161/L158
/L164/L143
K9/K7/K7
/K7/K5
I155/V159/I156
/V162/V141
K11/E9/I9
/V9/V7
I13/L11/L11
/I11/V9

Fig. S9. Leader peptide recognition by RRE.

(a-f) Structural comparison of four leader peptides that bind to RRE[30-33] with RrMbnA and VcMbnA recognized by RrMbnC and VcMbnC, respectively. (a) The lantibiotic nisin (NisB, a dehydratase; PDB: 4WD9)[30, 31]; (b) An antitumor cyanobactins (LynD, a cyclodehydratase; PDB: 4V1T)[31, 32]; (c) The trojan horse antibiotic microcin c7 (Mccb, an adenylating enzyme; PDB: 3H5R)[31, 32]; (d) A sactipeptide (CtsB, sactionine bond-forming enzyme; PDB: 5WGG)[33]; (e) RrMbnABC (PDB: 7FC0) and (f) VcMbnABC (PDB: 7DZ9). The purple β-sheets and gray α-helices constitute a conserved RRE and the precursor peptide is shown in yellow ball-and-stick format. **(g)** Structural comparison of VcMbnABC and RrMbnABC with structures of other groups of MbnABC complexes (predicted with AlphaFold2), namely *Methylosinus* sp. LW4 MbnABC (MsMbnABC), *Mc.hirsuta* CSC1 MbnABC (MhMbnABC) and *Gluconacetobacter* sp. SXCC-1 MbnABC (GsMbnABC). The comparison of the MbnA leader peptide sequences is shown below, these structures and highly conserved residues are indicated in red. **(h)** Conserved interactions between MbnAs and MbnCs. These five MbnAs and MbnCs are consistent with those shown in (g).
