# Supplementary material for: Crystal structure and catalytic mechanism of the MbnBC holoenzyme required for methanobactin biosynthesis
Source: Cell Res. 2022 Feb 2;32(3):302–14. doi: 10.1038/s41422-022-00620-2 (PMC8888699; doi:10.1038/s41422-022-00620-2)
Supplement: Supplementary file 10 — Supplementary Figure S10 [file 41422_2022_620_MOESM10_ESM.pdf]

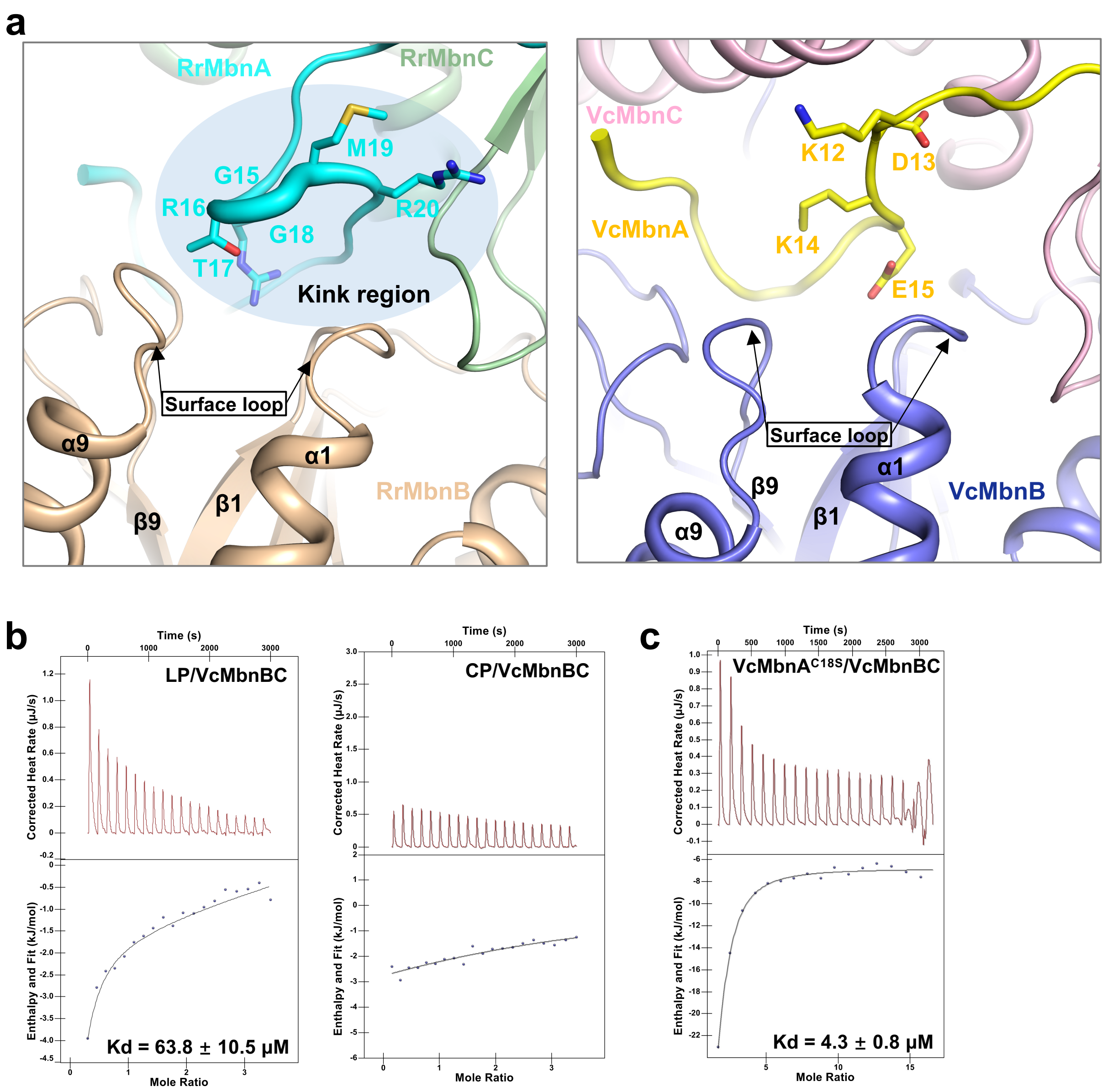

Fig. S10. MbnA variants recognition by MbnBC.

(a) The C-terminal half of the RrMbnA LP (residues 15–20) forms a short kink that interacts with the surface loop region of RrMbnB compared to VcMbnA. The kink is shown as a putty cartoon representation, and the surface loops are also indicated with arrows. **(b)** ITC measurement of the binding affinity between VcMbnA variants (LP or CP) and VcMbnBC. The upper panel shows the original titration traces. **(c)** ITC measurement of the binding affinity between VcMbnA^{C18S} and VcMbnBC. The upper panel shows the original titration traces.
