# Supplementary material for: Crystal structure and catalytic mechanism of the MbnBC holoenzyme required for methanobactin biosynthesis
Source: Cell Res. 2022 Feb 2;32(3):302–14. doi: 10.1038/s41422-022-00620-2 (PMC8888699; doi:10.1038/s41422-022-00620-2)
Supplement: Supplementary file 11 — Supplementary Figure S11 [file 41422_2022_620_MOESM11_ESM.pdf]

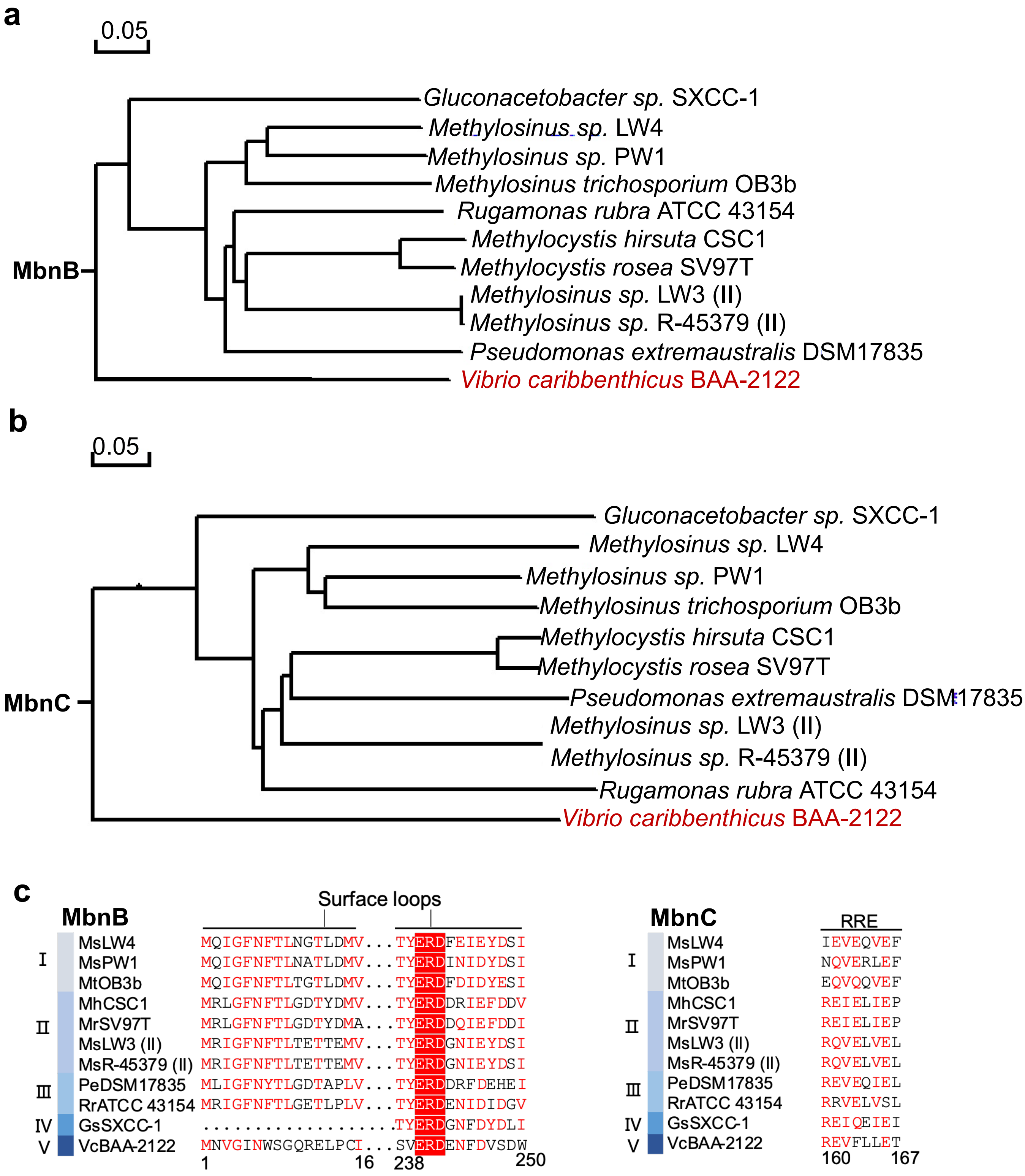
a
0.05
MbnB
Gluconacetobacter sp. SXCC-1
Methylosinus sp. LW4
Methylosinus sp. PW1
Methylosinus trichosporium OB3b
Rugamonas rubra ATCC 43154
Methylocystis hirsuta CSC1
Methylocystis rosea SV97T
Methylosinus sp. LW3 (II)
Methylosinus sp. R-45379 (II)
Pseudomonas extremaustralis DSM17835
Vibrio caribbenthicus BAA-2122
b
0.05
MbnC
Gluconacetobacter sp. SXCC-1
Methylosinus sp. LW4
Methylosinus sp. PW1
Methylosinus trichosporium OB3b
Methylocystis hirsuta CSC1
Methylocystis rosea SV97T
Pseudomonas extremaustralis DSM17835
Methylosinus sp. LW3 (II)
Methylosinus sp. R-45379 (II)
Rugamonas rubra ATCC 43154
Vibrio caribbenthicus BAA-2122
c
MbnB
Surface loops
I
MsLW4 MQIGFNFTLNGTLDMV...TYERDFEIEYDSI
MsPW1 MQIGFNFTLNATLDMV...TYERDINIDYDSI
MtOB3b MQIGFNFTLTGTLDMV...TYERDFDIDYESI
II
MhCSC1 MRLGFNFTLGDTYDMV...TYERDDRIEFDDV
MrSV97T MRLGFNFTLGDTYDMA...TYERDDQIEFDDI
MsLW3 (II) MRIGFNFTLTETTEMV...TYERDGNIEYDSI
MsR-45379 (II) MRIGFNFTLTETTEMV...TYERDGNIEYDSI
III
PeDSM17835 MLIGFNYTLGDTAPLV...TYERDDRFDEHEI
RrATCC 43154 MRIGFNFTLGETLPLV...TYERDENIDIDGV
IV
GsSXCC-1 ...................TYERDGNFDYDLI
V
VcBAA-2122 MNVGINWSGQRELPCI...SVERDENFDVSDW
1 16 238 250
MbnC
RRE
I
MsLW4 IEVEQVEF
MsPW1 NQVERLEF
MtOB3b EQVQQVEF
II
MhCSC1 REIELIEP
MrSV97T REIELIEP
MsLW3 (II) RQVELVEL
MsR-45379 (II) RQVELVEL
III
PeDSM17835 REVEQIEL
RrATCC 43154 RRVELVSL
IV
GsSXCC-1 REIQEIEI
V
VcBAA-2122 REVFLLET
160 167

Fig. S11. Phylogenetic analysis of VcMbnBC among Mbn operons.

(a-b) Phylogenetic analysis of MbnBs and MbnCs from species in each of the five Mbn groups: *Methylosinus* sp. LW4 (Group I); *Methylosinus* sp. PW1(Group I); *Methylosinus trichosporium* OB3b (Group I); *Mc.hirsuta* CSC1 (Group IIa); *Methylocystis rosea* SV97T (Group IIa); *Methylosinus* sp. LW3 (II) (Group IIb); *Methylosinus* sp. R-45379 (II) (Group IIb); *Pseudomonas extremaustralis* DSM17835 (Group III); *Rugamonas rubra* ATCC 43154 (Group III); *Gluconacetobacter* sp. SXCC-1 (Group IV) and *Vibrio caribbenthicus* BAA-2122 (Group V). The phylogenetic tree was constructed by DNAMAN software. **(c)** Sequence alignment of the MbnA recognition regions in MbnBCs. The conserved amino acids are labeled in red and highlighted.
