# Supplementary material for: Crystal structure and catalytic mechanism of the MbnBC holoenzyme required for methanobactin biosynthesis
Source: Cell Res. 2022 Feb 2;32(3):302–14. doi: 10.1038/s41422-022-00620-2 (PMC8888699; doi:10.1038/s41422-022-00620-2)
Supplement: Supplementary file 12 — Supplementary Figure S12 [file 41422_2022_620_MOESM12_ESM.pdf]

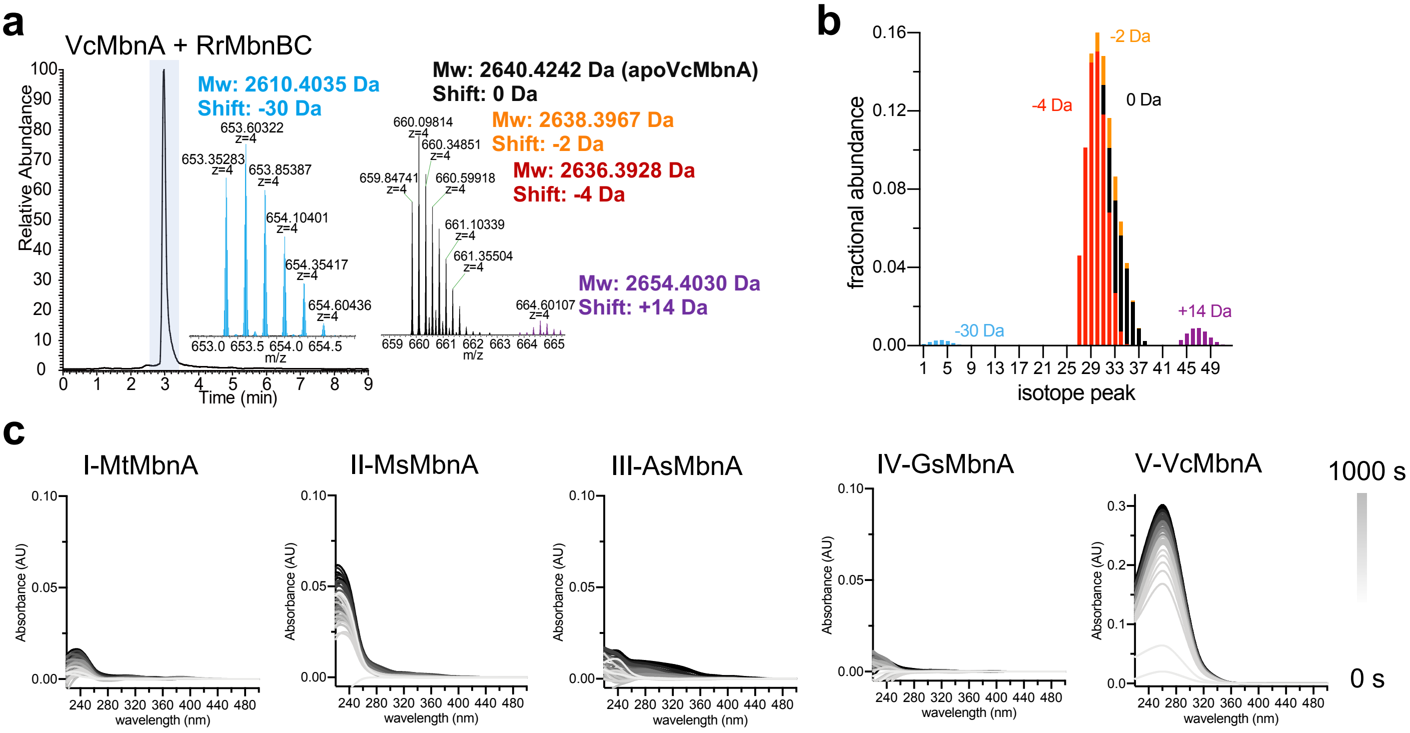

Fig. S12. Promiscuous recognition of RrMbnBC.

(a) Total ion chromatogram and mass spectra (inset) of VcMbnA modified by RrMbnBC. The mass shifts are indicated. **(b)** VcMbnA modified by RrMbnBC. The relative abundance of the components of modified VcMbnA components are represented in the histogram on the right: the unmodified state is 22%, the -4 Da state is 67%, the -30 Da state is 6%, and the +14 Da state is 5%. **(c)** Reaction of VcMbnBC with MbnAs of species from the five Mbn groups (*Methylosinus trichosporium* OB3b (MtMbnA, Group I), *Methylocystis* sp. SC2 (MsMbnA, Group II), *Azospirillum* sp. B510 (AsMbnA, Group III), *Gluconacetobacter* sp. SXCC-1 (GsMbnA, Group IV) and *Vibrio caribbenthicus* BAA-2122 (VcMbnA, Group V)).
