# Supplementary material for: Crystal structure and catalytic mechanism of the MbnBC holoenzyme required for methanobactin biosynthesis
Source: Cell Res. 2022 Feb 2;32(3):302–14. doi: 10.1038/s41422-022-00620-2 (PMC8888699; doi:10.1038/s41422-022-00620-2)
Supplement: Supplementary file 13 — Supplementary Figure S13 [file 41422_2022_620_MOESM13_ESM.pdf]

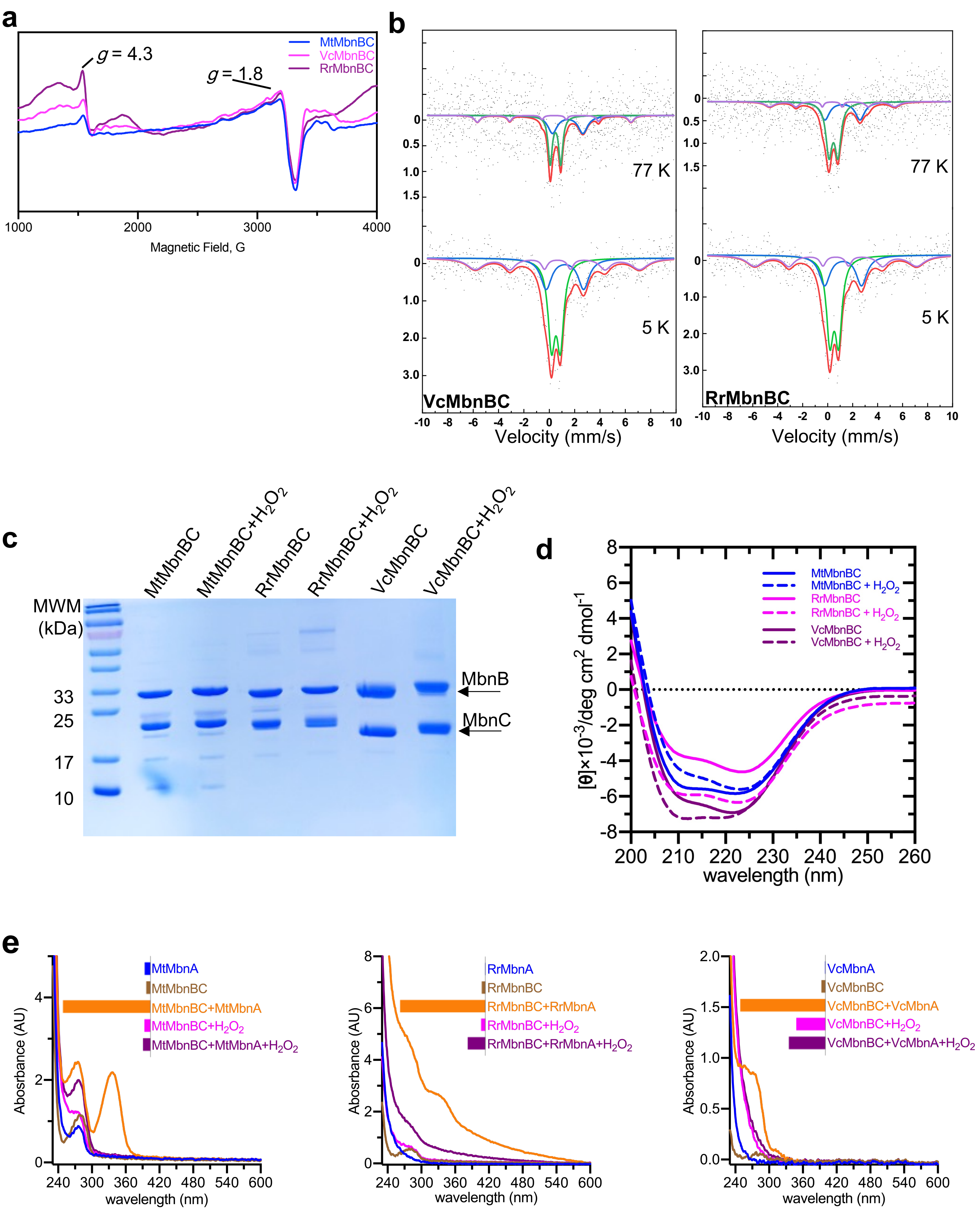
a
g = 4.3
g = 1.8
MtMbnBC
VcMbnBC
RrMbnBC
1000
2000
3000
4000
Magnetic Field, G
b
77 K
5 K
VcMbnBC
RrMbnBC
Velocity (mm/s)
c
MtMbnBC
MtMbnBC+H2O2
RrMbnBC
RrMbnBC+H2O2
VcMbnBC
VcMbnBC+H2O2
MWM
(kDa)
33
25
17
10
MbnB
MbnC
d
MtMbnBC
MtMbnBC + H2O2
RrMbnBC
RrMbnBC + H2O2
VcMbnBC
VcMbnBC + H2O2
[θ]×10-3/deg cm2 dmol-1
wavelength (nm)
e
MtMbnA
MtMbnBC
MtMbnBC+MtMbnA
MtMbnBC+H2O2
MtMbnBC+MtMbnA+H2O2
RrMbnA
RrMbnBC
RrMbnBC+RrMbnA
RrMbnBC+H2O2
RrMbnBC+RrMbnA+H2O2
VcMbnA
VcMbnBC
VcMbnBC+VcMbnA
VcMbnBC+H2O2
VcMbnBC+VcMbnA+H2O2
Absorbance (AU)
wavelength (nm)

**Fig. S13. Analysis of oxygen-needed tri-iron center.**

**(a)** EPR spectra of aerobically purified MtMbnBC, VcMbnBC and RrMbnBC collected at 5 K. EPR measurement conditions are described in the Methods. The signals near $g$ = 4.3 and 1.8 are indicated. MtMbnBC, VcMbnBC and RrMbnBC are shown in blue, pink and purple, respectively. **(b)** $^{57}$Fe-Mössbauer spectra of aerobically purified VcMbnBC (left) and RrMbnBC (right) recorded at 77 K/5 K with a 0 field. The raw data are presented as black dots. A three-component simulation is overlaid (red solid line), comprising three components (blue, green, violet solid lines), consistent with three unique sites in a triferric cluster, $Fe^{II}$, $Fe^{III}$ and $Fe^{III}/Fe^{II}$. The corresponding parameters are summarized in Supplementary Table S3. **(c)** $H_2O_2$-treated MtMbnBC, RrMbnBC and VcMbnBC further purified using gel filtration chromatography and analyzed with Coomassie staining SDS-PAGE gels. **(d)** The $H_2O_2$-treated proteins were detected by circular dichroism (CD) spectroscopy. **(e)** UV-Vis absorption spectra of MbnA modified by MbnBC treated with excess $H_2O_2$. From left to right are MtMbnA, VcMbnA and RrMbnA. (Inset) The activities of these proteins are shown by UV absorbance at 335 nm, 270 nm and 335 nm.
