# Supplementary material for: Crystal structure and catalytic mechanism of the MbnBC holoenzyme required for methanobactin biosynthesis
Source: Cell Res. 2022 Feb 2;32(3):302–14. doi: 10.1038/s41422-022-00620-2 (PMC8888699; doi:10.1038/s41422-022-00620-2)
Supplement: Supplementary file 14 — Supplementary Figure S14 [file 41422_2022_620_MOESM14_ESM.pdf]

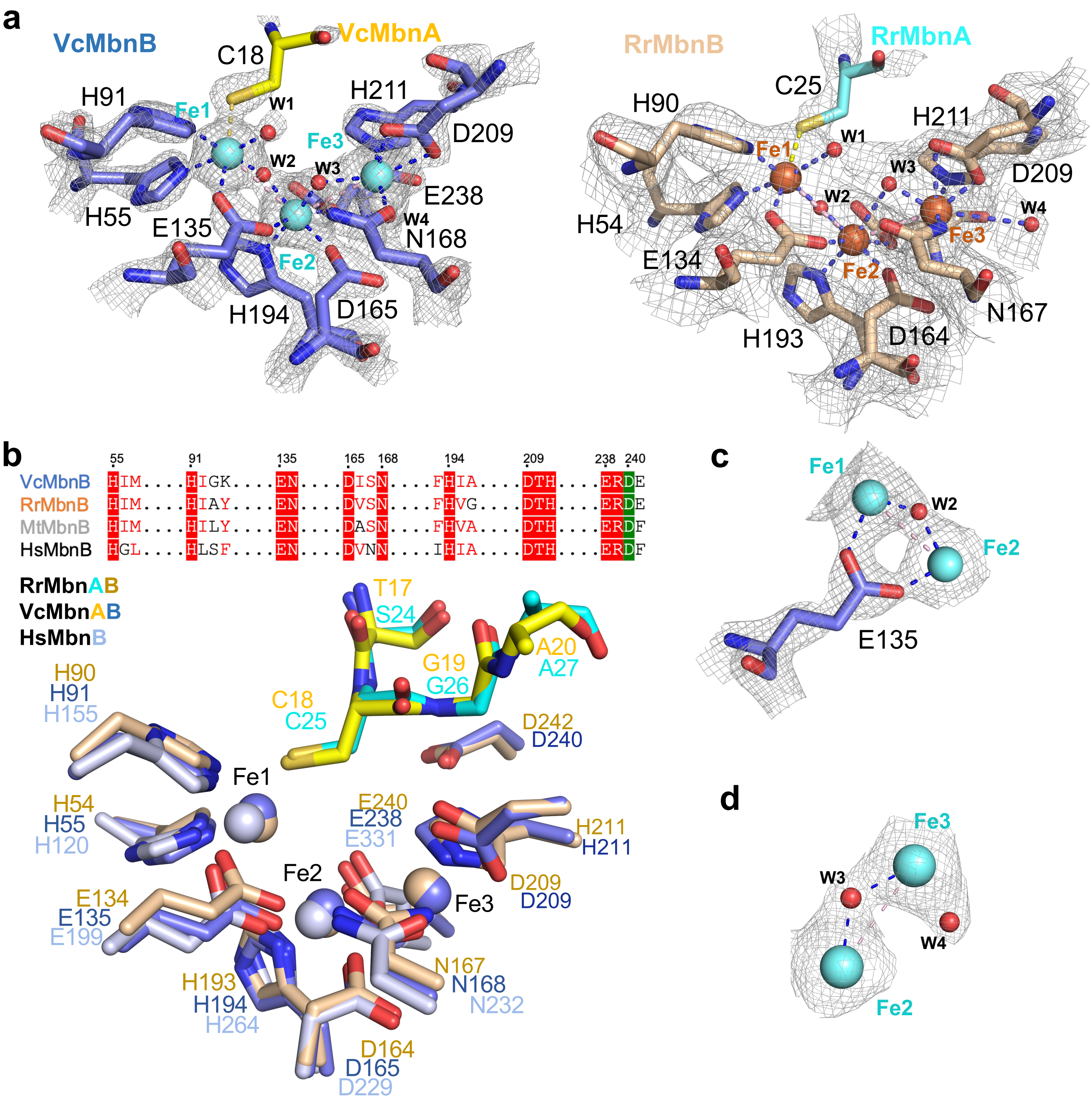

a
VcMbnB
VcMbnA
C18
H91
Fe1
W1
H211
D209
Fe3
W2
W3
E238
H55
E135
W4
N168
Fe2
H194
D165
RrMbnB
RrMbnA
C25
H90
W1
H211
Fe1
D209
W3
W2
W4
H54
Fe3
E134
Fe2
N167
H193
D164
b
55 91 135 165 168 194 209 238 240
VcMbnB HIM....HIGK....EN....DISN....FHIA....DTH....ERDE
RrMbnB HIM....HIAY....EN....DVSN....FHVG....DTH....ERDE
MtMbnB HIM....HILY....EN....DASN....FHVA....DTH....ERDF
HsMbnB HGL....HLSF....EN....DVNN....IHIA....DTH....ERDF
RrMbnAB
VcMbnAB
HsMbnB
T17
S24
A20
A27
G19
G26
C18
C25
H90
H91
H155
D242
D240
Fe1
H54
H55
H120
E240
E238
E331
H211
H211
Fe2
D209
D209
Fe3
E134
E135
E199
H193
H194
H264
N167
N168
N232
D164
D165
D229
c
Fe1
W2
Fe2
E135
d
Fe3
W3
W4
Fe2

Fig. S14. Active sites of MbnBs.

(a) Three iron atoms (Fe1, Fe2 and Fe3) are six-coordinated with octahedral geometry. The tri-iron active site with binding ligand residues, Cys18 (VcMbnA) or Cys25 (RrMbnA), iron, and water molecules are contoured with a 2Fo-Fc map at 2.0 σ. **(b)** Sequence alignment of MtMbnB, RrMbnB, VcMbnB, and HsMbnB with conserved iron binding residues indicated. Conserved and catalytic residues are highlighted in red and green, respectively. The tri-iron center of VcMbnB and RrMbnB and the di-iron center of HsMbnB are represented as spheres with the corresponding ligand amino acids shown as sticks. Thr17-Ala20 of VcMbnA and Ser24-Ala27 of RrMbnA form the curve portion. **(c)** Glu135 of VcMbnB provides a bidentate carboxylate ligand that bridges Fe1 and Fe2. **(d)** Solvent molecules complete the coordination of Fe2 and Fe3, with one water molecule coupling the two metal ions.
