# Supplementary material for: Crystal structure and catalytic mechanism of the MbnBC holoenzyme required for methanobactin biosynthesis
Source: Cell Res. 2022 Feb 2;32(3):302–14. doi: 10.1038/s41422-022-00620-2 (PMC8888699; doi:10.1038/s41422-022-00620-2)
Supplement: Supplementary file 15 — Supplementary Figure S15 [file 41422_2022_620_MOESM15_ESM.pdf]

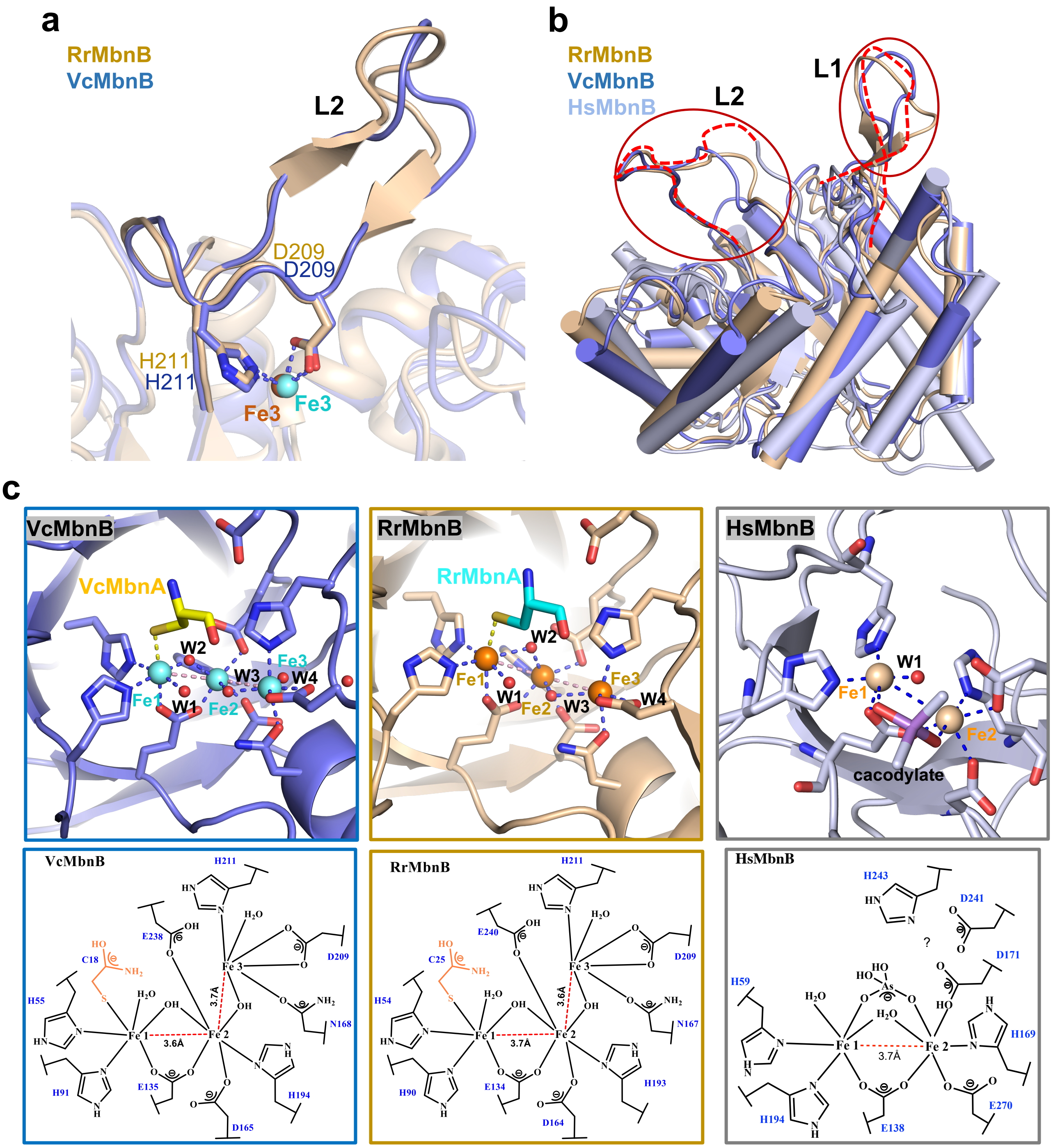
a
RrMbnB
VcMbnB
L2
D209
D209
H211
H211
Fe3
Fe3
b
RrMbnB
VcMbnB
HsMbnB
L2
L1
c
VcMbnB
VcMbnA
W2
W3
W4
Fe3
Fe1
W1
Fe2
RrMbnB
RrMbnA
W2
Fe1
W1
Fe2
W3
Fe3
W4
HsMbnB
W1
Fe1
Fe2
cacodylate
VcMbnB
H211
E238
D209
C18
H55
H91
E135
D165
H194
N168
Fe 1
Fe 2
Fe 3
3.6Å
3.7Å
RrMbnB
H211
E240
D209
C25
H54
H90
E134
D164
H193
N167
Fe 1
Fe 2
Fe 3
3.7Å
3.6Å
HsMbnB
H243
D241
?
D171
H59
H169
H194
E138
E270
Fe 1
Fe 2
3.7Å

Fig. S15. Structural comparison of RrMbnB, VcMbnB and HsMbnB.

(a) Structural comparison of RrMbnB and VcMbnB. The conserved residues of L2 loop coordinated with Fe3 are represented as sticks. **(b)** Structural comparison of VcMbnB, RrMbnB and HsMbnB. The equivalent L1 and L2 loops of VcMbnB and RrMbnB, which are represented as red dashed lines and highlighted by red circles, are disordered in the HsMbnB structure. **(c)** Comparison of tri-iron active sites of VcMbnB, RrMbnB and HsMbnB. The iron sites are labeled Fe1, Fe2, and Fe3 to illustrate the potential amino acid ligands with these ions throughout the manuscript. The amino acid ligands to Fe1 and Fe2 sites are conserved in these structures; however, the two residues Asp209 and His211 in the disordered extended loop of HsMbnB result in a di-iron center.
