# Supplementary figures and images for: Crystal structure and catalytic mechanism of the MbnBC holoenzyme required for methanobactin biosynthesis
Source: Cell Res. 2022 Feb 2;32(3):302–14. doi: 10.1038/s41422-022-00620-2 (PMC8888699; doi:10.1038/s41422-022-00620-2)

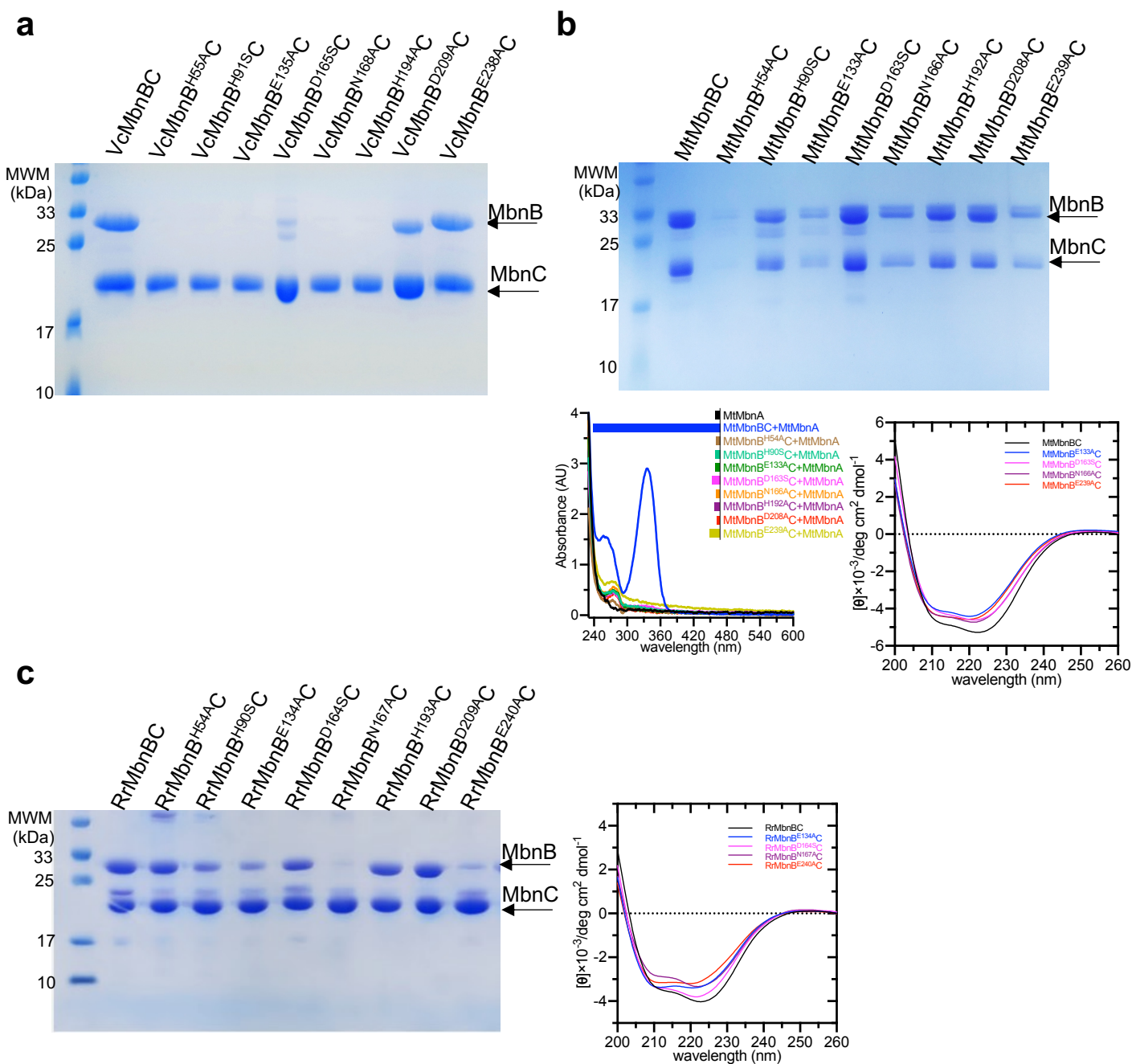

Supplement: Supplementary file 16 — Supplementary Figure S16 [file 41422_2022_620_MOESM16_ESM.pdf]
