# Supplementary material for: Crystal structure and catalytic mechanism of the MbnBC holoenzyme required for methanobactin biosynthesis
Source: Cell Res. 2022 Feb 2;32(3):302–14. doi: 10.1038/s41422-022-00620-2 (PMC8888699; doi:10.1038/s41422-022-00620-2)
Supplement: Supplementary file 17 — Supplementary Figure S17 [file 41422_2022_620_MOESM17_ESM.pdf]

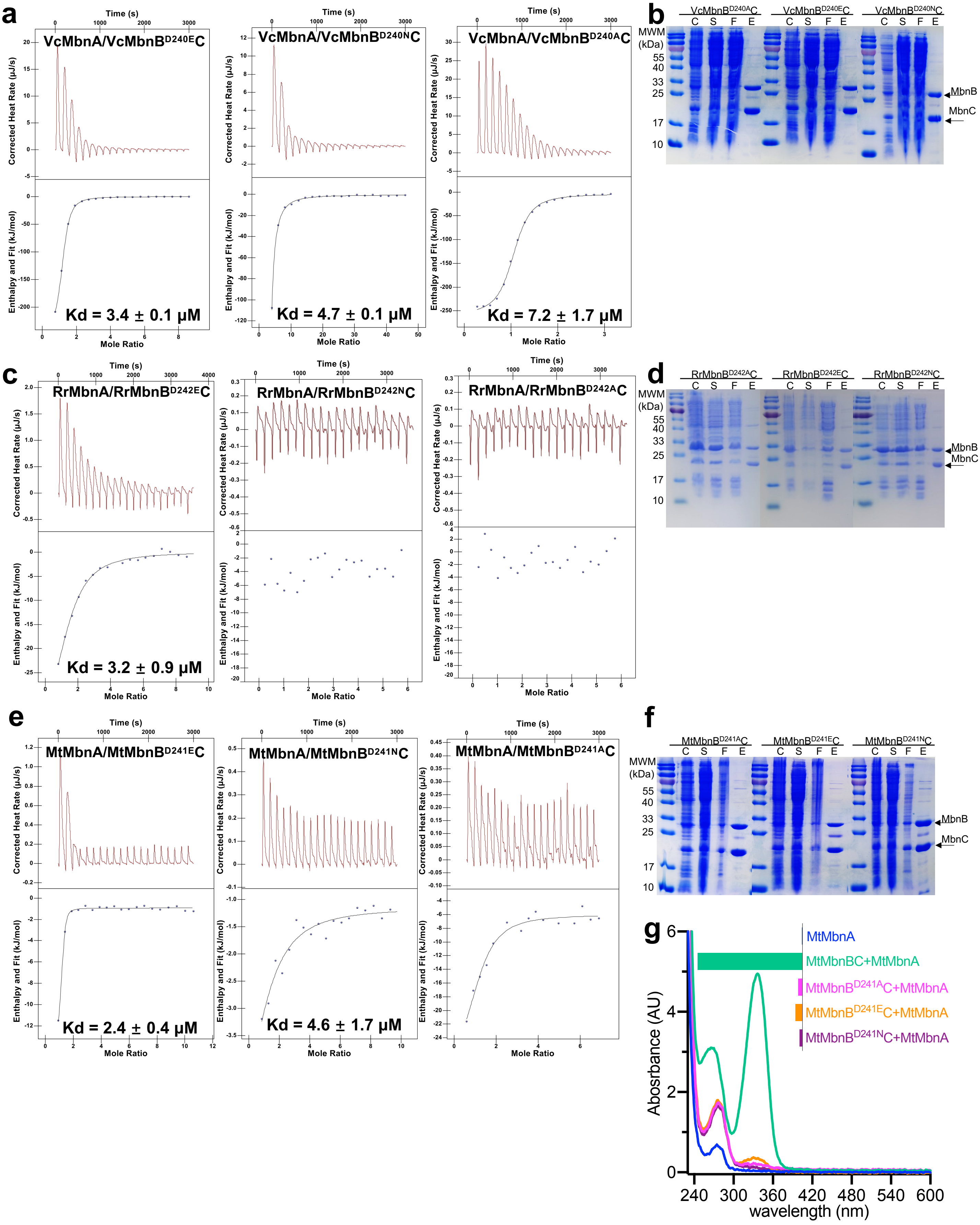
a
VcMbnA/VcMbnBD240EC
Kd = 3.4 ± 0.1 μM
VcMbnA/VcMbnBD240NC
Kd = 4.7 ± 0.1 μM
VcMbnA/VcMbnBD240AC
Kd = 7.2 ± 1.7 μM
Time (s)
Corrected Heat Rate (μJ/s)
Enthalpy and Fit (kJ/mol)
Mole Ratio
b
VcMbnBD240AC
VcMbnBD240EC
VcMbnBD240NC
C S F E
MWM (kDa)
55
40
33
25
17
10
MbnB
MbnC
c
RrMbnA/RrMbnBD242EC
Kd = 3.2 ± 0.9 μM
RrMbnA/RrMbnBD242NC
RrMbnA/RrMbnBD242AC
d
RrMbnBD242AC
RrMbnBD242EC
RrMbnBD242NC
e
MtMbnA/MtMbnBD241EC
Kd = 2.4 ± 0.4 μM
MtMbnA/MtMbnBD241NC
Kd = 4.6 ± 1.7 μM
MtMbnA/MtMbnBD241AC
f
MtMbnBD241AC
MtMbnBD241EC
MtMbnBD241NC
g
MtMbnA
MtMbnBC+MtMbnA
MtMbnBD241AC+MtMbnA
MtMbnBD241EC+MtMbnA
MtMbnBD241NC+MtMbnA
Absorbance (AU)
wavelength (nm)
240 300 360 420 480 540 600

Fig. S17. Characterization of the catalytic residue Asp of MbnBs.

(a) ITC measurement of the binding affinity between VcMbnBC variants (VcMbnB^{D240E}C, VcMbnB^{D240N}C, and VcMbnB^{D240A}C) and VcMbnA. **(b)** Co-expression of VcMbnBC variants (VcMbnB^{D240E}C, VcMbnB^{D240N}C, and VcMbnB^{D240A}C) as detected by SDS-PAGE. **(c)** ITC measurement of the binding affinity between RrMbnBC variants (RrMbnB^{D242E}C, RrMbnB^{D242N}C, and RrMbnB^{D242A}C) and RrMbnA. **(d)** Co-expression of RrMbnBC variants (RrMbnB^{D242E}C, RrMbnB^{D242N}C, and RrMbnB^{D242A}C) as detected by SDS-PAGE. **(e)** ITC measurement of the binding affinity between MtMbnBC variants (MtMbnB^{D241E}C, MtMbnB^{D241N}C and MtMbnB^{D241A}C) and MtMbnA. **(f)** Co-expression of MtMbnBC variants (MtMbnB^{D241E}C, MtMbnB^{D241N}C, and MtMbnB^{D241A}C) as detected by SDS-PAGE. The upper panel shows the original titration traces. **(g)** UV-Vis spectra detection of MtMbnBC variant (MtMbnB^{D241A}C, MtMbnB^{D241E}C, and MtMbnB^{D241N}C) activity.
