# Supplementary material for: Crystal structure and catalytic mechanism of the MbnBC holoenzyme required for methanobactin biosynthesis
Source: Cell Res. 2022 Feb 2;32(3):302–14. doi: 10.1038/s41422-022-00620-2 (PMC8888699; doi:10.1038/s41422-022-00620-2)
Supplement: Supplementary file 18 — Supplementary Table S1 [file 41422_2022_620_MOESM18_ESM.pdf]

**Table S1. Data collect and refinement statistics**

| | VcMbnABC<br>PDB ID: 7DZ9 | RrMbnABC<br>PDB ID: 7FC0 |
|---|---|---|
| **Data collect** | | |
| Space group | P 1 $2_1$ 1 | P $4_3$ |
| **Cell dimensions** | | |
| a, b, c (Å) | 81.915, 69.065, 82.573 | 173.968, 173.968, 61.186 |
| α, β, γ (°) | 90.00, 93.59, 90.00 | 90.00, 90.00, 90.00 |
| **Resolution (Å)** | 45.27 - 2.2 (2.279 - 2.2) | 61.51- 2.64 (2.738 - 2.64) |
| $R_{merge}$ **(%)** | 11.83 | 20.53 |
| $R_{pim}$ **(%)** | 3.2 | 5.9 |
| $I/\sigma$ ($I$) | 18.78 (6.35) | 6.46 (0.49) |
| **Completeness (%)** | 99.37 (99.64) | 99.79 (99.85) |
| **Redundancy** | 13.7 (13.7) | 13.0 (12.80) |
| **Refinement** | | |
| **No. reflections** | 46634 (4648) | 54084 (5328) |
| $R_{work}/R_{free}$ **(%)** | 18.17/22.32 | 18.83/22.48 |
| **No. atoms** | 7781 | 7726 |
| Protein | 7562 | 7602 |
| Ligand | 6 | 6 |
| solvent | 213 | 118 |
| **Average B-factors** | 30.03 | 87.92 |
| **Ramachandran favored (%)** | 98.47 | 97.49 |
| **Ramachandran allowed (%)** | 1.53 | 2.51 |
| **Ramachandran outliers (%)** | 0 | 0 |
| **R.m.s deviations** | | |
| Bond length (Å) | 0.003 | 0.017 |
| Bond angles (°) | 0.52 | 2.09 |

Value in parentheses is for the highest-resolution shell.
