# Supplementary material for: Crystal structure and catalytic mechanism of the MbnBC holoenzyme required for methanobactin biosynthesis
Source: Cell Res. 2022 Feb 2;32(3):302–14. doi: 10.1038/s41422-022-00620-2 (PMC8888699; doi:10.1038/s41422-022-00620-2)
Supplement: Supplementary file 19 — Supplementary Table S2 [file 41422_2022_620_MOESM19_ESM.pdf]

Table S2. Inductively couple plasma mass spectrometry (ICP-MS)

Analyte	Fe	Ni	Co	Cu
MtMbnBC	1.645 ± 0.032	0.106 ± 0.082	< 0.044	< 0.041
VcMbnBC	2.834 ± 0.186	0.243 ± 0.013	< 0.071	<0.058
RrMbnBC	1.408 ± 0.198	0.093 ± 0.015	< 0.029	< 0.027
RrMbnABC	2.798 ± 0.361	0.145 ± 0.068	< 0.041	< 0.038

The molar equivalents of various metals per hetero-MbnBC dimer were measured. All the values are mean and standard deviation of the measured metal concentrations for three independent protein preparations.
