# Supplementary material for: Crystal structure and catalytic mechanism of the MbnBC holoenzyme required for methanobactin biosynthesis
Source: Cell Res. 2022 Feb 2;32(3):302–14. doi: 10.1038/s41422-022-00620-2 (PMC8888699; doi:10.1038/s41422-022-00620-2)
Supplement: Supplementary file 20 — Supplementary Table S3 [file 41422_2022_620_MOESM20_ESM.pdf]

Table S3. Mössbauer Simulation Parameters of the tri-iron cluster of VcMbnBC and RrMbnBC

Sample	Temperature (K)	δ[a] (mm/s)	$\triangle E_Q$[b] (mm/s)	M.F.[c] (T)	Relative Amount (%)	Valence state of Fe
VcMbnBC	77	1.45	2.40		28.93	Fe^{II}
		0.50	0.83		54.16	Fe^{III}
		0.38		37.6	16.91	Fe^{III}/ Fe^{II}
	5	1.23	2.96		17.07	Fe^{II}
		0.53	0.70		54.51	Fe^{III}
		0.65		40.3	28.42	Fe^{III}/ Fe^{II}
RrMbnBC	77	1.18	2.80		37.53	Fe^{II}
		0.46	0.72		44.56	Fe^{III}
		0.36		31.1	17.91	Fe^{III}/ Fe^{II}
	5	1.28	3.10		26.21	Fe^{II}
		0.50	0.66		44.99	Fe^{III}
		0.84		29.8	28.79	Fe^{III}/ Fe^{II}

[a]Isomer shift versus α-iron at RT

[b]Quadrupole splitting

[c]Magnetic field (intrinsic)
