# Supplementary material for: Crystal structure and catalytic mechanism of the MbnBC holoenzyme required for methanobactin biosynthesis
Source: Cell Res. 2022 Feb 2;32(3):302–14. doi: 10.1038/s41422-022-00620-2 (PMC8888699; doi:10.1038/s41422-022-00620-2)

Table S4. Determination of iron levels by 1, 10-phenanthroline colorimetric

Protein	preparation	Concentration (mM)		Molecular ratio of Fe^{II}/Total Fe (%)
		Fe^{II}	Total Fe	
**MtMbnBC**	aerobically	0.67×10^{-3}	2.72×10^{-3}	24.63
	anaerobically	0.50×10^{-3}	1.91×10^{-3}	26.18
**VcMbnBC**	aerobically	2.57×10^{-3}	7.13×10^{-3}	36.04
	anaerobically	3.35×10^{-3}	9.01×10^{-3}	37.19
**RrMbnBC**	aerobically	1.45×10^{-3}	4.19×10^{-3}	34.60
	anaerobically	1.53×10^{-3}	4.74×10^{-3}	32.28
**RrMbnABC**	aerobically	1.88×10^{-3}	4.91×10^{-3}	38.29
	anaerobically	1.94×10^{-3}	5.43×10^{-3}	35.73

All the values are representative for three independent protein preparations.

Standard curve

Volume (μL)	Absorbance (510 nm)	Concentration (mM)
0	0	0
10	0.204	1.785×10^{-2}
20	0.414	3.571×10^{-2}
30	0.622	5.357×10^{-2}
40	0.828	7.143×10^{-2}
50	1.028	8.929×10^{-2}

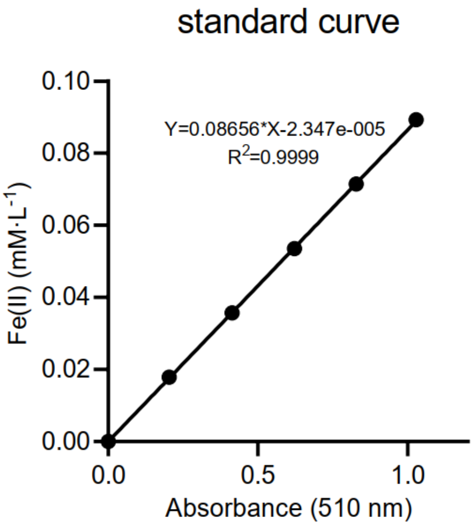

Supplement: Supplementary file 21 — Supplementary Table S4 [file 41422_2022_620_MOESM21_ESM.pdf]
