# Supplementary material for: Crystal structure and catalytic mechanism of the MbnBC holoenzyme required for methanobactin biosynthesis
Source: Cell Res. 2022 Feb 2;32(3):302–14. doi: 10.1038/s41422-022-00620-2 (PMC8888699; doi:10.1038/s41422-022-00620-2)
Supplement: Supplementary file 22 — Supplementary Table S5 [file 41422_2022_620_MOESM22_ESM.pdf]

Table S5. Synthesized peptides information

Peptides	Sequences	Purity (%)
**MbnA and variants form *Vibrio caribbenthicus* BAA-2122 (Group V)**		
VcMbnA	MKNDKKVVVKVKDKEMTCGAFNK	95
VcMbnA^{C18S}	MKNDKKVVVKVKDKEMTSGAFNK	95
VcMbnA, leader peptide	MKNDKKVVVKVKDKEM	95
VcMbnA, core peptide	TCGAFNK	95
**MbnA and variants from *Methylosinus trichosporium* OB3b (Group I)**		
MtMbnA	MTVKIAQKKVLPVIGRAAALCGSCYPCSCM	95
**MbnA and variants from *Rugamonas rubra* ATCC 43154 (Group III)**		
RrMbnA	MKIVIVKKVEIQVAGRTGMRCASSCGAKS	<80%
**MbnA and variants from *Methylocystis* sp.SC2 (Group II)**		
MsMbnA (Group II)	MTIRIAKRITLNVIGRAGAMCASTCAATNG	95
**MbnA and variants from *Azospirillum* sp. B510 (Group III)**		
AsMbnA (Group III)	MTIKIAKKQTLSVAGRAGACCGSCCAPVGVN	95
**MbnA and variants from *Gluconacetobacter* sp. SXCC-1 (Group IV)**		
GsMbnA (Group IV)	MAITITILKTKQISVPVRAGLQCGSGVCGYNA	95

All the peptides were verified by HPLC and mass spectrometry.
