# Supplementary material for: Crystal structure and catalytic mechanism of the MbnBC holoenzyme required for methanobactin biosynthesis
Source: Cell Res. 2022 Feb 2;32(3):302–14. doi: 10.1038/s41422-022-00620-2 (PMC8888699; doi:10.1038/s41422-022-00620-2)
Supplement: Supplementary file 23 — Supplementary Table S6 [file 41422_2022_620_MOESM23_ESM.pdf]

## Table S6. Bacterial strains and plasmids used in this study

| Plasmids and strains | Genotype/phenotype | Reference/source |
|---|---|---|
| *Escherichia coli* BL21 (DE3) | F⁻ *ompT hsdS*$_B$*(r*$_B^-$ *m*$_B^-$*) dcm*$^+$ *gal λ(DE3 [lacI lacUV5-T7 gene1 ind1 sam7 nin5])* | Novagen |
| pET28b*::MsLW4 MbnA | Gene fragment containing the codon-optimized MsLW4 *mbnA* and a C-terminal TEV cleavable $His_6$ tag, $Kan^R$ | This study |
| pET28b*::MsPW1 MbnA | Gene fragment containing the codon-optimized MsPW1 *mbnA* and a C-terminal TEV cleavable $His_6$ tag, $Kan^R$ | This study |
| pET28b*::MtOB3b MbnA | Gene fragment containing the codon-optimized MtOB3b *mbnA* and a C-terminal TEV cleavable $His_6$ tag, $Kan^R$ | This study |
| pET28b*::MhCSC1* MbnA | Gene fragment containing the codon-optimized MhCSC1* *mbnA* and a C-terminal TEV cleavable $His_6$ tag, $Kan^R$ | This study |
| pET28b*::MrSV97T MbnA | Gene fragment containing the codon-optimized MrSV97T *mbnA* and a C-terminal TEV cleavable $His_6$ tag, $Kan^R$ | This study |
| pET28b*::MsLW3(II) MbnA | Gene fragment containing the codon-optimized MsLW3(II) *mbnA* and a C-terminal TEV cleavable $His_6$ tag, $Kan^R$ | This study |
| pET28b*::MsR-45379(II) MbnA | Gene fragment containing the codon-optimized MsR-45379(II) *mbnA* and a C-terminal TEV cleavable $His_6$ tag, $Kan^R$ | This study |
| pET28b*::PeDSM17835 MbnA | Gene fragment containing the codon-optimized PeDSM17835 *mbnA* and a C-terminal TEV cleavable $His_6$ tag, $Kan^R$ | This study |
| pET28b*::RrATCC 43154 MbnA | Gene fragment containing the codon-optimized RrATCC 43154 *mbnA* and a C-terminal TEV cleavable $His_6$ tag, $Kan^R$ | This study |
| pET28b*::GsSXCC-1 MbnA | Gene fragment containing the codon-optimized GsSXCC-1 *mbnA* and a C-terminal TEV cleavable $His_6$ tag, $Kan^R$ | This study |
| pET28b*::VcBAA-2122 MbnA | Gene fragment containing the codon-optimized VcBAA-2122 *mbnA* and a C-terminal TEV cleavable $His_6$ tag, $Kan^R$ | This study |
| pET28b*::RrATCC 43154 MbnAno | Gene fragment containing the codon-optimized RrATCC 43154 *mbnA* with no tag, $Kan^R$ | This study |
| pET28b*::RrATCC 43154 $MbnA^{C21S, C25S}$ no | pET28b*: RrATCC 43154 MbnAno with C21S and C25S mutation in *mbnA* | This study |
| pET-28b | One cloning site; F1 ori, $Kan^R$ | Novagen |
| pET-Duet-1 | Two cloning sites; F1 ori, $Amp^R$ | Novagen |
| pET-Duet-1::MsLW4 MbnBC | Gene fragment containing the codon-optimized | This study |

| | | |
|---|---|---|
| | MsLW4 *mbnB* gene into CDS-2 of pET-Duet-1; *mbnC* gene from the same operon and an N-terminal $His_6$ tag into CDS-1. | |
| pET-Duet-1::MsPW1 MbnBC | Gene fragment containing the codon-optimized MsPW1 *mbnB* gene into CDS-2 of pET-Duet-1; *mbnC* gene from the same operon and an N-terminal $His_6$ tag into CDS-1. | This study |
| pET-Duet-1::MtOB3b MbnBC | Gene fragment containing the codon-optimized MtOB3b *mbnB* gene into CDS-2 of pET-Duet-1; *mbnC* gene from the same operon and an N-terminal $His_6$ tag into CDS-1. | This study |
| pET-Duet-1::MhCSC1 MbnBC | Gene fragment containing the codon-optimized MhCSC1 *mbnB* gene into CDS-2 of pET-Duet-1; *mbnC* gene from the same operon and an N-terminal $His_6$ tag into CDS-1. | This study |
| pET-Duet-1::MrSV97T MbnBC | Gene fragment containing the codon-optimized MrSV97T *mbnB* gene into CDS-2 of pET-Duet-1; *mbnC* gene from the same operon and an N-terminal $His_6$ tag into CDS-1. | This study |
| pET-Duet-1::MsLW3(II) MbnBC | Gene fragment containing the codon-optimized MsLW3(II) *mbnB* gene into CDS-2 of pET-Duet-1; *mbnC* gene from the same operon and an N-terminal $His_6$ tag into CDS-1. | This study |
| pET-Duet-1::MsR-45379(II) MbnBC | Gene fragment containing the codon-optimized MsR-45379(II) *mbnB* gene into CDS-2 of pET-Duet-1; *mbnC* gene from the same operon and an N-terminal $His_6$ tag into CDS-1. | This study |
| pET-Duet-1::PeDSM17835 MbnBC | Gene fragment containing the codon-optimized PeDSM17835 *mbnB* gene into CDS-2 of pET-Duet-1; *mbnC* gene from the same operon and an N-terminal $His_6$ tag into CDS-1. | This study |
| pET-Duet-1::RrATCC 43154 MbnBC | Gene fragment containing the codon-optimized RrATCC 43154 *mbnB* gene into CDS-2 of pET-Duet-1; *mbnC* gene from the same operon and an N-terminal $His_6$ tag into CDS-1. | This study |
| pET-Duet-1::GsSXCC-1 MbnBC | Gene fragment containing the codon-optimized GsSXCC-1 *mbnB* gene into CDS-2 of pET-Duet-1; *mbnC* gene from the same operon and an N-terminal $His_6$ tag into CDS-1. | This study |
| pET-Duet-1::VcBAA-2122 MbnBC | Gene fragment containing the codon-optimized VcBAA-2122 *mbnB* gene into CDS-2 of pET-Duet-1; *mbnC* gene from the same operon and an N-terminal $His_6$ tag into CDS-1. | This study |
| pET-Duet-1::MtOB3b $MbnB^{D241A}C$ | MtOB3b MbnBC with D241A mutation in *mbnB* | This study |

| | | |
|---|---|---|
| pET-Duet-1::MtOB3b MbnB$^{D241E}$C | MtOB3b MbnBC with D241E mutation in *mbnB* | This study |
| pET-Duet-1::MtOB3b MbnB$^{D241N}$C | MtOB3b MbnBC with D241N mutation in *mbnB* | This study |
| pET-Duet-1::MtOB3b MbnB$^{H54A}$C | MtOB3b MbnBC with H54A mutation in *mbnB* | This study |
| pET-Duet-1::MtOB3b MbnB$^{H90S}$C | MtOB3b MbnBC with H90S mutation in *mbnB* | This study |
| pET-Duet-1::MtOB3b MbnB$^{E133A}$C | MtOB3b MbnBC with E133A mutation in *mbnB* | This study |
| pET-Duet-1::MtOB3b MbnB$^{D163S}$C | MtOB3b MbnBC with D163S mutation in *mbnB* | This study |
| pET-Duet-1::MtOB3b MbnB$^{N166A}$C | MtOB3b MbnBC with N166A mutation in *mbnB* | This study |
| pET-Duet-1::MtOB3b MbnB$^{H192A}$C | MtOB3b MbnBC with H192A mutation in *mbnB* | This study |
| pET-Duet-1::MtOB3b MbnB$^{D208A}$C | MtOB3b MbnBC with D208A mutation in *mbnB* | This study |
| pET-Duet-1::MtOB3b MbnB$^{E239A}$C | MtOB3b MbnBC with E239A mutation in *mbnB* | This study |
| pET-Duet-1::RrATCC 43154 MbnB$^{D242A}$C | RrATCC 43154 MbnBC with D242A mutation in *mbnB* | This study |
| pET-Duet-1::RrATCC 43154 MbnB$^{D242E}$C | RrATCC 43154 MbnBC with D242E mutation in *mbnB* | This study |
| pET-Duet-1::RrATCC 43154 MbnB$^{D242N}$C | RrATCC 43154 MbnBC with D242N mutation in *mbnB* | This study |
| pET-Duet-1::RrATCC 43154 MbnB$^{H54A}$C | RrATCC 43154 MbnBC with H54A mutation in *mbnB* | This study |
| pET-Duet-1::RrATCC 43154 MbnB$^{H90S}$C | RrATCC 43154 MbnBC with H90S mutation in *mbnB* | This study |
| pET-Duet-1::RrATCC 43154 MbnB$^{E134A}$C | RrATCC 43154 MbnBC with E134A mutation in *mbnB* | This study |
| pET-Duet-1::RrATCC 43154 MbnB$^{D164S}$C | RrATCC 43154 MbnBC with D164S mutation in *mbnB* | This study |
| pET-Duet-1::RrATCC 43154 MbnB$^{N167A}$C | RrATCC 43154 MbnBC with N167A mutation in *mbnB* | This study |
| pET-Duet-1::RrATCC 43154 MbnB$^{H193A}$C | RrATCC 43154 MbnBC with H193A mutation in *mbnB* | This study |
| pET-Duet-1::RrATCC 43154 MbnB$^{D209A}$C | RrATCC 43154 MbnBC with D209A mutation in *mbnB* | This study |
| pET-Duet-1::RrATCC 43154 MbnB$^{E240A}$C | RrATCC 43154 MbnBC with E240A mutation in *mbnB* | This study |
| pET-Duet-1::RrATCC 43154 MbnBC$^{\triangle NTD28}$ | RrATCC 43154 MbnBC with N-terminal 1-28 residues deletion in *mbnC* | This study |
| pET-Duet-1::RrATCC 43154 MbnBC$^{\triangle 151\text{-}156}$ | RrATCC 43154 MbnBC with 151-156 residues deletion in *mbnC* | This study |
| pET-Duet-1::VcBAA-2122 MbnB$^{D240A}$C | VcBAA-2122 MbnBC with D240A mutation in *mbnB* | This study |
| pET-Duet-1::VcBAA-2122 MbnB$^{D240E}$C | VcBAA-2122 MbnBC with D240E mutation in *mbnB* | This study |
| pET-Duet-1::VcBAA-2122 MbnB$^{D240N}$C | VcBAA-2122 MbnBC with D240N mutation in *mbnB* | This study |
| pET-Duet-1::VcBAA-2122 MbnB$^{H55A}$C | VcBAA-2122 MbnBC with H55A mutation in *mbnB* | This study |
| pET-Duet-1::VcBAA-2122 MbnB$^{H91S}$C | VcBAA-2122 MbnBC with H91S mutation in *mbnB* | This study |
| pET-Duet-1::VcBAA-2122 MbnB$^{E135A}$C | VcBAA-2122 MbnBC with E135A mutation in *mbnB* | This study |
| pET-Duet-1::VcBAA-2122 MbnB$^{D165S}$C | VcBAA-2122 MbnBC with D165S mutation in *mbnB* | This study |
| pET-Duet-1::VcBAA-2122 MbnB$^{N168A}$C | VcBAA-2122 MbnBC with N168A mutation in *mbnB* | This study |
| pET-Duet-1::VcBAA-2122 MbnB$^{H194A}$C | VcBAA-2122 MbnBC with H194A mutation in *mbnB* | This study |
| pET-Duet-1::VcBAA-2122 MbnB$^{D209A}$C | VcBAA-2122 MbnBC with D209A mutation in *mbnB* | This study |
| pET-Duet-1::VcBAA-2122 MbnB$^{E238A}$C | VcBAA-2122 MbnBC with E238A mutation in *mbnB* | This study |
| pET-Duet-1::VcBAA-2122 MbnBC$^{\triangle NTD12}$ | VcBAA-2122 MbnBC with N-terminal 1-12 residues deletion in *mbnC* | This study |
| pET-Duet-1::VcBAA-2122 MbnBC$^{\triangle 131\text{-}136}$ | VcBAA-2122 MbnBC with 131-136 residues deletion in *mbnC* | This study |
