# Supplementary material for: Crystal structure and catalytic mechanism of the MbnBC holoenzyme required for methanobactin biosynthesis
Source: Cell Res. 2022 Feb 2;32(3):302–14. doi: 10.1038/s41422-022-00620-2 (PMC8888699; doi:10.1038/s41422-022-00620-2)
Supplement: Supplementary file 24 — Supplementary Table S7 [file 41422_2022_620_MOESM24_ESM.pdf]

Table S7. List of primers used in this study

	Forward primer	Reverse primer
**Construction of *mbnA* expression vectors**		
pET28b*-MsLW4 MbnA	F1:CGGATAACAATTCCCCTCTAGAAATAATTTTGTTTAACTTTAAGAAGGAGATATACC;	R1:CGGCAGGATCTCTTTTTTAACCACTTTGATGGTCATGGTATATCTCCTTCTTAAAGT;
	F2:AAAAGAGATCCTGCCGGTAATTGGTCGTGTTCAGGCCATGTGTGCTTGCAACCC;	R2:CTCACAAGTACCGCACCACGGCGGGTTGCAAGCACACA;
	F3:GTGCGGTACTTGTGAGAACCTGTACTTCCAAGGGCATCATCATCATCATCA	R3:TGGTGGTGGTGGTGCTCGAGTCAGTGATGATGATGATGATGCC
pET28b*-MsPW1 MbnA	F1:CGGATAACAATTCCCCTCTAGAAATAATTTTGTTTAACTTTAAGAAGGAGATATACC;	R1:GCAGAACTTCTTTTTTGGCAATTTTGATAGCCATGGTATATCTCCTTCTTAA;
	F2:AAAAAAGAAGTTCTGCCGGTCGTGGGCCGTCTGGGTGCTATGTGCAGCTCCTGT;	R2:CGGGCACAGCGGACCACACATCGGACAGGAGCTGCACATA;
	F3:GGTCCGCTGTGCCCGGAGAACCTGTACTTCCAAGGGCATCATCATCATCAT	R3:TGGTGGTGGTGGTGCTCGAGTCAGTGATGATGATGATGATGCC
pET28b*-MtOB3b MbnA	F1:TGAGCGGATAACAATTCCCCTCTAGAAATAATTTTGTTTAACTTTAAGAA;	R1:GAGCAATCTTGACAGTCATGGTATATCTCCTTCTTAAAGTTAAACAAAAT;
	F2:CATGACTGTCAAGATTGCTCAGAAGAAAGTCCTTCCGGTCATCGGTCGCG;	R2:ACGGATAGCACGAACCGCAAAGCGCAGCCGCGCGACCGATGACCGGAAGG;
	F3:GTTCGTGCTATCCGTGCAGCTGCATGGAGAACCTGTACTTCCAAGGGCATCATCAT	R3:TCTCAGTGGTGGTGGTGGTGGTGCTCGAGTCAGTGATGATGATGATGATG
pET28b*-MhCSC1* MbnA	F1:CGGATAACAATTCCCCTCTAGAAATAATTTTGTTTAACTTTAAGAAGGAGATATACC;	R1:GATACGTTTCGCGATACGAATGGTCATGGTATATCTCCTTCTT;
	F2:ATCGCGAAACGTATCACCCTGAACGTAGTTGGTCGTGCTGGTGCTATGTG;	R2:CGTTGGTCGCGGCACACGTAGACGCGCACATAGCACCAGCA;
	F3:TGCCGCGACCAACGGCGAGAACCTGTACTTCCAAGGGCATCATCATCATC	R3:TGGTGGTGGTGGTGCTCGAGTCAGTGATGATGATGATGATGCC
pET28b*-MrSV97T MbnA	F1:CGGATAACAATTCCCCTCTAGAAATAATTTTGTTTAACTTTAAGAAGGAGATATACC;	R1:AGAGTGATACGTTTTGCGATACGGATGGTCATGGTATATCTCCTTCTTA;
	F2:GCAAAACGTATCACTCTGAACGTTATCGGTCGTGCATCTGC	R2:GTTCTCGCCGTTAGTAGCCGCGCAGGTGGATGCGCA

	CCGTTGCG;<br>F3:GCTACTAACGGCGAGAACCTGTACTTCCAAGGGCATCATCATCATCAT	ACGGGCAGATG;<br>R3:TGGTGGTGGTGGTGCTCGAGTCAGTGATGATGATGATGATGCC
pET28b*-MsLW3(II) MbnA	F1:CGGATAACAATTCCCCTCTAGAAATAATTTTGTTTAACTTTAAGAAGGAGATATACC;<br>F2:AAACGTACTACCCTGGTTGTTAACGGCCGTTCTGGTGCAGATTG;<br>F3:ACCGCCTGCTGGGGCGAGAACCTGTACTTCCAAGGGCATCATCATCATCA	R1:CAGGGTAGTACGTTTTACGATGTTGATAGCCATGGTATATCTCCTTCTTA;<br>R2:CCCCAGCAGGCGGTACCGCAATCTGCACCAGAA;<br>R3:TGGTGGTGGTGGTGCTCGAGTCAGTGATGATGATGATGATGCC
pET28b*-MsR-45379(II) MbnA	F1:CGGATAACAATTCCCCTCTAGAAATAATTTTGTTTAACTTTAAGAAGGAGATATACC;<br>F2:CGTTAAACGTACCACTCTGGTCGTTAACGGTCGTAGCGGCGCAGACTGC;<br>F3:GCATGTTGGGGCGAGAACCTGTACTTCCAAGGGCATCATCATCATC	R1:GAGTGGTACGTTTAACGATGTTGATAGCCATGGTATATCTCCTTCTTA;<br>R2:CTCGCCCCAACATGCAGTGCCGCAGTCTGCGCCGCT;<br>R3:TGGTGGTGGTGGTGCTCGAGTCAGTGATGATGATGATGATGCC
pET28b*-PeDSM17835 MbnA	F1:CGGATAACAATTCCCCTCTAGAAATAATTTTGTTTAACTTTAAGAAGGAGATATACC;<br>F2:GAAACATACCCTGCAGATCGCAGGTCGTGCTGGTGCATGTTGTGCGA;<br>F3:GCACCGCTGGGTGTTAACGAGAACCTGTACTTCCAAGGGCATCATCATCATC	R1:TCTGCAGGGTATGTTTCTTCGCGATTTTGATGGACATGGTATATCTCCTTCT;<br>R2:AACACCCAGCGGTGCGCAGCAGCTCGCACAACATGCACC;<br>R3:TGGTGGTGGTGGTGCTCGAGTCAGTGATGATGATGATGATGCC
pET28b*-RrATCC 43154 MbnA	F1:CGGATAACAATTCCCCTCTAGAAATAATTTTGTTTAACTTTAAGAAGGAGATATACC;<br>F2:AAAAAGTGGAGATTCAGGTTGCAGGCCGTACCGGCATGCGT;<br>F3:TGTGGTGCAAAAAGCGAGAACCTGTACTTCCAAGGGCATCATCATCATCA	R1:CTGAATCTCCACTTTTTTAACGATCACGATTTTCATGGTATATCTCCTTCTTA;<br>R2:GCTTTTTGCACCACAAGAAGAAGCGCAACGCATGCCGGTACG;<br>R3:TGGTGGTGGTGGTGCTCGAGTCAGTGATGATGATGATGATGCC
pET28b*-RrATCC 43154 MbnAno	F1:CGGATAACAATTCCCCTCTAGAAATAATTTTGTTTAACTTTAAGAAGGAGATATACC;<br>F2:AAAAAGTGGAGATTCAGGT	R1:CTGAATCTCCACTTTTTTAACGATCACGATTTTCATGGTATATCTCCTTCTTA;<br>R2:GCTTTTTGCACCACAAG

	TGCAGGCCGTACCGGCATGCGT;<br>F3:TGTGGTGCAAAAAGCTAAGAGAACCTGTACTTCCAAGGGCATCATCATCATCA	AAGAAGCGCAACGCATGCCGGTACG;<br>R3:TGGTGGTGGTGGTGCTCGAGTCAGTGATGATGATGATGATGCC
pET28b*-GsSXCC-1 MbnA	F1:CGGATAACAATTCCCCTCTAGAAATAATTTTGTTTAACTTTAAGAAGGAGATATACC;<br>F2:TCCTGAAAACCAAACAGATCTCTGTGCCGGTCCGTGCCGGCCTGCAGTGCGGC;<br>F3:TGGCTACAACGCGGAGAACCTGTACTTCCAAGGGCATCATCATCATCAT	R1:TGTTTGGTTTTCAGGATGGTAATGGTAATAGCCATGGTATATCTCCTTCTTAA;<br>R2:TCCGCGTTGTAGCCACAAACGCCGGAGCCGCACTGCAGGCC;<br>R3:TGGTGGTGGTGGTGCTCGAGTCAGTGATGATGATGATGATGCC
pET28b*-VcBAA-2122 MbnA	F1:TGAGCGGATAACAATTCCCCTCTAGAAATAATTTTGTTTAACTTTAAGAA;<br>F2:ATGAAGAACGACAAGAAGGTGGTGGTGAAGGTGAAGGACAAGGAGATGAC;<br>F3:TTCAATAAAGAGAACCTGTACTTCCAAGGGCATCATCATCATCATCACTG	R1:ACCTTCTTGTCGTTCTTCATGGTATATCTCCTTCTTAAAGTTAAACAAAA;<br>R2:TACAGGTTCTCTTTATTGAAGGCGCCGCACGTCATCTCCTTGTCCTTCAC;<br>R3:TCTCAGTGGTGGTGGTGGTGGTGCTCGAGTCAGTGATGATGATGATGATG
**Mutagenesis of *mbnA***		
pET28b*-RrATCC 43154 MbnA^{C21,25S}no	TAGCTTTTTGCACCACTAGAAGAAGCGCTACGCATGCCGGTACGG	CCGTACCGGCATGCGTAGCGCTTCTTCTAGTGGTGCAAAAAGCTA
**Mutagenesis of *mbnB***		
pET-Duet-1-MtOB3b MbnB^{H54A}C	TGCCCGGTGGCCTTCGCAATTATGCTGAGCAAG	CTTGCTCAGCATAATTGCGAAGGCCACCGGGCA
pET-Duet-1-MtOB3b MbnB^{H90S}C	GTTTACGTGAGCGATAGCATTTTATACTTCACC	GGTGAAGTATAAAATGCTATCGCTCACGTAAAC
pET-Duet-1-MtOB3b MbnB^{E133A}C	ACCCGTTTATATTTAGCAAACTACCCGAGCATC	GATGCTCGGGTAGTTTGCTAAATATAAACGGGT
pET-Duet-1-MtOB3b MbnB^{D163S}C	GTGGGCGTGCTGTTTAGCGCCAGCAATGCCATT	AATGGCATTGCTGGCGCTAAACAGCACGCCCAC
pET-Duet-1-MtOB3b MbnB^{N166A}C	CTGTTTGATGCCAGCGCAGCCATTTGCGCCCAG	CTGGGCGCAAATGGCTGCGCTGGCATCAAACAG
pET-Duet-1-MtOB3b MbnB^{H192A}C	ACCACCCGCCATTTTGCAGTGGCTGGTTATGGC	GCCATAACCAGCCACTGCAAAATGGCGGGTGGT
pET-Duet-1-MtOB3b MbnB^{D208A}C	CCGCGCGTGAAGGCCGCAACCCATGACCGTGAG	CTCACGGTCATGGGTTGCGGCCTTCACGCGCGG
pET-Duet-1-MtOB3b MbnB^{E239A}C	GCCACCATTACCTACGCACGCGATTTCGATATC	GATATCGAAATCGCGTGCGTAGGTAATGGTGGC

pET-Duet-1-MtOB3b MbnB^{D241A}C	ATTACCTACGAACGCGCATTCGATATCGATTAT	ATAATCGATATCGAATGCGCGTTCGTAGGTAAT
pET-Duet-1-MtOB3b MbnB^{D241E}C	ATTACCTACGAACGCGAATTCGATATCGATTAT	ATAATCGATATCGAATTCGCGTTCGTAGGTAAT
pET-Duet-1-MtOB3b MbnB^{D241N}C	ATTACCTACGAACGCAACTTCGATATCGATTAT	ATAATCGATATCGAAGTTGCGTTCGTAGGTAAT
pET-Duet-1-RrATCC 43154 MbnB^{H54A}C	AAAGCGGCTAAACATAATAGCAAAGCCCACCGGCACATCA	TGATGTGCCGGTGGGCTTTGCTATTATGTTTAGCCGCTTT
pET-Duet-1-RrATCC 43154 MbnB^{H90S}C	GATGGCTAAAATACGCAATACTATCGCTCACATACAGCGGGC	GCCCGCTGTATGTGAGCGATAGTATTGCGTATTTTAGCCATC
pET-Duet-1-RrATCC 43154 MbnB^{E134A}C	CACAATGCTCGGATAGTTTGCAAAATGAATGGTCTGACC	GGTCAGACCATTCATTTTGCAAACTATCCGAGCATTGTG
pET-Duet-1-RrATCC 43154 MbnB^{D164S}C	CGCGTTGCTAACACTAAACAGCACGCCCGCGC	GCGCGGGCGTGCTGTTTAGTGTTAGCAACGCG
pET-Duet-1-RrATCC 43154 MbnB^{N167A}C	CACGCGCACACCGCGGCGCTAACATCAAACAGC	GCTGTTTGATGTTAGCGCCGCGGTGTGCGCGTG
pET-Duet-1-RrATCC 43154 MbnB^{H193A}C	CCGCCCACAGCAAAATGGCTCGCGCCCG	CGGGCGCGAGCCATTTTGCTGTGGGCGG
pET-Duet-1-RrATCC 43154 MbnB^{D209A}C	GCGATCATGGGTAGCCACGGTCACGCC	GGCGTGACCGTGGCTACCCATGATCGC
pET-Duet-1-RrATCC 43154 MbnB^{E240A}C	TGTTTTCATCGCGTGCATAGGTAATGGTCGCGC	GGCGCGACCATTACCTATGCACGCGATGAAAACA
pET-Duet-1-RrATCC 43154 MbnB^{D242A}C	CGTCAATGTCAATGTTTTCAGCGCGTTCATAGGTAATGGTC	GACCATTACCTATGAACGCGCTGAAAACATTGACATTGACG
pET-Duet-1-RrATCC 43154 MbnB^{D242E}C	CGTCAATGTCAATGTTTTCCTCGCGTTCATAGGTAATGG	CCATTACCTATGAACGCGAGGAAAACATTGACATTGACG
pET-Duet-1-RrATCC 43154 MbnB^{D242N}C	GTCAATGTCAATGTTTTCATTGCGTTCATAGGTAATGGTCG	CGACCATTACCTATGAACGCAATGAAAACATTGACATTGAC
pET-Duet-1∷RrATCC 43154 MbnBC$^{\triangle NTD28}$	CATCATCACCACAGCCAAGGATCCCCGGCGGCGCGCGGCGAT	CATTATGCGGCCGCAAGCTTTTAGCGCGGCAG
pET-Duet-1∷RrATCC 43154 MbnBC$^{\triangle 151\text{-}156}$	GATTGGATTATCGGCGCGGCGGGCCGCCGCGTGGAA	TTCCACGCGGCGGCCCGCCGCGCCGATAATCCAATC
pET-Duet-1-VcBAA-2122 MbnB^{H55A}C	CGCCCGTGTGCCTTCGCAATCATGAACAGCCAG	CTGGCTGTTCATGATTGCGAAGGCACACGGGCG
pET-Duet-1-VcBAA-2122 MbnB^{H91S}C	ATTTACATCAGCGATAGCATCGGCAAGTTCTAT	ATAGAACTTGCCGATGCTATCGCTGATGTAAAT
pET-Duet-1-VcBAA-2122 MbnB^{E135A}C	GGTAAGCTGCTGCTGGCAAACTACCCGAGCATT	AATGCTCGGGTAGTTTGCCAGCAGCAGCTTACC
pET-Duet-1-VcBAA-2122 MbnB^{D165S}C	TGTGGTTTACTGTTCAGCATCAGTAACGCCTTC	GAAGGCGTTACTGATGCTGAACAGTAAACCACA

pET-Duet-1-VcBAA-2122 MbnB^{N168A}C	CTGTTCGACATCAGTGCAGCCTTCATCGCCGAG	CTCGGCGATGAAGGCTGCACTGATGTCGAACAG
pET-Duet-1-VcBAA-2122 MbnB^{H194A}C	CACTGCCAGCACTTCGCAATTGCCGGCTTTGAA	TTCAAAGCCGGCAATTGCGAAGTGCTGGCAGTG
pET-Duet-1-VcBAA-2122 MbnB^{D209A}C	AACCAGTTTTTAGTGGCAACCCATAGTCAGTGC	GCACTGACTATGGGTTGCCACTAAAAACTGGTT
pET-Duet-1-VcBAA-2122 MbnB^{H211A}C	TTTTTAGTGGATACCGCAAGTCAGTGCATCGAA	TTCGATGCACTGACTTGCGGTATCCACTAAAAA
pET-Duet-1-VcBAA-2122 MbnB^{E238A}C	GCCACCATTAGCGTGGCACGCGACGAAAACTTC	GAAGTTTTCGTCGCGTGCCACGCTAATGGTGGC
pET-Duet-1-VcBAA-2122 MbnB^{D240A}C	ATTAGCGTGGAGCGCGCAGAAAACTTCGATGTT	AACATCGAAGTTTTCTGCGCGCTCCACGCTAAT
pET-Duet-1-VcBAA-2122 MbnB^{D240N}C	ATTAGCGTGGAGCGCAACGAAAACTTCGATGTT	AACATCGAAGTTTTCGTTGCGCTCCACGCTAAT
pET-Duet-1-VcBAA-2122 MbnB^{D240E}C	ATTAGCGTGGAGCGCGAAGAAAACTTCGATGTT	AACATCGAAGTTTTCTTCGCGCTCCACGCTAAT
pET-28b-VcBAA-2122 MbnB	GTGCCGCGCGGCAGCCATATGAATGTGGGTATTAACTGGAGCGGCC	GTGGTGGTGGTGGTGCTCGAGACGGGTATCACGACCGTTAC
pET-Duet-1::VcBAA-2122 MbnBC$^{\triangle NTD12}$	TCATCACCACAGCCAGGATCCGATGTCGGCTAAGCCGCTAACT	GTGGTGGTGGTGGTGCTCGAGACGGGTATCACGACCGTTAC
pET-Duet-1::VcBAA-2122 MbnBC$^{\triangle 131\text{-}136}$	F1:TCATCACCACAGCCAGGATCCGATGGAAGAGATACTAGATAGAATAATT<br>F2:GTTTTTGGAGGAAATGAATTTCGTGAGGTA	R1:TACCTCACGAAATTCATTTCCTCCAAAAAC<br>R2:GCATTATGCGGCCGCAAGCTTTCATAGTTTTTCACCCATAGTAC
