# Supplementary material for: Crystal structure and catalytic mechanism of the MbnBC holoenzyme required for methanobactin biosynthesis
Source: Cell Res. 2022 Feb 2;32(3):302–14. doi: 10.1038/s41422-022-00620-2 (PMC8888699; doi:10.1038/s41422-022-00620-2)
Supplement: Supplementary file 25 — Supplementary Table S8 [file 41422_2022_620_MOESM25_ESM.pdf]

Table S8. The recipe of minimal nutrient M9 medium

Reagent	Content (Final concentration in 1L)
Na_2HPO_4	8.86 g
KH_2PO_4	3.9 g
NaCl	0.65 g
NH_4Cl	1.3 g
Glucose	5 %
YNB medium	0.65 %
**Amino acids**	
L-Met	50 mg
L-Leu	50 mg
L-Ile	50 mg
L-Val	50 mg
L-Lys	100 mg
L-Thr	100 mg
L-Phe	100 mg
ddH_2O	Make up the volume

YNB medium was from BD Difco. And the other regents were purchased from MilliporeSigma (Darmstadt Germany).

We introduced excess ^{57}Fe into the bacteria using M9 minimal medium addition of amino acids, which avoided the effect of ^{56}Fe within the original LB medium. The overnight starter culture was inoculated into the M9 medium supplemented with 0.3 mg/L freshly prepared ^{57}Fe (anaerobically dissolved under heat in hydrochloric acid). Growth was initiated with continuous oscillation at 37 °C, followed by addition of seven essential amino acids when the OD_{600} reached to 0.5-0.6. Then the flasks were cooled shift to 16 °C and addition of a final 0.1 mM IPTG for induction. Protein purification steps were conducted as the description of methods.
