# Supplementary material for: Crystal structure and catalytic mechanism of the MbnBC holoenzyme required for methanobactin biosynthesis
Source: Cell Res. 2022 Feb 2;32(3):302–14. doi: 10.1038/s41422-022-00620-2 (PMC8888699; doi:10.1038/s41422-022-00620-2)
Supplement: Supplementary file 26 — Supplementary Table S9 [file 41422_2022_620_MOESM26_ESM.pdf]

Table S9. List of reconstituted MbnBC and MbnABC complexes

Reconstitution	Transformation
MbnBC	
MsLW4 MbnBC	pET-Duet-1:: MsLW4 MbnBC
MsPW1 MbnBC	pET-Duet-1:: MsPW1 MbnBC
MtMbnBC	pET-Duet-1:: MtOB3b MbnBC
MhCSC1* MbnBC	pET-Duet-1:: MhCSC1* MbnBC
MrSV97T MbnBC	pET-Duet-1:: MrSV97T MbnBC
MsLW3(II) MbnBC	pET-Duet-1:: MsLW3(II) MbnBC
MsR-45379(II) MbnBC	pET-Duet-1:: MsR-45379(II) MbnBC
PeDSM17835 MbnBC	pET-Duet-1:: PeDSM17835 MbnBC
RrMbnBC	pET-Duet-1:: RrATCC 43154 MbnBC
GsSXCC-1 MbnBC	pET-Duet-1:: GsSXCC-1 MbnBC
VcMbnBC	pET-Duet-1:: VcBAA-2122 MbnBC
MbnABC	
MsLW4 MbnABC	pET-Duet-1:: MsLW4 MbnBC co-transformation with pET28b*::MsLW4 MbnA
MsPW1 MbnABC	pET-Duet-1:: MsPW1 MbnBC co-transformation with pET28b*::MsPW1 MbnA
MtMbnABC	pET-Duet-1::MtOB3b MbnBC co-transformation with pET28b*::MtOB3b MbnA
MhCSC1* MbnABC	pET-Duet-1::MhCSC1* MbnBC co-transformation with pET28b*::MhCSC1* MbnA
MrSV97T MbnABC	pET-Duet-1::MrSV97T MbnBC co-transformation with pET28b*::MrSV97T MbnA
MsLW3(II) MbnABC	pET-Duet-1::MsLW3(II) MbnBC co-transformation with pET28b*::MsLW3(II) MbnA
MsR-45379(II) MbnABC	pET-Duet-1::MsR-45379(II) MbnBC co-transformation with pET28b*::MsR-45379(II) MbnA
PeDSM17835 MbnABC	pET-Duet-1::PeDSM17835 MbnBC co-transformation with pET28b*::PeDSM17835 MbnA
RrMbnABC	pET-Duet-1::RrATCC 43154 MbnBC co-transformation with pET28b*::RrATCC 43154 MbnA
GsSXCC-1 MbnABC	pET-Duet-1::GsSXCC-1 MbnBC co-transformation with pET28b*::GsSXCC-1 MbnA
VcMbnABC	pET-Duet-1::VcBAA-2122 MbnBC co-transformation with pET28b*::VcBAA-2122 MbnA
